# Application of Proteomics and Metabonomics to Reveal the Molecular Basis of Atractylodis Macrocephalae Rhizome for Ameliorating Hypothyroidism Instead of Hyperthyroidism

**DOI:** 10.3389/fphar.2021.664319

**Published:** 2021-04-20

**Authors:** Jing Chen, Peiyuan Dou, Hang Xiao, Deqiang Dou, Xueying Han, Haixue Kuang

**Affiliations:** ^1^ College of Pharmacy, Liaoning University of Traditional Chinese Medicine, Da Lian, China; ^2^ Department of Pharmacy, Taizhou Hospital of Zhejiang Province Affiliated to Wenzhou Medical University, Linhai, Zhejiang, China; ^3^ College of Pharmacy, Heilongjiang University of Chinese Medicine, Harbin, Heilongjiang, China

**Keywords:** network pharmacology, atractylodis macrocephalae rhizome, hypothyroidism, hyperthyroidism, proteomics, metabonomics

## Abstract

As the treatments of diseases with Chinese herbs are holistic and characterized by multiple components, pathways, and targets, elucidating the efficacy of Chinese herbs in treating diseases, and their molecular basis, requires a comprehensive, network-based approach. In this study, we used a network pharmacology strategy, as well as *in vivo* proteomics and metabonomics, to reveal the molecular basis by which *Atractylodis macrocephalae* rhizome (AMR) ameliorates hypothyroidism. Eighteen main compounds from AMR and its fractions (volatile oil fraction, crude polysaccharides fraction, lactones fraction, oligosaccharide fraction, and atractyloside fraction) were identified by HPLC, and their targets were screened using the TCMSP database and Swiss Target Prediction. Disease targets were gathered from the TTD, CTD and TCMSP databases. Hub targets were screened by different plug-ins, such as Bisogene, Merge, and CytoNCA, in Cytoscape 3.7.1 software and analyzed for pathways by the DAVID database. Hypothyroidism and hyperthyroidism pharmacological models were established through systems pharmacology based on proteomic and metabolomic techniques. Finally, AMR and its fractions were able to ameliorate the hypothyroidism model to different degrees, whereas no significant improvements were noted in the hyperthyroidism model. The lactones fraction and the crude polysaccharides fraction were considered the most important components of AMR for ameliorating hypothyroidism. These amelioration effects were achieved through promoting substance and energy metabolism. In sum, the integrative approach used in this study demonstrates how network pharmacology, proteomics, and metabolomics can be used effectively to elucidate the efficacy, molecular basis, and mechanism of action of medicines used in TCM.

## Introduction

Traditional Chinese medicine (TCM) has been used to prevent, diagnose, and treat illness for thousands of years (Feng et al., 2006). Chinese herbs have multi-component, multi-path, and multi-target characteristics that can be used to holistically treat diseases (Pan et al., 2020; Wang et al., 2012). One of the major challenges facing the modernization of TCM is elucidating the molecular basis of medicines and the mechanisms underlying the efficacy of medicines used in TCM (Peng et al., 2018; Zhang et al., 2018). New methods are thus essential for exploring the efficacy of these medicines as well as characterizing their molecular basis. Network pharmacology is an emerging field based on the theoretical development of systems biology, multi-directional pharmacology, and omics that has been used to develop new strategies and methods to study new drugs (Zhai et al., 2018). The multi-level information associated with the “compound-target-pathway-disease” network provides insight into the workings of TCM by clarifying its multi-component and multi-target characteristics (Ru et al., 2014). Furthermore, this approach can be used to reveal the roles of TCM interventions in the illness network and predict the effects of pharmacodynamic components on certain key targets of diseases and their pathways, providing scientific evidence that clarifies the efficacy of the medicines used in TCM, their molecular basis, and their underlying mechanisms (Huang et al., 2018). Hence, network pharmacology has been widely used to reveal the efficacy of medicines, their molecular basis, and the mechanisms underlying the many components and targets involved in TCM.

Network pharmacology reveals the relationships between drugs and targets, targets and diseases, as well as between diseases themselves. Given that network pharmacology is based on the networks of computers and databases, the results of network pharmacology require verification with more realistic pharmacological modes. Proteomics and metabonomics have been used to discover biomarkers in biological systems and characterize the mechanisms underlying TCM (Liu and Guo, 2011; Pan et al., 2020; Zhang et al., 2020). Proteomics can be used to detect large-scale changes in proteins associated with diseases as well as cell metabolic processes, whereas metabolomics can be used to find changes in the metabolic pathways of an organism (Liu and Guo, 2011; Zhang et al., 2020). In other words, proteomics can reveal what is going to happen, and metabolomics can reveal what biological processes have occurred in organisms. These changes in proteins and metabolites detected by proteomics and metabonomics can then be used as biomarkers to predict and diagnose diseases (Bracewell-Milnes et al., 2017). Furthermore, these approaches have been widely used to evaluate the therapeutic effects and mechanisms of TCM in recent years (Buriani et al., 2012). Therefore, the development of a novel integrated approach consisting of network pharmacology, proteomics, and metabonomics that could be applied in compounds, core targets, and biomarkers screening, could be aided our ability to characterize the mechanisms underlying the efficacy of medicines used in TCM as well as their molecular basis.

Hyperthyroidism and hypothyroidism are two of the most common disorders of the endocrine system. Hyperthyroidism with excess thyroid hormones is often along with weight loss, reduced cholesterol levels, increased lipolysis, and gluconeogenesis and energy expenditure (Motomura and Brent, 1998; Obregon, 2008). In contrast, hypothyroidism with reduced thyroid hormones is associated with hypometabolism and characterized by reduced resting energy expenditure, weight gain, increased cholesterol levels, reduced lipolysis, and reduced gluconeogenesis (Meier and Kaplan, 2002). In TCM, hyperthyroidism results from *Yin* deficiency and hypothyroidism results from *Yang* deficiency. Chinese drugs are thought to ameliorate *Yin* deficiency or *Yang* deficiency and have been used to treat hyperthyroidism or hypothyroidism for thousands years (Ke et al., 2015; Zen et al., 2007). Recent studies have shown the remarkable efficacy of warming and invigorating drugs to hypothyroidism (Cheng et al., 2016). The literatures have demonstrated that *Atractylodes macrocephala Koidz*. rhizome (AMR) is among the most frequently prescribed medications in TCM for treating hypothyroidism (Shan et al., 2017). The pharmacological activity of AMR and its components have been previously explored (Chen et al., 2016; Wang F. et al., 2015). However, there are not any reports concerning the effects of AMR on hypothyroidism or hyperthyroidism. According to TCM theory, hypothyroidism belongs to a *cold* syndrome, while hyperthyroidism belongs to a *heat* syndrome. Chinese drugs with *warm* or *hot* natures are suitable for the treatment of *cold* syndromes, while drugs with *cool* or *cold* natures are suitable for the treatment of *heat* syndromes (Xiao et al., 2017). In TCM, AMR is a *warm* or *hot*-natured drug and primarily used for *cold* or *cool* syndromes. Up till now, AMR is the most widely used Chinese medicine to treat hypothyroidism rather than hyperthyroidism. It is still unclear whether the effect of AMR on hypothyroidism is a superior to hyperthyroidism. Furthermore, the mechanism underlying the efficacy of AMR and its molecular basis also remains unclear. Therefore, it is necessary to further study the effects of AMR on hypothyroidism and hyperthyroidism to provide a basis for treating hypothyroidism or hyperthyroidism.

Here, we developed a novel network pharmacology strategy to reveal how AMR ameliorates hypothyroidism combined with *in vivo* proteomics and metabonomics. This study not only helps clarify the molecular basis and mechanism of AMR in alleviating hypothyroidism but also contributes to explaining the mechanism of TCM theory, wherein Chinese drugs with *warm* or *hot* natures are suitable for the treatment of *cold* syndromes with lower energy metabolism, such as hypothyroidism, rather than *heat* syndromes. More generally, this study also provides effective tools for elucidating the efficacy of medicines, their molecular basis, and their underlying mechanisms.

## Materials and Methods

### Materials

Instruments and reagents used for indices measurements, proteomics analyses, and metabolomics analyses, as well as animal materials, plant materials, and the preparation of the splitted fractions of AMR, were described in the Supporting Materials S1.

### Network Construction

According to the our previous research (Chen et al., 2016), the molecular components of AMR were divided into the following fractions: volatile oil fraction (VOF), lactones fraction (LAF), atractyloside fraction (ATF), oligosaccharide fraction (OSF), and crude polysaccharide fraction (CPF). And our previous research showed that CPF primarily contained inulin-type polysaccharides (Lin et al., 2015). As network pharmacology was mainly used to screen the potential targets of small molecular compounds for elucidating the role and the underlying mechanism. Hence, we didn’t use network pharmacology to study polysaccharide and evaluate its pharmacology as well. A total of 18 compounds from VOF, LAF, ATF, and OSF were identified by HPLC (Supplementary Table S1). Potential targets of 18 compounds were obtained by the Swiss Target Prediction database (http://www.swisstargetprediction.ch/), STICH database (http://stitch.embl.de/), and the Traditional Chinese Medicine Systems Pharmacology (TCMSP) database. And hypothyroidism and hyperthyroidism targets were screened from the Therapeutic Target database (TTD) (http://bidd.nus.edu.sg/group/ttd/), Comparative Toxicogenomics database (CTD, http://ctdbase.org/), Integrative Pharmacology-based Research Platform of Traditional Chinese Medicine (TCMIP, http://www.tcmip.cn/), and TCMSP database. Compound-targets and disease-targets were standardized through the UniProt database (http://www.uniProt.org/). The plugins including Bisogene, Merge, and CytoNCA in Cytoscape in the Cytoscape 3.7.1 software (http://www.cytoscape.org/) were used to construct the compound-candidate targets network, protein-protein interaction (PPI) network and screen the core therapeutic targets of AMR. And KEGG pathway enrichment analyses of core targets were performed using DAVID database (http://david.abcc.ncifcrf.gov) to determine the key pathways of the core targets and to explore the candidate compounds of AMR and the mechanism by which it ameliorates hypothyroidism and hyperthyroidism.

### Establishment of the Pharmacological Model and Treatment

#### Hypothyroidism Model Establishment and Treatment

In reference to previous research from our lab, the hypothyroidism model rats were succesfully established by administering PTU (Xiao et al., 2017). Specific experimental details were provided below. Rats were randomly divided into eight groups (10 per group): CON group, hypo-MO group, hypo-WD group, hypo-VOF group, hypo-CPF group, hypo-LAF group, hypo-OSF group, and hypo-ATF group. The hypo-MO, hypo-WD, hypo-VOF, hypo-CPF, hypo-LAF, hypo-OSF, and hypo-ATF groups were administered PTU (intraperitoneally, 10 mg kg^−1^·d^−1^), while the CON group was administered the same volume of distilled water. After 4 h, each group was orally administered *i. g* as follows: CON group and hypo-MO group, purified water 1 ml·(100 g)^−1^; hypo-WD group, 6.6 g kg^−1^ water decoction; fractions groups (hypo-VOF, hypo-CPF, hypo-LAF, hypo-OSF, hypo-ATF), corresponding extracts of AMR with 6.6 g kg^−1^ crude herbs for 20 days.

#### Hyperthyroidism Model Establishment and Treatment

The implementation method refers to our previous study, the hyperthyroidism model rats were succesfully established by administering euthyrox (Xiao et al., 2016). Specific experimental details were provided below. Rats were randomly divided into eight groups (10 per group): CON group, Hype-MO group, Hype-WD group, Hype-VOF group, Hype-CPF group, Hype-LAF group, Hype-OSF group, and Hype-ATF group. The Hype-MO, Hype-WD, Hype-VOF, Hype-CPF, Hype-LAF, OSF, and Hype-ATF groups were administered *i. g* with euthyrox (240 μg kg^−1^·d^−1^), while the CON group was administered *i. g* with the same volume of distilled water. After 4 h, each group was orally administered *i. g* as follows: CON group and Hype-MO group, purified water 1 ml·(100 g)^−1^; Hype-WD group, 6.6 g kg^−1^ water decoction; fractions groups (Hype-VOF, Hype-CPF, Hype-LAF, Hype-OSF, Hype-ATF), corresponding extracts of AMR with 6.6 g kg^−1^ crude herbs for 15 days.

### Sample Collection and Preparation

Twelve hours after the final drug administration, urine samples were collected. Unrine was centrifuged at 12,000 rpm for 10 min and the supernatants was used for UHPLC-Q-TOF-MS/MS analysis. Then, blood was collected and serum was obtained following centrifugation at 2,500 rpm min^−1^ for 15 min. After euthanasia, liver dissections were performed immediately and washed with normal saline using for blood removal. Approximately 0.2 g of the liver tissue were obtained for proteomics analysis and frozen at -80 °C before use. In addition, approximately 0.1 g of the liver tissue were homogenized at a ratio of 1:9 (w/v) in a 0.9% saline solution, which was used to detect the Na^+^-K^+^-ATPase activity and frozen at -80°C before use.

### Index Measurement

Rectal temperature of the rats were measured by an electronic standard rectal thermometer at 4:00 pm of first, seventh, 14th, and 20th d in the hypothyroidism model and 1st, 5th, 10th, and 15th d in the hyperthyroidism model. And body weight of the rats were weighed by electronic scales at 8:00 am. The levels of TSH, T_3_ and T_4_ in serum, and activity of Na^+^-K^+^-ATPase in liver were measured using ELISA kits according to the manufacturer’s instructions.

### Metabolomic Analysis

#### Chromatography and Mass Spectrometry Conditions

Unrine was centrifuged at 12,000 rpm for 10 min and the supernatant was analyzed using UPLC-Q-TOF-MS/MS. Quality control (QC) samples were prepared by mixing an equal amount of each sample. UPLC/Q-TOF-MS/MS analysis was performed on an ACQUITY UPLC HSS T3 C18 column (2.1 × 100 mm, 1.7 μm, Waters Corp. Milford, United States). The injection volume was 4 μL, flow rate was 0.6 ml/min and the column temperature was 45°C. And the gradient elution was performed using a gradient of mobile phase A (0.1% formic acid in acetonitrile) and mobile phase B (0.1% formic acid in water) with the following time schedule: 0–8 min, 2–8% B; 8–10 min, 40–98% B; 10–12 min, 98 to 2% B; 12–14 min, 2% B separately; and curve, 6. Mass spectrometric analysis was performed on a Waters Xevo G2 QTOF quadrupole accelerated time-of-flight mass spectrometer (Waters Corp. Manchester, United States) in both positive (ESI^+^) and negative (ESI^-^) ionization modes.

#### Multivariate Statistical Analysis

The raw data of UPLC/Q-TOF-MS/MS were initially processed using MarkerLynx 4.1 software. The OPLS-DA and PLS-DA were used to identify the difference in metabolites. The metabolites with VIP >1 and *p* < 0.05 were used as candidate metabolites.

#### Metabolite Identification and Metabolic Annotation

Candidate metabolites were identified by comparing their retention times and mass spectra with those of the standards and the mass spectra data at METLIN database (http://metlin.scripps.edu/), HMDB database (http://www.hmdb.ca/), and KEGG database (http://www.genome.jp/kegg/). Pathway analysis was performed with the MetPA database (http://metpa.metabolomics.ca./MetPA/faces/Home.jsp).

#### iTRAQ Proteome Analysis

Our previous research has shown that *warm*-natured drugs could ameliorate diseases with *cold* syndrome by way of regulation on the metabolism of substance and energy of organism (Xiao et al., 2017). We speculated that regulation of substance and energy metabolism was one of the mechanisms of AMR in the treatment of hypothyroidism. Meanwhile, the activities of Na^+^-K^+^-ATPase in liver were significantly up-regulated in hypothyroidism rats by treating with AMR. In addition, the liver was the primary site for substance and energy metabolism. Hence, liver samples were collected for the iTRAQ proteome analysis, which was used to evaluate the effects of AMR and its fractions on hypothyroidism rats.

#### iTRAQ-Labeling

First, 100 mg liver samples were weighed and homogenized with 1,000 μL of protein extraction reagent on ice. The homogenate was placed in an ice bath for 20 min and then centrifuged under 10,000 g at 4°C for 10 min. Protein concentrations were detected by BCA kit. Further, the proteins from each group were denatured, reduced, and digested at room temperature according to the iTRAQ reagent reference guide. The tryptic peptides from the CON, hypo-MO, hypo-WD, hypo-LAF, hypo-VOF, hypo-OSF, hypo-ATF, and hypo-CPF groups were labeled with 8-plex iTRAQ reagents with different tags for 2 h at room temperature. The labeled peptides were then pooled together and dried by vacuum centrifugation.

#### High-pH Reverse-phase Liquid Chromatography Fractionation

The iTRAQ-labeled peptide mixtures were fractionated on an LC-2998 HPLC Pump system (Waters, United States). Peptides were redissolved in 150 μL of buffer A (25 mM HCOONH_4_ in H_2_O, pH 10) and loaded onto a 4.6 × 250 mm Ultremex SCX C18 column that contained 5 μm particles (Phenomenex). And the gradient elution was performed using a gradient of mobile phase A (25 mM HCOONH_4_ in H_2_O, pH 10) and mobile phase B (pH = 10 20 mM HCOONH_4_, 80% ACN) with the following time schedule: 0–5 min, 5% B; 5–30 min, 5–15% B; 30–45 min, 15–48% B; 45–46 min, 38–90% B; 46–54.5 min, 90% B; 54.5–55 min, 90 to 5% B; and 55–65 min, 5% B. The eluent was monitored by absorbance at 254 nm. A total of fifty fractions were collected at 1 min intervals from 6 to 55 min. Further, these fractions were combined into 10 fractions by merging fractions 1, 11, 21, 31, 41 and 51; fractions 2, 12, 22, 32, 42, 52; and so on. All ten fractions were dried by vacuum centrifugation and analyzed using LC-ESI-MS/MS analysis based on Triple TOF 5600.

#### LC-ESI-MS/MS Analysis based on Triple TOF 5600

iTRAQ-labeled peptide mixtures were performed using a Triple-TOF 5600 system (AB SCIEX, Massachusetts, United States). Peptides were loaded on a nanocolumn (75 μm × 150 mm) and nanotrap column (350 μm × 0.5 mm), and filled with ChromXP C18-CL 3 μm 120 Å phase. A gradient elution with buffer A (2% ACN and 0.1% FA) and buffer B (98% ACN and 0.1% FA) were performed. The following linear gradient was used: 0–0.1 min, 5–7% B; 0.1–60 min, 7–22% B; 60–70 min, 22–30% B; 70–74.9 min, 30–50% B; 74.9–75 min, 80% B; 75–80 min, 80% B; 80–80.5 min, 80 to 5% B; and 80.5–90 min, 5% B. MS analysis was obtained on the hybrid LCMS/MS system (Applied Biosystems API QSTAR, MA) at a mass spectra range of 350–1,250 Da for MS and 100–1,500 Da for MS/MS (Hsieh et al., 2009).

#### Proteomic Data Analysis and Bioinformatics

In reference to exiting literature (Zhang et al., 2016), ABI ProteinPilot software 4.5 (AB SCIEX) was used to identify and quantify and the iTRAQ-based proteomics data was abtained and analzed by Analyst QS 1.1 software (Applied Biosystems). Ratios with *p*-values < 0.05 and fold changes >1.5 were considered significant. Differentially expressed proteins (DEPs) were screened according to the criteria: *p*-values < 0.05 and fold changes >1.5. GO enrichment and KEGG pathway enrichment analyses of these DEPs were performed by DAVID databases (http://david.abcc.ncifcrf.gov).

### Integrated Analysis

To characterize the mechanism of AMR and its fractions in more detail, integrated core targets, compounds, and pathway analyses were conducted by the KEGG database and MetaboAnalyst (http://www.metaboanalyst.ca) based on data of the network pharmacology, metabolomic and proteomics. The “compound-target-metabolite-pathway” network was established by Cytoscape 3.7.1 software, which was used to clarify the molecular basis and the mechanism of AMR.

### Statistical Analysis

Measurement data were expressed as means ± SD. The data were analyzed by one-way analysis of variance with the statistical software SPSS 20.0 (Chicago, IL, United States). And *p* < 0.05 was considered statistical significance.

## Results

### Compound-Candidate Target Network Analysis of AMR

**FIGURE 1:**
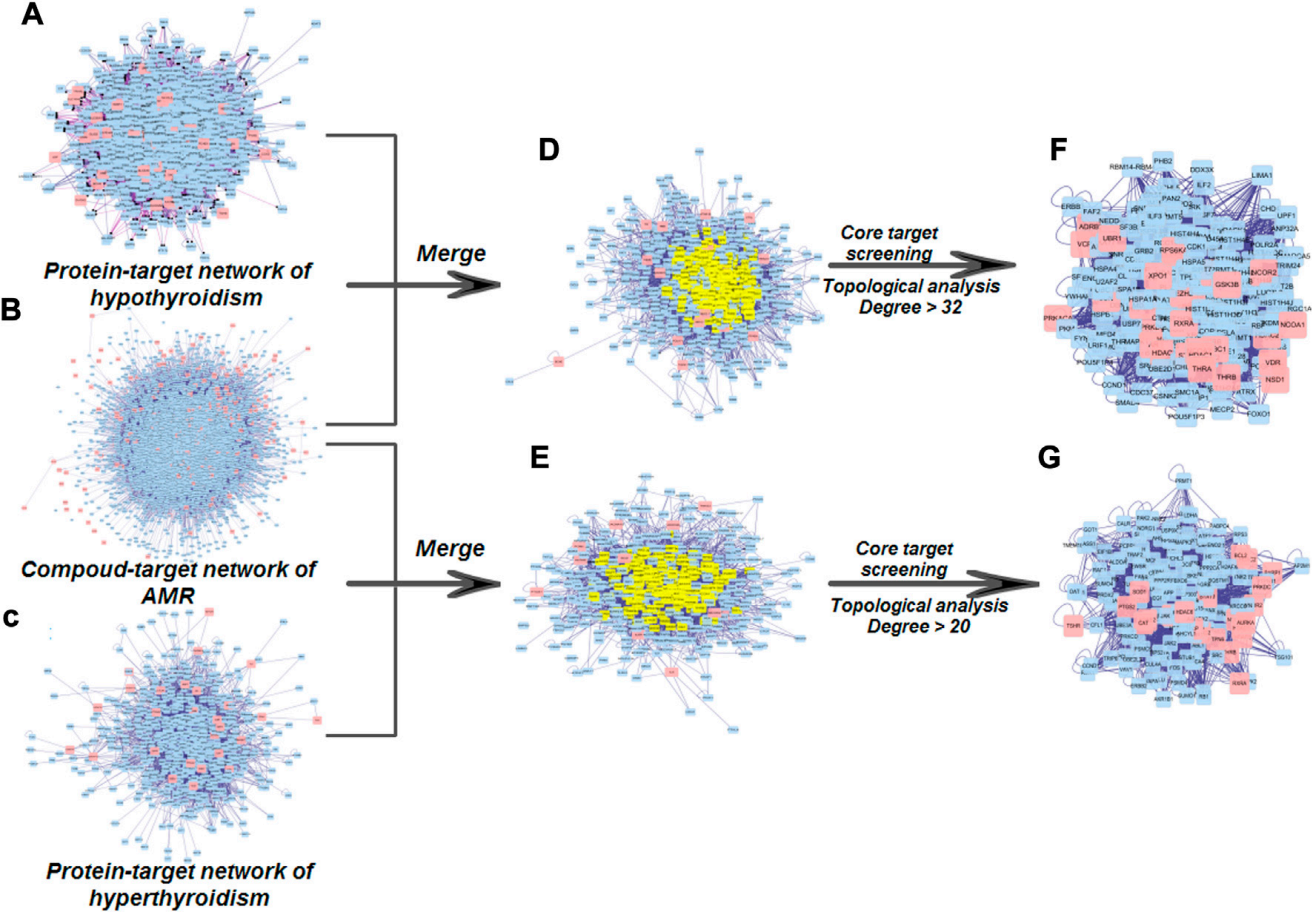
Core target screening processes used to establish the PPI regulation network **(A)** PPI network of hypothyroidism targets. **(B)** PPI network of targets of constituents from AMR **(C)** PPI network of hyperthyroidism targets. **(D)** PPI network of AMR against hypothyroidism **(E)** PPI network of AMR against hyperthyroidism. **(F)** PPI network of core targets of AMR against hypothyroidism. **(G)** PPI network of core targets of AMR against hyperthyroidism.

The water decoction of AMR was split into five fractions: VOF, LAF, ATF, OSF, and CPF. A total of 18 compounds were identified by HPLC in VOF, LAF, ATF, and OSF (Supplementary Table S1 and Supplementary Figure S1), indicating that the main components of VOF, LAF, ATF, and OSF were monoterpenoids and sesquiterpenoids, atractylenolide, polyacetylene, 5-hydroxymethyl furfural, and small molecular sugar. Furthermore, 461 potential targets of 18 compounds were identified from the Swiss Target Prediction and TCMSP databases (Supplementary Table S2). A total of 65 hyperthyroidism targets and 209 hypothyroidism targets were obtained from the TCMIP, CTD, and TTD databases (Supplementary Table S3). To identify the key targets of AMR effects on hyperthyroidism and hypothyroidism, we constructed the PPI based on Cytoscape 3.7.1 with the criteria “mean degree value > 32” and “mean degree value > 20” in the node size mapping (Figure 1); 224 and 170 core targets of AMR against hypothyroidism and hyperthyroidism, respectively, were obtained. Specifically, a total of nine compounds (attractylone in VOF; juniper camphor, atractylenolide III, stigmasterol, atractylenolide I, sitosterol, atractylenolide II and DBP in LAF; hexanolactam in ATF) have direct interaction with twenty-eight targets (such as: ESR1, AR, RXRA, CDK2, RPS6KA5, and so on) in hypothyroidism; a total of nine compounds (attractylone in VOF; juniper camphor, atractylenolide III, stigmasterol, atractylenolide I, sitosterol, atractylenolide II and DBP in LAF; hexanolactam in ATF) have direct interaction with twenty-two targets (such as: ADRB2, RXRA, PTGS2, ESR1, and so on) in hyperthyroidism (Supplementary Table S4 **)**. These results suggested that AMR exhibited multi-compound and multi-target feature for treating hypothyroidism and hyperthyroidism. And the strongest effect was observed with LAF, the weakest with OSF. To characterize the anti-hyperthyroidism and anti-hypothyroidism function of AMR, enrichment analysis of the pathway of candidate targets were performed by DAVID database. A total of 73 pathways of the anti-hypothyroidism function of AMR were significantly enriched and mainly involved in thyroid hormone signaling pathway, MAPK signaling pathway, neurotrophin signaling pathway, insulin resistance, insulin signaling pathway, adipocytokine signaling pathway, and cAMP signaling pathway (Supplementary Table S5). A total of 70 pathways of the anti-hyperthyroidism function of AMR were significantly enriched and mainly involved in the thyroid hormone signaling pathway, glycolysis/gluconeogenesis, biosynthesis of amino acids, and carbon metabolism (Supplementary Table S6). Metabolic processes of both anti-hyperthyroidism and anti-hypothyroidism function of AMR, including thyroid hormone signaling pathway, insulin resistance, insulin signaling pathway, adipocytokine signaling pathway, glycolysis/gluconeogenesis, biosynthesis of amino acids, and carbon metabolism, were closely related to the body’s energy and substance metabolism. These findings provided insight into the mechanism of AMR effects on hypothyroidism and hyperthyroidism.

### Index Measurement

Rectal temperature was significantly decreased (*p* < 0.05) in hypothyroidism rats, but increased (*p* < 0.05) in hyperthyroidism rats. Compared with the hypo-MO group in hypothyroidism, the rectal temperature in the hypo-ATF group was markedly increased. Body weight was up-regulated in hypothyroidism rats, but down-regulated (*p* < 0.05) in hyperthyroidism rats **(** Supplementary Figure S2). Body weight was increased and rectal temperature was decreased in hypothyroidism rats, but hyperthyroidism rats showed the opposite pattern. These results were consistent with the results of previous studies demonstrating that thyroid hormone status were correlated with body weight and energy expenditure (Fox et al., 2008; Iwen and Brabant, 2013; Knudsen et al., 2005).

Thyroid hormone regulates metabolic processes essential for normal growth and development as well as adult metabolism (Cheng et al., 2010; Mariash and Oppenheimer, 1980; Oetting and Yen, 2007). Numerous studies have shown that hypothyroidism patients often exhibited low basal metabolisms along with a decrease in T_3_ and T_4_ in plasma and increase in TSH; in contrast, hyperthyroidism patients showed a decrease in TSH and a increase in their basal metabolisms and T_3_ and T_4_ in plasma (Kong et al., 2015; Sawin et al., 1994). In our study, levels of T_3_ and T_4_ in serum were significantly down-regulated and level of TSH in serum was significantly up-regulated (*p* < 0.05) in hypothyroidism rats; and levels of T_3_ and T_4_ in serum were significantly up-regulated and level of TSH in serum was significantly down-regulated (*p* < 0.05) in hyperthyroidism rats (Supplementary Figure S2), demonstrating that the hypothyroidism and hyperthyroidism models were successfully established. Compared with the hypothyroidism group, the level of T_3_ was significantly increased in hypo-WD, hypo-VOF, hypo-CPF, hypo-LAF, hypo-OSF, and hypo-ATF (*p* < 0.05); the level of T_4_ was significantly up-regulated in hypo-WD, hypo-VOF, hypo-CPF, hypo-LAF, and hypo-ATF (*p* < 0.05); the level of TSH was significantly down-regulated in hypo-WD, hypo-CPF and hypo-LAF (*p* < 0.05). Compared with the hyperthyroidism group, the level of T_3_ was significantly increased in hype-VOF, hype-CPF, hype-LAF, and hype-OSF (*p* < 0.05) **(** Supplementary Figure S2). Thus, AMR and its fractions promoted the recovery of hypothyroidism rats by regulating the content of T_3,_ T_4_ and TSH, but did not have any notable effect on hyperthyroidism rats.

The Na^+^-K^+^-ATPase extrudes three Na^+^ ions in exchange for two K^+^ ions and harvests energy from the hydrolysis of an ATP molecule (Einholm et al., 2016; Wang X. et al., 2015). Furthermore, researches showed that Na^+^-K^+^-ATPase is also closely linked to the amino acids and glucose transport (Crunkhorn and Patti, 2008). Several studies have shown that Na^+^-K^+^-ATPase activity is an energy dependent process and energy consumption increases as Na^+^-K^+^-ATPase activity increases (Emadi et al., 2019). In this study, Na^+^-K^+^-ATPase activity was significantly down-regulated in hypothyroidism rats but up-regulated in hyperthyroidism rats **(** Supplementary Figure S2 **)**, indicating that energy expenditures were low in hypothyroidism rats and high in hyperthyroidism rats. Furthermore, hypo-WD, hypo-VOF, hypo-CPF, and hypo-LAF promoted the recovery of hypothyroidism rats, showing that AMR and its components could up-regulate the lower energy metabolism of hypothyroidism rats.

### Proteomics

PROTEIN PILOT 4.5 software was used to match proteins with those in the Uniprot database; overall, 1,499 proteins in the hypothyroidism model were identified with a 95% confidence level. The relative protein expression values were compared between groups (CON *vs*. hypo-MO group; hypo-WD and its fractions *vs*. hypo-MO group) to identify DEPs (*p* < 0.05 and changes >1.5-fold) that played potentially important roles in disease progression, that could potentially be used to diagnose diseases and that could reveal molecular drug targets. A total of 78 DEPs in CON *vs*. hypo-MO groups and 34, 42, 34, 24, 39, and 38 DEPs in hypo-WD, hypo-VOF, hypo-CPF, hypo-LAF, hypo-OSF, hypo-ATF, *vs*. hypo-MO groups were screened in the hypothyroidism model and the results were shown in Supplementary Tables S7–13. These DEPs including Pygl, Pklr, Pc, Ehhadh, Acox1, Fasn, Acly, Acsl1, Acsl5, Pgm1, Suclg1, Gpt, Idh1, Fh, and Adh1 maight be as target proteins of AMR and its fractions for hypothyroidism.

**FIGURE 2:**
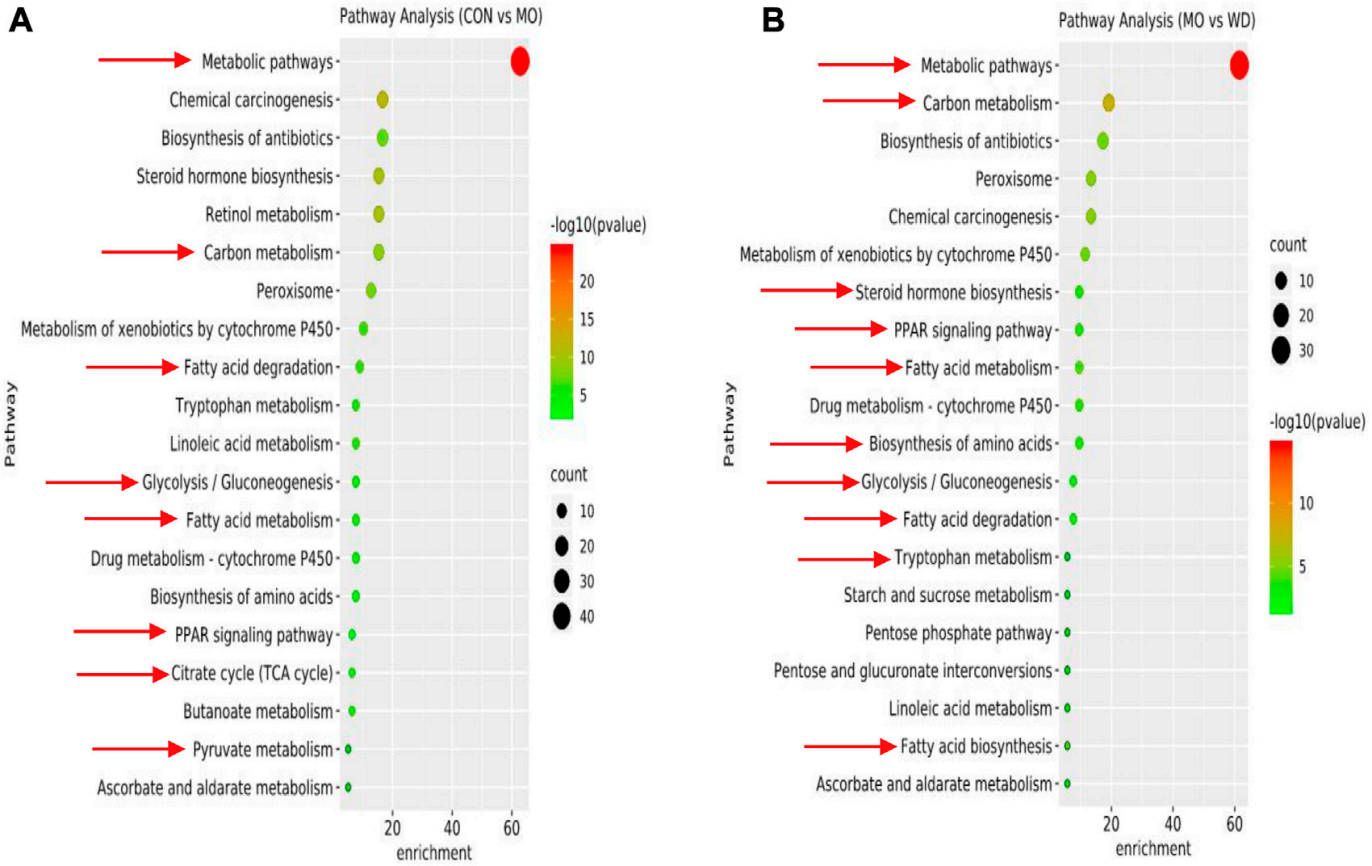
DEPs of KEGG enrichment in hypothyroidism model **(A)** DEPs of CON vs MO. **(B)** DEPs of MO vs WD.

GO enrichment analysis, including biological processes, cellular component, and molecular function, were performed using DAVID database (Supplementary Figures S4–10). The biological processes were primarily involved in oxidation-reduction process, metabolic process, fatty acid biosynthetic process, and fatty acid beta-oxidation. The results of KEGG enrichment were shown in Figure 2 and Supplementary Figure S11. We found that DEPs of KEGG enrichment in the hypothyroidism model were primarily involved in metabolic pathways, carbon metabolism, fatty acid degradation, glycolysis/gluconeogenesis, fatty acid metabolism, PPAR signaling pathway, TCA cycle, and pyruvate metabolism. These pathways were closely related to substance and energy metabolism. In addition, the DEPs of AMR and its fractions were also primarily involved in these pathways. These results suggested that AMR and its fractions played roles in regulating the substance and energy metabolism for the treatment of hypothyroidism.

### Metabolomics

In the OPLS-DA score plots, a clear separation was observed between CON and hypo-MO (Supplementary Figures S12), hypo-MO and hypo-AMR, and its fractions (Supplementary Figures S13A–L), CON and hype-MO (Supplementary Figures S14), hype-MO and hype-AMR, and its fractions (Supplementary Figures S15 A–L). The R2Y and Q2 (cum) of the newly established OPLS-DA model were 0.995 and 0.786 in positive ion mode (hypothyroidism), 0.995 and 0.654 in positive ion mode (hyperthyroidism), 0.994 and 0.623 in negative ion mode (hypothyroidism), and 0.999 and 0.914 in negative ion mode (hyperthyroidism), respectively, indicating that the fitness and predictions of the model were robust.

OPLS-DA (A1, B1) score plots of hypothyroidism rats urine metabolites were obtained from the normal group (n = 7) and model group (n = 7), and permutation tests (A2, B2) for OPLS-DA models were conducted in the positive ion mode (A) and negative ion mode (B). The OPLS-DA score plots revealed a distinct separation between the normal and model group. OPLS-DA models were validated by conducting 100 random permutations and examining the correlation between the *x*-axis, representing the correlation coefficient between the original y variable and the permuted y variable, and the *y*-axis, representing the correlation coefficient between the values of R2 and Q2.

**FIGURE 3:**
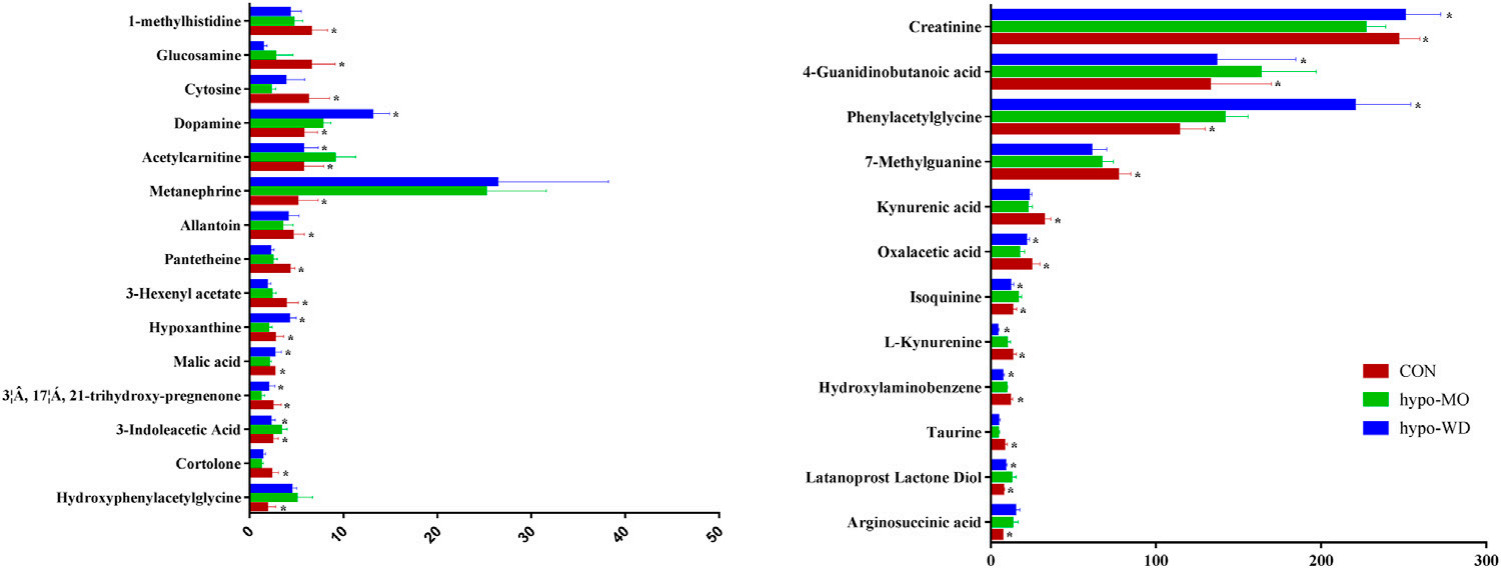
Bar graph of 36 representative metabolites in urine with a reversing trend to normal induced by WD treatment. The *x*-axis indicates the relative peak intensities. Data were expressed as mean ± SD (n = 7/group). *p < 0.05 vs. the model group.

In this study, twenty-seven (seventeen down-regulated and ten up-regulated) differential metabolites (Figure 3, Supplementary Table S14) were identified in hypothyroidism rats, which were associated with alanine, aspartate, and glutamate metabolism; tyrosine metabolism, tryptophan metabolism; arginine and proline metabolism; TCA cycle; steroid synthesis biosynthesis; purine metabolism; and energy metabolism. Levels of oxalacetic acid, malic acid, glucosamine, 3-hexenyl acetate, cortolone, 3β, 17α, 21-trihydroxy-pregnenone, kynurenic acid, l-lynurenine, 1-methylhistidine, pantetheine, creatinine, taurine, hypoxanthine, 7-methylguanine, allantoin, hydroxylaminobenzene, and cytosine were significantly down-regulated and levels of hydroxypropanal, acetylcarnitine, 3-Indoleacetic acid, arginosuccinic acid, phenylacetylglycine, metanephrine, hydroxyphenylacetylglycine, isoquinine, latanoprost lactone diol, and dopamine were significantly up-regulated in hypothyroidism rats. The related pathways were identified with the MetaboAnalyst tool (Supplementary Figures S16). Furthermore, oxalacetic acid, malic acid, creatinine, 3β, 17α, 21-trihydroxy-pregnenone, acelycarnitine, hypoxanthine, 3-hydroxypropanal, isoquinoline, and 3-indoleacetic in the hypo-WD group; oxalacetic acid, creatinine, hydroxypropanal, and latanoprost lactone diol in the hypo-CPF group; 3β, 17α, 21-trihydroxy-pregnenone, creatinine, and isoquinine in the hypo-VOF group; oxalacetic acid, isoquinoline, propylparaben, and latanoprost lactone diol in the hypo-LAF group; glucosamine, acetylcarnitine, 3-indoleacetic acid, 1-methylhistidine, creatinine, isoquinine, and latanoprost lactone diol in the hypo-OSF group; and oxalacetic acid, 3-indoleacetic, and isoquinoline in the hypo-ATF group were significantly reversed, indicating that AMR and its fractions could significantly reverse hypothyroidism in rats and that the action of WD was most significant.

**FIGURE 4:**
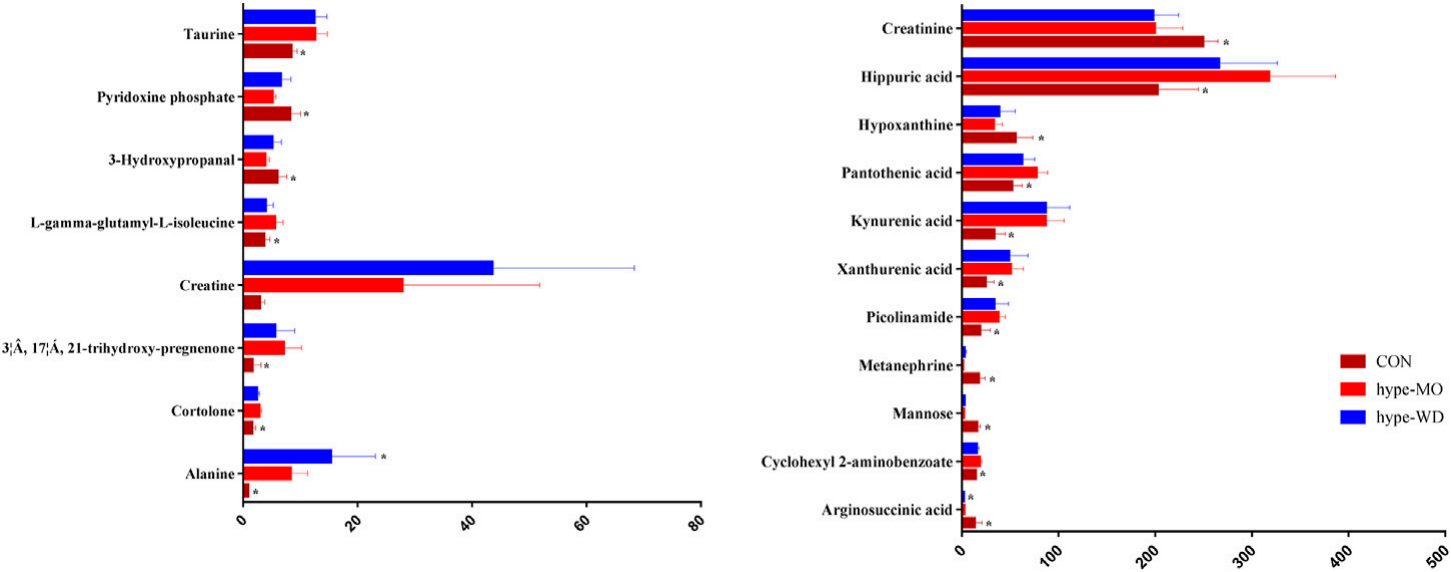
Bar graph of 26 representative metabolites in urine with a reversing trend to normal induced by WD treatment. The *x*-axis indicates the relative peak intensities. Data were expressed as mean ± SD (n = 7/group). *p < 0.05 vs. the model group.

Nineteen (thirteen up-regulated and six down-regulated) differential metabolites were identified in hyperthyroidism rats (Figure 4, Supplementary Table S15); these metabolites were primarily involved in taurine and hypotaurine metabolism, pantothenate and CoA biosynthesis, TCA cycle, and vitamin B6 metabolism. Mannose, xanthurenic acid, kynurenic acid, alanine, hippuric acid, taurine, pantothenic acid, creatine, picolinamide, cyclohexyl 2-aminobenzoate, cortolone, 3β, 17α, 21-trihydroxy-pregnenolone, and L-gamma-glutamyl-l-isoleucine were significantly up-regulated, and pyridoxine phosphate, creatinine, hypoxanthine, 3-hydroxypropanal, arginosuccinic acid, and metanephrine were significantly down-regulated in hyperthyroidism rats. After receiving treatment with AMR and its fractions, hippuric acid, pantothenic acid, cyclohexyl 2-aminobenzoate, and metanephrine in the hype-CPF group; pantothenic acid, cyclohexyl 2-aminobenzoate, and 3-hydroxypropanal in the hype-VOF group; hippuric acid, pantothenic acid, picolinamide, cyclohexyl 2-aminobenzoate, and 3-hydroxypropanal in the hype-LAF group; hippuric acid, 3-hydroxypropanal, and metanephrine in the OSF group; hypoxanthine, picolinamide, metanephrine, and L-gamma-glutamyl-l-isoleucine in the hype-ATF group were reversed.

**FIGURE 5:**
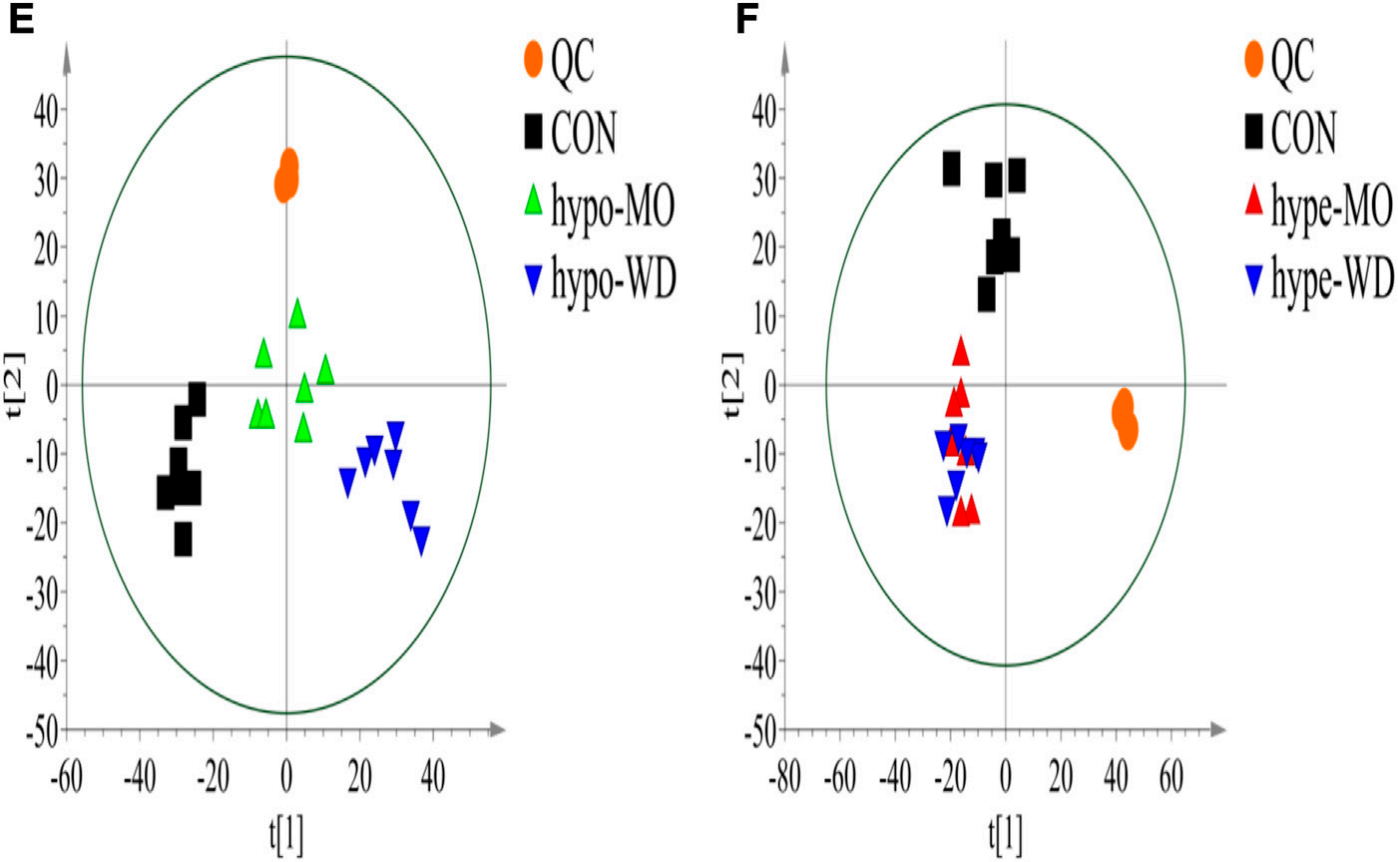
PLS-DA scores plot of hypothyroidism (E) and hyperthyroidism (F) rat urine metabolites from the CON group (n = 7), MO group (n = 7) and WD group.

Furthermore, a PLS-DA model was built **(** Figure 5 and Supplementary Figures S17 **)**. The QC sample features were tightly clustered in the score plot of PLS-DA, ingdicating that the proposed system had good stability for this metabolomics study. The model evaluation parameters (R2Y = 0.991 cum, Q2 = 0.897cum) were obtained, which indicated that the model was stable and reliable. Separation of the CON and hypo-MO groups, CON and hype-MO were clearly observed in both hypothyroidism and hyperthyroidism rats (Figure 5). Hypo-MO and hypo-WD groups were separated in hypothyroidism rats, and Hype-MO and hype-WD groups were overlapped in hyperthyroidism rats, indicating that AMR provided different degrees of protection for hypothyroidism but did not have an effect on hyperthyroidism (Figure 5). Similarly, different degrees of protective effects conferred to hypothyroidism rats were detected in fractions of AMR **(** Supplementary Figures S18A–J and Supplementary Figure S19A–J **)**.

### Integrating Analysis of Omics and Network Pharmacology

**FIGURE 6:**
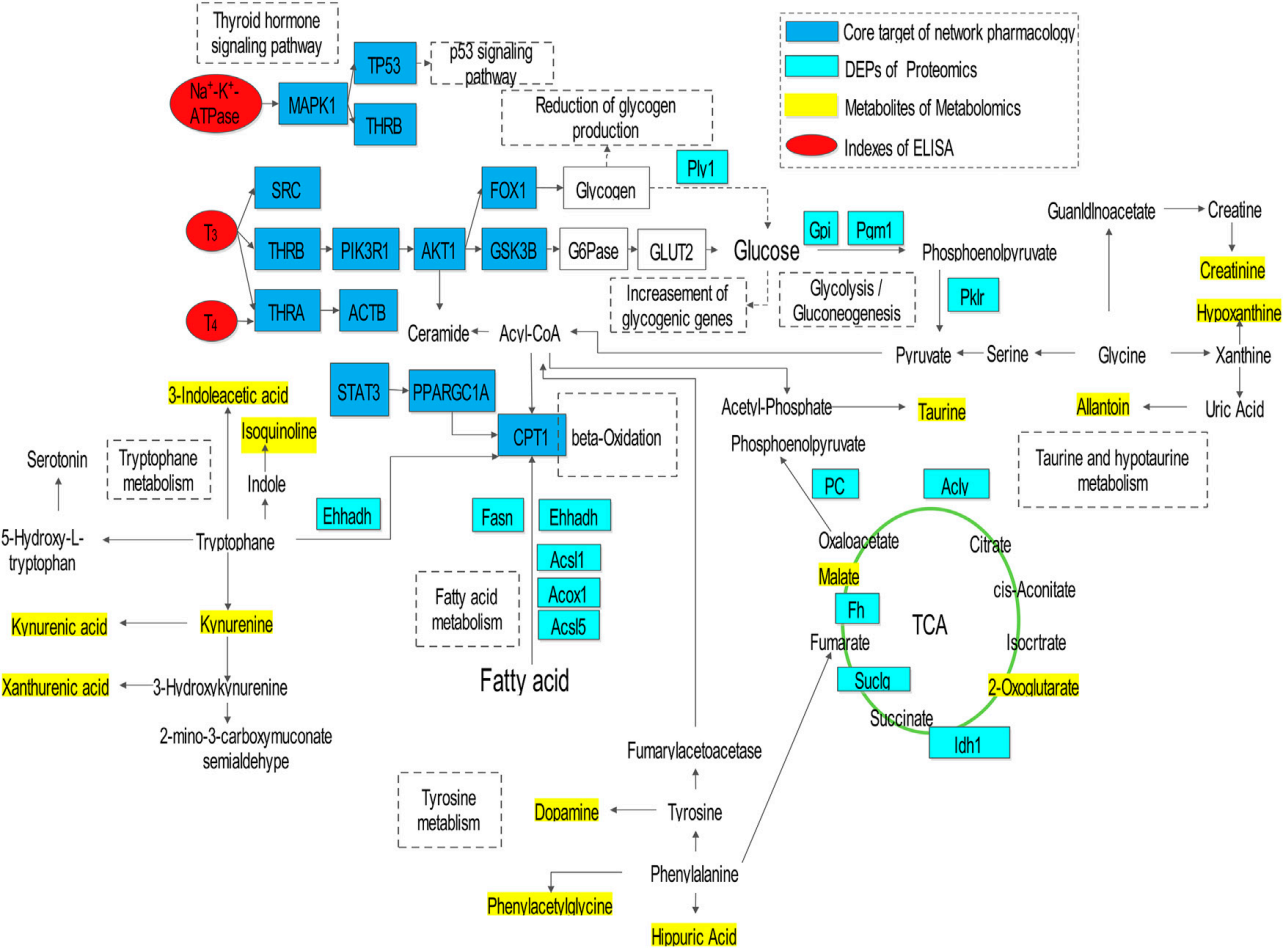
Potential metabolic pathways regulated in hypothyroidism rats after treatment with AMR.

The results of integrating analyses of omics and network pharmacology were shown in (Figure 6). AMR and its split components ameliorated the hypothyroidism model through regulation of core targets (MAPK1, TP53, THRB, THRA, AKT1, STAT3, PPARGC1A, CPT1, and GSK3B), metabolites (2-oxoglutarate, 3-indoleacetic acid, isoquinoline, kynurenine, kynurenic acid, xanthurenic acid, taurine, allantion, creatinine, hypoxanthine, dopamine, phenylacetylglycine, hippuric acid, and malate), and DEPs (Plyl, Cpt1, Ehhadh, Fasn, Acsl1, Acox1, Acsl5, Pklr, Gpi, Pgml, Pc, Acly, Fh, Idh1, and Suclg), followed by adjustment of substance and energy metabolism, which involved in fatty acid metabolism, glycolysis/gluconeogenesis, citrate cycle (TCA cycle), tryptophan metabolism, thyroid hormone signaling pathway, p53 signaling pathway, taurine and hypotaurine metabolism, and tyrosine metabolism.

**FIGURE 7:**
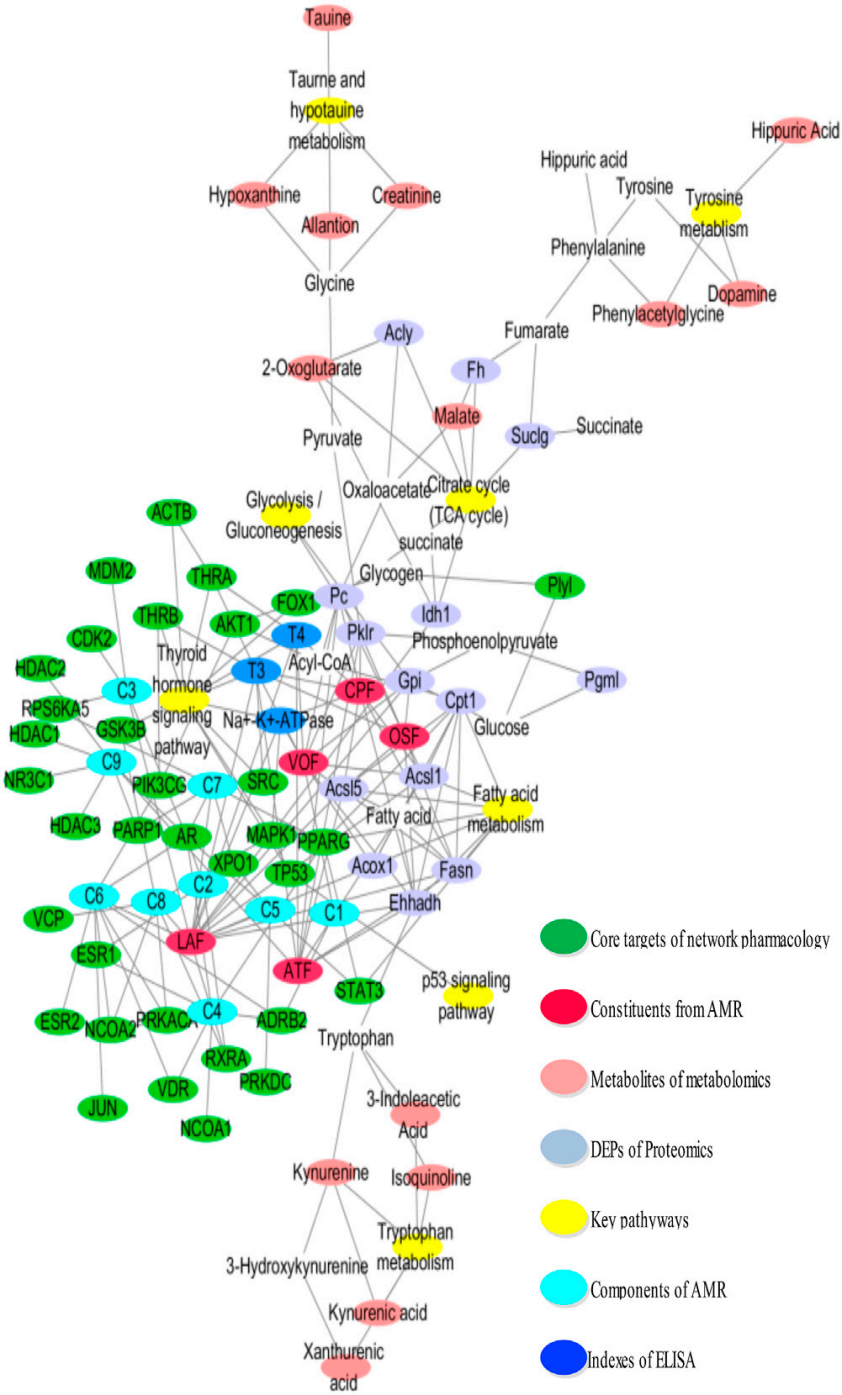
The network is graphically represented with components, constituents, core targets, metabolites, DEPs, and pathways as nodes, their relations as edges.

Moreover, the “compound-target-metabolite-pathway” network shown in Figure 7 was established by Cytoscape 3.7.1 software. In the network, VOF connected one compound (C1, attractylone) and one target (ADRB2); LAF connected seven compounds (C2, juniper camphor; C3, atractylenolide III; C4, stigmasterol; C5, atractylenolide I; C6, sitosterol; C7, atractylenolide II; C8, DBP) and 22 targets (RXRA, AR, ESR1, RPS6KA5, MDM2, CDK2, ADRB2, PRKACA, NCOA1, NCOA2, VDR, PPARG, STAT3, PRKDC, XPO1, PIK3CG, JUN, ESR2, PARP1, GSK3B, MAPK1, and VCP); and ATF connected one compound (C9, hexanolactam) and six targets (HDAC3, HDAC2, HDAC1, NR3C1, PARP1, AR). Moreover, these connections showed that the lactones fraction, primarily containing atractylenolide, were the main component of AMR that ameliorated hypothyroidism. The omics analysis also revealed that the lactones fraction containing atractylenolide and the crude polysaccharides fraction containing inulin-type polysaccharides were the main components of AMR that ameliorated hypothyroidism.

## Discussion

According to TCM theory, Chinese drugs with *warm* or *hot* natures are suitable for the treatment of *cold* syndromes, while drugs with *cool* or *cold* natures are suitable for the treatment of *heat* syndromes. Hypothyroidism and hyperthyroidism are respectively considered as *cold* and *heat* diseases. Previous experiments in our laboratory have shown that aconiti lateralis radix praeparaia, a *hot* drug, could ameliorate hypothyroidism rats with *cold* syndromes by regulating the metabolism of substances and energy (Xiao et al., 2017). AMR has often been prescribed to treat hypothyroidism, as well as hyperthyroidism; however, it has been used more commonly for the former rather than the latter. In this study, we used network pharmacology to show that AMR has a potential therapeutic effect on hypothyroidism and hyperthyroidism, but the effect was stronger for the former. Furthermore, omics analysis confirmed these results, as AMR and its split components ameliorated the effects of hypothyroidism to different degrees by promoting substance and energy metabolism, but had no significant contribution to ameliorating hyperthyroidism. In addition, AMR, a *warm* drug, had therapeutic effects on hypothyroidism (a *cold* syndrome) and not on hyperthyroidism (a *heat* syndrome), which was consistent with TCM theory.

Integrative network pharmacology, proteomics, and metabolomics analysis were performed to evaluate the holistic efficacy and molecular basis of AMR as well as the mechanism underlying how AMR ameliorated hypothyroidism. The key pathways of AMR involved in the amelioration of hypothyroidism and relating to substance and energy metabolism were screened. These pathways mainly involved in thyroid hormone signaling pathway, glycolysis/gluconeogenesis pathways, TCA cycle pathway, fatty acid metabolism pathway, amino acid pathway metabolism, and taurine and hypotaurine metabolism. These findings revealed that the lactones fraction, primarily containing atractylenolide, and the crude polysaccharides fraction, primarily containing inulin-type polysaccharides AMR, were considered the most important components of AMR contributing to the amelioration of hypothyroidism. Figures 6 and 7 show the interaction network, which illustrate how AMR exerts its ameliorating properties for hypothyroidism. These results suggested that AMR held the multi-compound to multi-target feature for hypothyroidism treatment. At the same time, we found nine compounds (attractylone, juniper camphor, atractylenolide III, stigmasterol, atractylenolide I, sitosterol, atractylenolide II, DBP, hexanolactam) might be the molecular basis of AMR for ameliorating hypothyroidism. These compounds acted directly or indirectly on relative targets (ADRB2, RXRA, AR, ESR1, RPS6KA5, MDM2, CDK2, ADRB2, PRKACA, NCOA1, NCOA2, VDR, PPARG, STAT3, PRKDC, XPO1, PIK3CG, JUN, ESR2, PARP1, GSK3B, MAPK1, VCP, HDAC3, HDAC2, HDAC1, NR3C1, PARP1, AR) and further treated hypothyroidism through the key relevant pathways such as thyroid hormone signaling pathway, glycolysis/gluconeogenesis pathways, TCA cycle pathway, fatty acid metabolism pathway and fatty acid metabolism pathway. Below, we provided a detailed discussion of key relevant pathways.

### Thyroid Hormone Signaling Pathway

Body weight was increased, and rectal temperature, levels of T_3_, T_4_ and TSH in serum, and Na^+^-K^+^-ATPase activity were down-regulated in hypothyroidism rats, which were consistent with the clinically observed symptoms of hypothyroidism, indicating that the hypothyroidism model (a *cold* syndrome) was successfully established. These indexes in hypothyroidism rats were significantly reversed in AMR and its fraction groups. And the hyperthyroidism model (a *heat* syndrome) was also successfully established. However, after treatment, level of T_3_ and T_4_ were up-regulated in hyperthyroidism rats, indicating AMR and its components had no therapeutic effects on hyperthyroidism. Furthermore, results of the proteomics and metabolomics indicated that AMR and its fractions could reverse the proteins or metabolites of the citrate cycle, tryptophan metabolism, fatty acid metabolism, and glycolysis/gluconeogenesis, ultimately promoting the energy metabolism, exerting a recovery effect on hypothyroidism rats. However, energy metabolism was increased in hyperthyroidism rats, but AMR and its fractions did not show any reverse action. In a word, following treatment with AMR and its fractions, T_3_, T_4_ and TSH levels should return to normal in hypothyroidism, but not in hyperthyroidism. Meanwhile, the results of network pharmacology indicated that THRA and THRB might be potential therapeutic targets for hypothyroidism. THRA1, THRB1 and THRB2 were three major thyroid hormone receptors and mediated thyroid hormone actions, which were encoded by THRA and THRB (Minakhina et al., 2020). THRA and THRB mutations directly caused abnormal thyroid hormone levels, which was associated with tissue-specific hypothyroidism (Schoenmakers, 2012). Hence, the active compouds of AMR have direct interaction with THRA and THRB targets, and up-regulated the T_3_ and T_4_ levels to alleviate hypothyroidism. It might be one mechanism by which AMR modulates thyroid hormone levels in hypothyroidism.

#### Glycolysis/Gluconeogenesis Pathways

Thyroid hormones play a significant role in glucose homeostasis (Xu et al., 2019). Thyroid hormone functions in the liver, white adipose tissue, skeletal muscle, and pancreas by controlling plasma glucose levels, insulin sensitivity, and carbohydrate metabolism. The reduced activity of mitochondria provides a link between a well-described action of thyroid hormone and a defect in Type 2 diabetes (Tashima et al., 2000). Glycogen is a form of sugar storage in animals. Liver and muscle are the main organs for storing glycogen. Glycogen in muscle is primarily used for muscle contraction, but the glycogen in liver is an important source of blood glucose. Glycogen phosphorylase (Pygl) is a key enzyme in glycogen metabolism (Tashima et al., 2000). In this study, the expression of Pygl was significantly decreased in hypothyroidism rats, which indicated that the source of blood glucose was down-regulated; the reverse effect was detected in the hypo-WD, hypo-VOF, and hypo-OSF groups. Previous studies have established that T_3_ stimulates gluconeogenesis, especially in the hyperthyroid state, and that hypothyroidism is associated with reduced gluconeogenesis (Comte et al., 1990). Treatment with T_4_ increases alanine transport into hepatocytes, increasing the production of metabolic intermediates of the gluconeogenic pathway and ultimately the conversion of alanine into glucose (Singh and Snyder, 1978). Evaluation of T_3_ treatment on target genes in the liver has revealed an increase in the genes regulating glycogenolysis and gluconeogenesis (Feng et al., 2013). Specifically, regulation of phosphoenolpyruvate carboxykinase, the rate-limiting step in gluconeogenesis, is critical for glucose homeostasis and is regulated by TR and CCAAT enhancer-binding protein in the liver (Park et al., 1999). In this study, expression of pyruvate carboxykinase (Pklr), pyruvate carboxylase (Pc), glycogen phosphorylase (Pyg1), and Phosphoglucomutase-1 (Pgm1) were significantly down-regulated in hypothyroidism rats, which indicated that the glycolysis pathway was blocked. Interestingly, expression of these proteins (AMR: Pklr, Pgm1, and Pc; VOF: Pklr, Pgm1, Pyg1, and Pc; CPF: Pklr; OSF: Pklr, Pgm1, Pyg1, and Pc; ATF: Pklr) were significantly up-regulated after treatment of AMR and its fractions. In addition, Pklr was the rate-limiting enzyme in glycolysis (Jia et al., 2019). The increase in the expression of Pklr verified the booster effects of AMR and its fractions on glycolytic flux. These findings indicated that promotion of glycolysis might be one of the mechanisms in AMR treatment for hypothyroidism.

#### TCA Cycle Pathway

*Cis*-aconitate, succinic acid, citric acid, and a-ketoglutaric acid as important intermediates of the tricarboxylic acid cycle (TCA), were associated with glucose degradation and energy metabolism of body. In this study, the results of metabolomics and proteomics indicated that the levels of metabolites (citric acid, a-ketoglutaric acid), expression of Acly (ATP-citrate synthase) were significantly decreased in hypothyroidism rats, indicating that the suppression of tricarboxylic acid cycle (TCA) activity caused disordered energy metabolism. Moreover, levels of *cis*-aconitate and a-ketoglutaric acid were significantly up-regulated, suggesting that energy metabolism increased significantly in hyperthyroidism rats. There was evidence to suggest that thyroid hormones significantly influenced TCA cycle by increasing the mitochondrial Ca^2+^ and the substrate supply, stimulating the TCA cycle activity (Briere et al., 2006). Some studies have demonstrated that biogenesis and respiratory capacity of free mitochondria and neuronal oxygen consumption in the cerebral cortex were down-regulated in hypothyroidism rats (Bienvenida et al., 2009). In our study, energy metabolism dysfunction was detected in hypothyroidism rats, which were in line with the results of earlier research on lower energy metabolism in hypothyroidism (Lu et al., 2011; Mcgarry and Brown, 2010; Obregon, 2008). Furthermore, citric acid, a-ketoglutaric acid, and acly were significantly up-regulated in hypo-WD and a-ketoglutaric acid in hypo-CPF, hypo-LAF, and hypo-ATF groups; acly in the hypo-CPF, hypo-OSF, and hypo-ATF groups were significantly increased compared with the hypothyroidism group, suggesting that AMR and its fractions might stimulate the TCA cycle to induce the energy metabolism of hypothyroidism rats. Additionally, we found a increased activity of the tricarboxylic acid cycle in hyperthyroidism rats, but there was no improvement in AMR and its fractions groups.

#### Fatty Acid Metabolism Pathway

Evidences demonstrated that lipolysis and lipogenesis were regulted by thyroid hormone (Lu et al., 2011). Thyroid hormone have an important role of the regulation of the conversion of preadipocytes to adipocytes (Obregon, 2008). Malonyl-CoA was used by fatty acid synthase to synthesize palmitic acid but was also serving to allosterically inhibit carnitine palmitoyl transferase (CPT1), the protein responsible for transporting long-chain fatty acids into mitochondria for β-oxidation (Mcgarry and Brown, 2010). Peroxisomal bifunctional enzyme (Ehhadh) was one of the dehydrogenases and played an important role in fatty acid β-oxidation (Hashimoto, 1999; Hiroshi et al., 2014). Acox1 and Acox3 are the rate-limiting enzymes in fatty acid β-oxidation. Fatty acid synthase (Fasn) was a key enzyme in fatty acid synthesis. In our study, the expression of Fasn, Acsl1, Acsl5, Ehhadh, and Acox1 were significantly down-regulated in hypothyroidism rats, indicating that the synthesis of fatty acid and the oxidation process of fatty acids were inhibited. Furthermore, the level of Fasn, Ehhadh, Acsl1, Acsl5, and Acox1 in the hypo-WD group; Fasn, Ehhadh, and Acox1 in the hypo-VOF group; Fasn, Ehhadh, and Acsl1 in the hypo-CPF group; Fasn, Ehhadh, Acsl1, Acsl5, and Acox1 in the hypo-LAF group; Fasn, Ehhadh, Acsl1, and Acsl5 in the OSF group; and Fasn, Ehhadh, Acsl1, and Acsl5 in the hypo-ATF group were significantly up-regulated, showing that AMR and its fractions could stimulate fatty acid metabolism. These results indicated that Fasn, Acsl1, Acsl5, Ehhadh, and Acox1 were potential therapeutic targets for AMR and its fractions in treatment of hypothyroidism. Meanwhile, fatty acid β-oxidation was one of the major pathways of energy metabolism (Castagnaro et al., 2016). Therefore, AMR and its fractions could promote fatty acid metabolism by these DEPs, so as to promote energy metabolism, which might be one of the mechanisms in AMR treatment for hypothyroidism.

#### Amino Acid Pathway Metabolism

Tryptophan as an essential amino acid, has a variety of physiological function. Hypothyroidism patients often present with experience anorexia and poor appetite, and dietary tryptophan deficiency can alter thyroid hormone levels (Carew et al., 1983). In addition, the expression of Ehhadh, Haao, and Cyp1A2 were up-regulated and Cat, Aldh2, and Ehhadh were down-regulated in hypothyroidism rats. The metabolic products of tryptophan, such as kynurenic acid and 3-hydroxyhippuric acid, were up-regulated in hyperthyroidism rats. In contrast, levels of kynurenic acid, kynurenine acid, and xanthurenic acid were down-regulated, and 3-indoleacetic acid and isoquinoline up-regulated, in hypothyroidism rats. Thus, tryptophan metabolism pathway was abnormal in hypothyroidism and hyperthyroidism rats, which was consistent with the results of previous studies that have documented tryptophan deficits, can lead to thyroid dysfunction (Kulikov and Jeanningro, 2000; Mannisto and Ranta, 1978). After treatment with AMR and its fractions, the high expression of Hadha, Haao, Cyp1a2, the high level of 3-indoleacetic acid and isoquinoline, the low expression of cat, aldh2, Ehhadh, and xanthurenic acid were significantly reversed in hypothyroidism rats, indicating that AMR and its splitted fractions could ameliorate abnormal tryptophan metabolism. However, in the present study, no significant changes in kynurenic acid and 3-hydroxyhippuric acid levels following AMR and its fractions treatment were observed in hyperthyroidism. These results further verified that AMR were able to ameliorate the hypothyroidism instead of hyperthyroidism.

#### Taurine and Hypotaurine Metabolism

Creatine plays an essential role in energy storage and transmission in several tissues (Arias et al., 2006). In our study, level of creatinine was down-regulated in hypothyroidism rats and up-regulated in hypothyroidism rats. Meanwhile, level of creatinine was up-regulated in hypo-WD, hypo-CPF, and hypo-OSF groups compared with hypo-MO group. These findings indicated that AMR and its fractions could modify the lower energy storage and transmission in hypothyroidism by up-regulating the level of creatine.

#### Phenylalanine Metabolism

The levels of phenylacetylglycine, dopamine, phenylethylamine and hippuric acid have significantly change in hypothyroidism model, which indicates that hypothyroidism model is closely related to phenylalanine metabolism disorder. As potential biomarkers, phenylalanine generally is first converted into tyrosine by phenylalanine hydroxylase and subsequently into dopamine, catecholamines, norepinephrine and epinephrine (Fernstrom and Fernstrom, 2007). Some studies have demonstrated that the level of phenylalanine and tyrosine are strongly associated with thyroid function (Sjoberg et al., 1998), and neonatal hypothyroidism induces striatal dopaminergic dysfunction (Vaccari et al., 1990). In our study, the levels of phenylalanine and tyrosine were significantly increased, which indicated that phenylalanine metabolism was disordered in hypothyroidism model. These results further confirmed hypothyroidism may be involved in phenylalanine metabolism. After treatment of AMR and its fractions, the levels of phenylethylamine and hippuric acid didn’t reverse in hypothyroidism. This observation implied that AMR didn’t exert therapeutic effects via the paythway of phenylalanine metabolism.

To sum up, the effects of the AMR on hypothyroidism and hyperthyroidism in animal models were investigated. Body weight, body temperature, the levels of T_3_, T_4_ and TSH in serum and the activity of Na^+^-K^+^-ATPase were used to evaluate the models. In hypothyroidism model, body weight and the level of TSH in serum of the model rats increased and the body temperature, levels of T_3_ and T_4_ in serum and the activity of Na^+^-K^+^-ATPase of the model rats decreased significantly, indicating successful establishment of the hypothyroidism model. After AMR and its fractions treatment, these indexes were ameliorated at different degrees, indicating AMR and its fractions could achieve a better therapeutic effect for hypothyroidism, and LAF and CPF had more significant effects. In hyperthyroidism model, body weight and level of TSH in serum of the model rats decreased and body temperature, levels of T_3_ and T_4_ in serum and the activity of Na^+^-K^+^-ATPase of the model rats increased significantly, which indicated successful establishment of the model of hyperthyroidism. These indexes were not improved by AMR and its fractions treatment, however, the levels of T_3_ in serum were further increased after VOF, CPF, LAF and OSF treatment, indicating that AMR and its fractions demonstrated no significant effects on hyperthyroidism. These findings verified that AMR was able to ameliorate the hypothyroidism instead of hyperthyroidism.

Furthermore, proteomics and metabolomics analysis indicated that AMR and its fractions could promote glycolysis, TCA cycle, fatty acid metabolism and amino acid metabolism, and then promoted the substance and energy metabolism, which might be an important mechanism underlying therapeutic effetcs of AMR in the treatment of hypothyroidism. As shown in the results of proteomics and metabolomics analysis, AMR were able to ameliorate the hypothyroidism instead of hyperthyroidism.

In addition, we adopted network pharmacology to further explore the mechanisms of AMR on hypothyroidism. And the results indicated that nine compounds (attractylone, juniper camphor, atractylenolide III, stigmasterol, atractylenolide I, sitosterol, atractylenolide II, DBP, hexanolactam) might be molecular basis of AMR for ameliorating hypothyroidism. The mechanism was mainly related to thyroid hormone signaling pathway, glycolysis/gluconeogenesis pathways, TCA cycle pathway, fatty acid metabolism pathway and fatty acid metabolism pathway. In comparison, LAF hold the strongest effect as well as OSF hold the weakest effect. These results were consistent with proteomic and metabolomics analysis results. Comprehensive analysis results indicated that as the essential substance basis of AMR, LAF and CPF were able to ameliorate the hypothyroidism model to different degrees, whereas no significant improvements were noted in the hyperthyroidism model.

## Conclusion

In sum, AMR was able to ameliorate the hypothyroidism instead of Hyperthyroidism. The mechanism of AMR in the treatment of hypothyroidism were mainly related to increasing thyroid hormone levels and promoting glycolysis, TCA cycle, fatty acid metabolism and amino acid metabolism, and then promoting the substance and energy metabolism. And nine compounds (attractylone, juniper camphor, atractylenolide III, stigmasterol, atractylenolide I, sitosterol, atractylenolide II, DBP, hexanolactam) might be molecular basis of AMR for ameliorating hypothyroidism. The lactones fraction, primarily containing atractylenolide, and the crude polysaccharides fraction, primarily containing inulin-type polysaccharides, were considered the most important components of AMR. This study provided novel insight into how network pharmacology, proteomics, and metabolomics could be used as effective tools for elucidating the efficacy and molecular basis of and the mechanisms underlying a variety of medicines used in TCM.

## Data Availability

The datasets presented in this study can be found in the Supplementary Material (Supplementary Material S1, Supplementary Tables S1–15, and Supplementary Figures S1-19) and the mass spectrometry proteomics data have been deposited to the ProteomeXchange via the iProX partner repository with the dataset identifier PXD024535 (http://proteomecentral.proteomexchange.org).
