# Supplementary material for: Application of Proteomics and Metabonomics to Reveal the Molecular Basis of Atractylodis Macrocephalae Rhizome for Ameliorating Hypothyroidism Instead of Hyperthyroidism
Source: Front Pharmacol. 2021 Apr 20;12:664319. doi: 10.3389/fphar.2021.664319 (PMC8095350; doi:10.3389/fphar.2021.664319)
Supplement: Supplementary file 1 [file DataSheet3.docx]

| 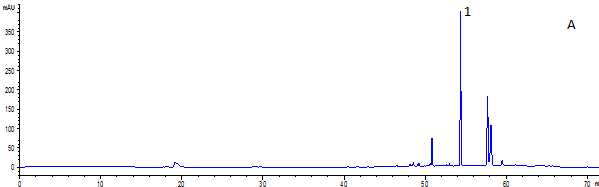 | 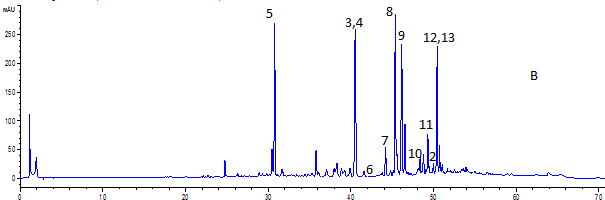 |
| --- | --- |
| 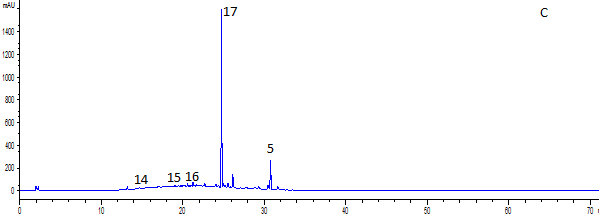 | 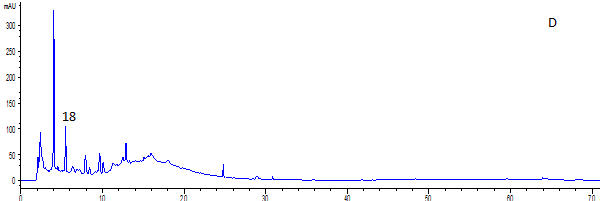 |

*t* /min *t* /min

Figures S1. HPLC characterization of the splitted fractions of AMR. A. VOF; B. LAF; C. ATF; D. OSF. In the study, 18 chemical constituents of AMR were isolated as follow: 1. Atractylone; 2. Juniper camphor; 3. AtractylenolideⅢ; 4. Taraxeryl acetate; 5. (4E, 6E, 12E)-tetradeca-4, 6, 12-trien-8, 10-diyne-1, 3, 14-triol; 6. 3β-acetoxy-atractylenolide Ⅰ; 7. Stigmasterol; 8. Atractylenolide Ⅰ; 9. Isoatractylenolide Ⅰ; 10. Sitosterin; 11. Atractylenolide Ⅱ; 12. Dibutyl phthalate; 13. Diisobutyl phthalate; 14. Atractyloside A; 15. Caprolactam; 16. 5-Hydroxymethyl furfural ether; 17. (4E, 6E, 12E)-3, 14-dihydroxytetradeca-4, 6, 12-trien-8, 10-diyn-1-yl acetate; 18. 5-Hydroxymethyl furfural.

**Figure S2 Variation of body weight and rectal temperature in hypothyroidism and hyperthyroidism rats**

Note: A/C was under hypothyroidism rat; B/D was under hypothyroidism rat; **p*<0.05 and ***p*<0.01compared to MO using LSD analyse.


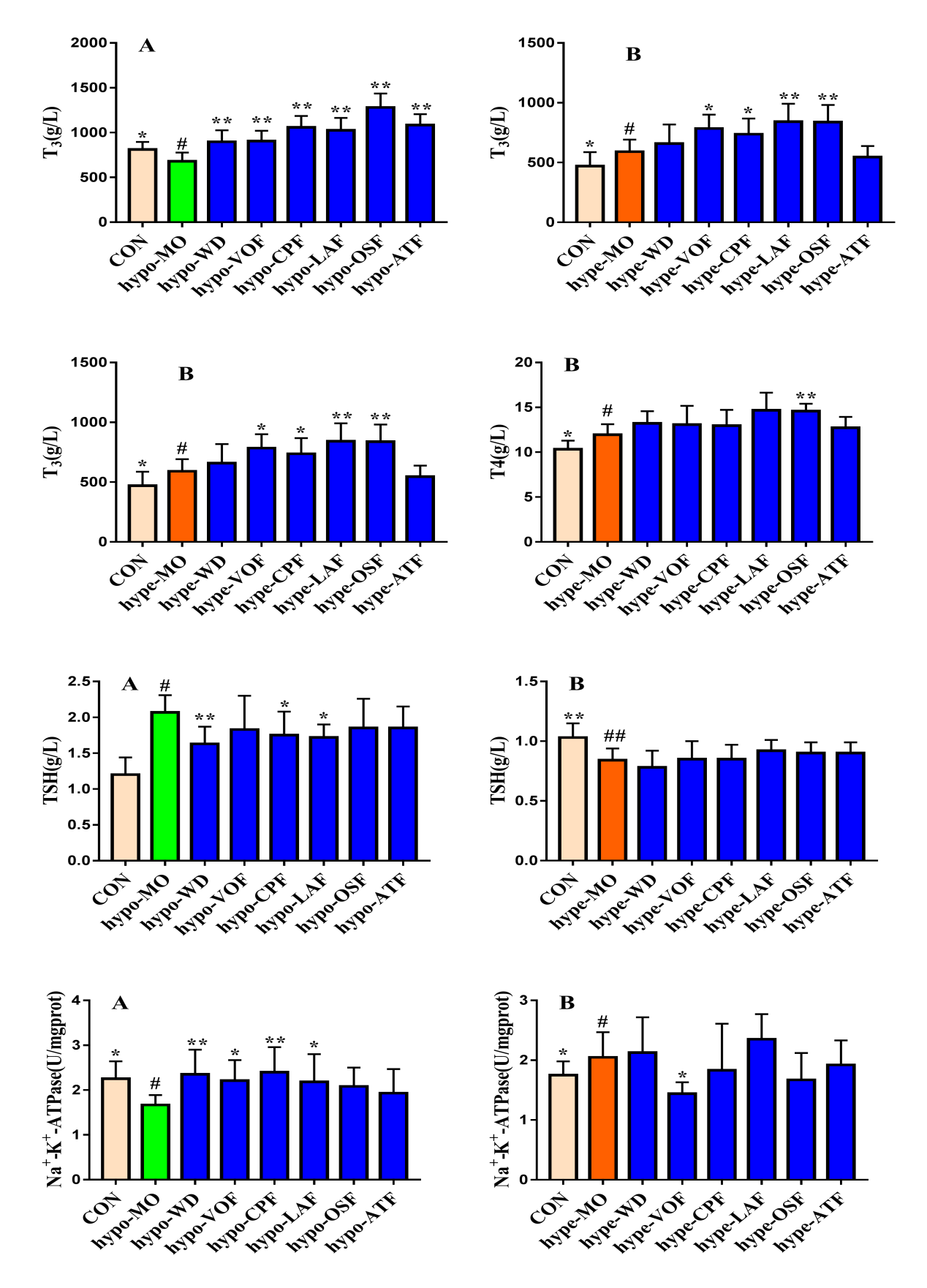


**Figure S3** **Variation of T_3_, T_4_, TSH and Na^+^-K^+^-ATPase in hypothyroidism and hyperthyroidism rats**

Note: A was under hypothyroidism rats; B was under hypothyroidism rats; *P<0.05 and **P<0.01compared to MO using LSD analyse. #*P*<0.05 and ##*P*<0.01compared to MO using LSD analyse.


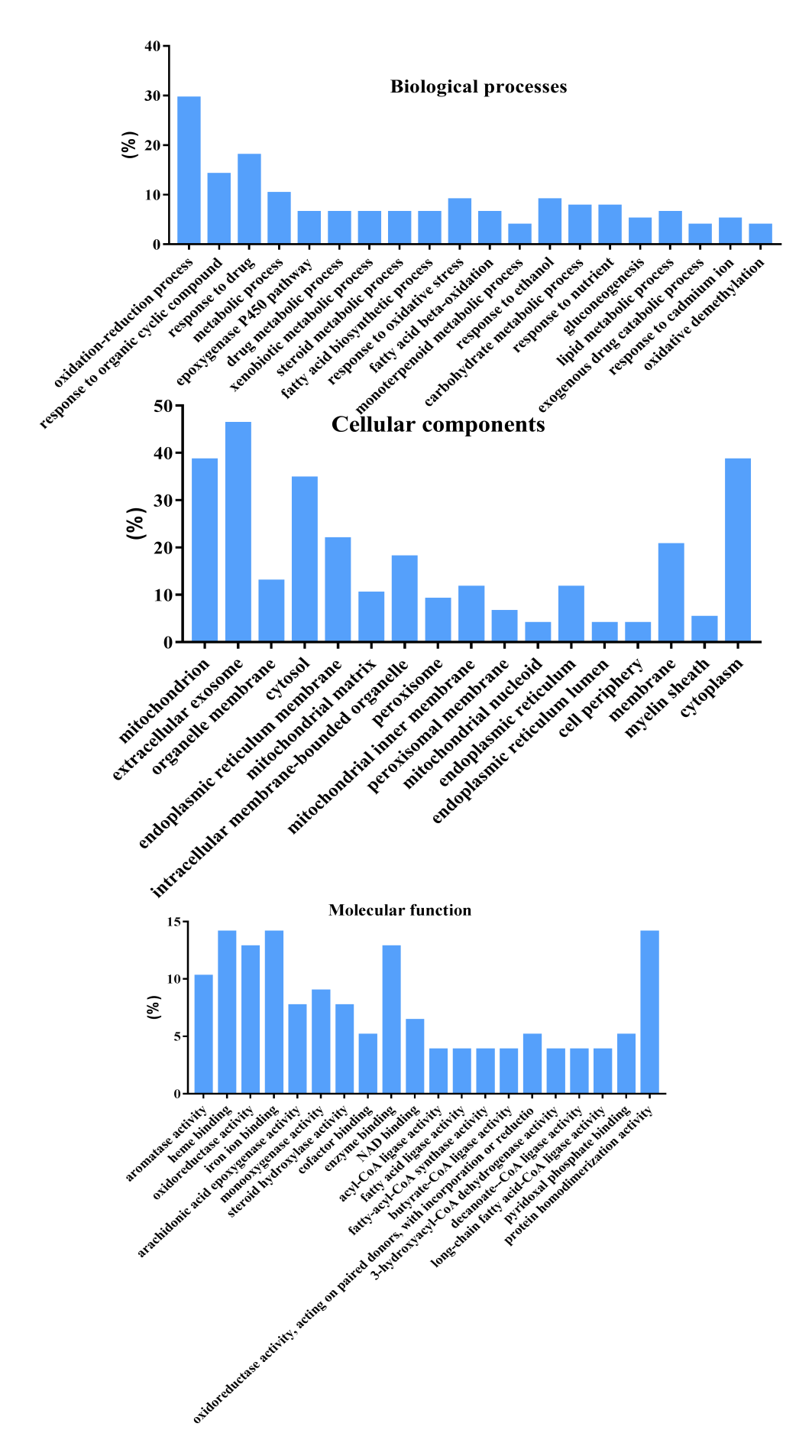


**Figure S4** **The proportion of the differentially expressed proteins categorized by function (MO *vs* CON).**

**
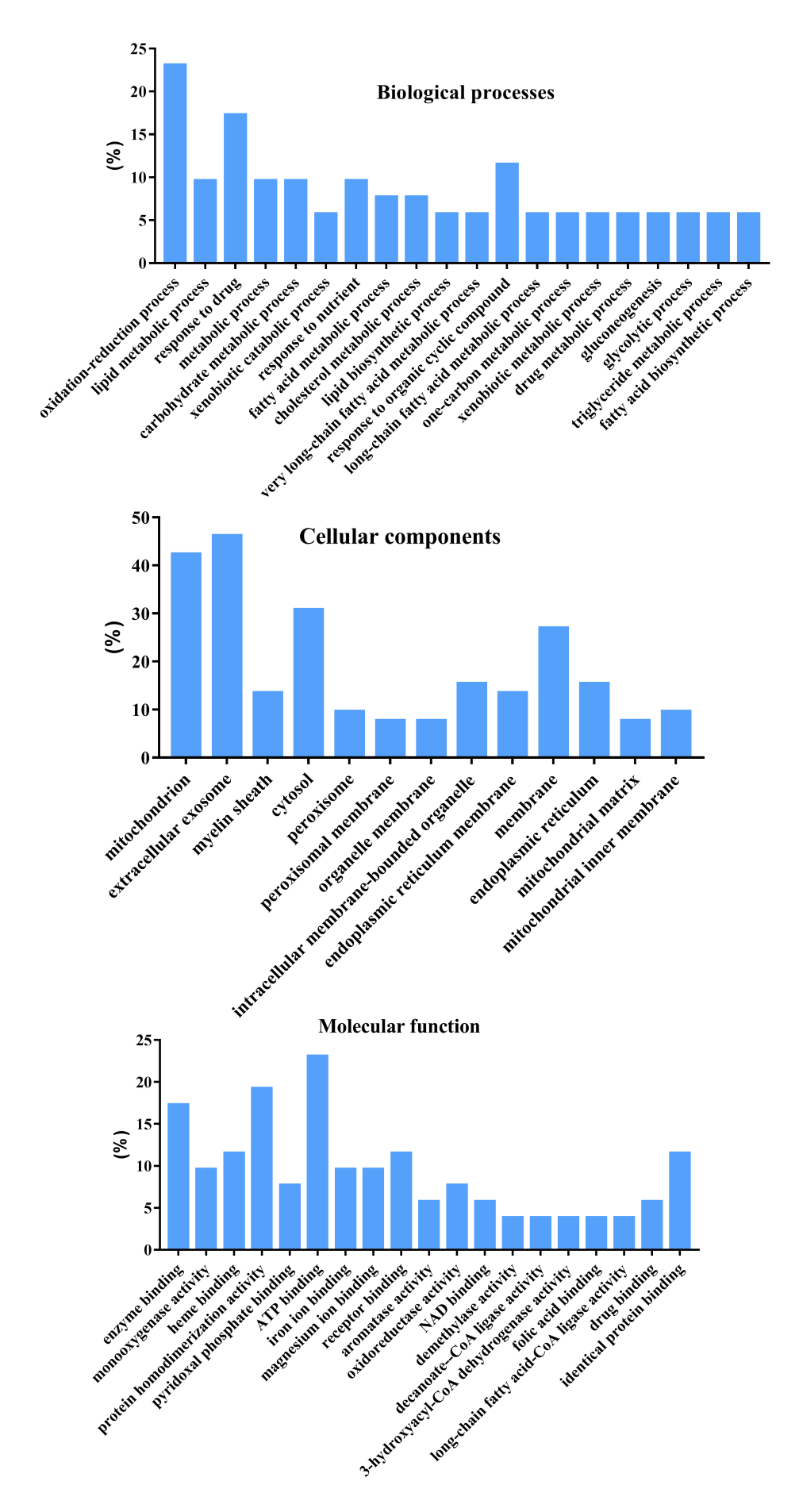
**

**Figure S5** **The proportion of the differentially expressed proteins categorized by function (MO *vs* AMR).**


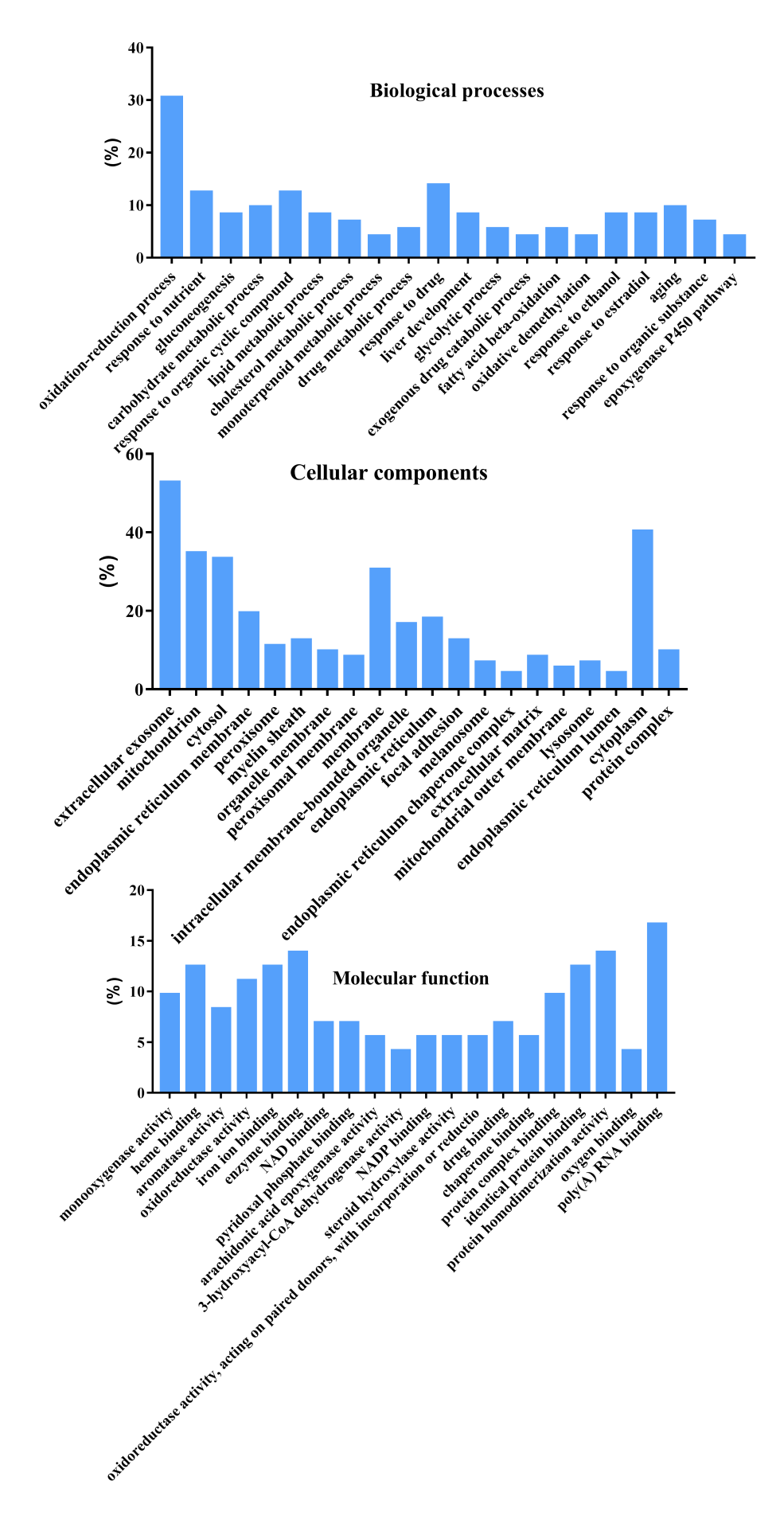


**Figure S6** **The proportion of the differentially expressed proteins categorized by function (MO *vs* VOF).**


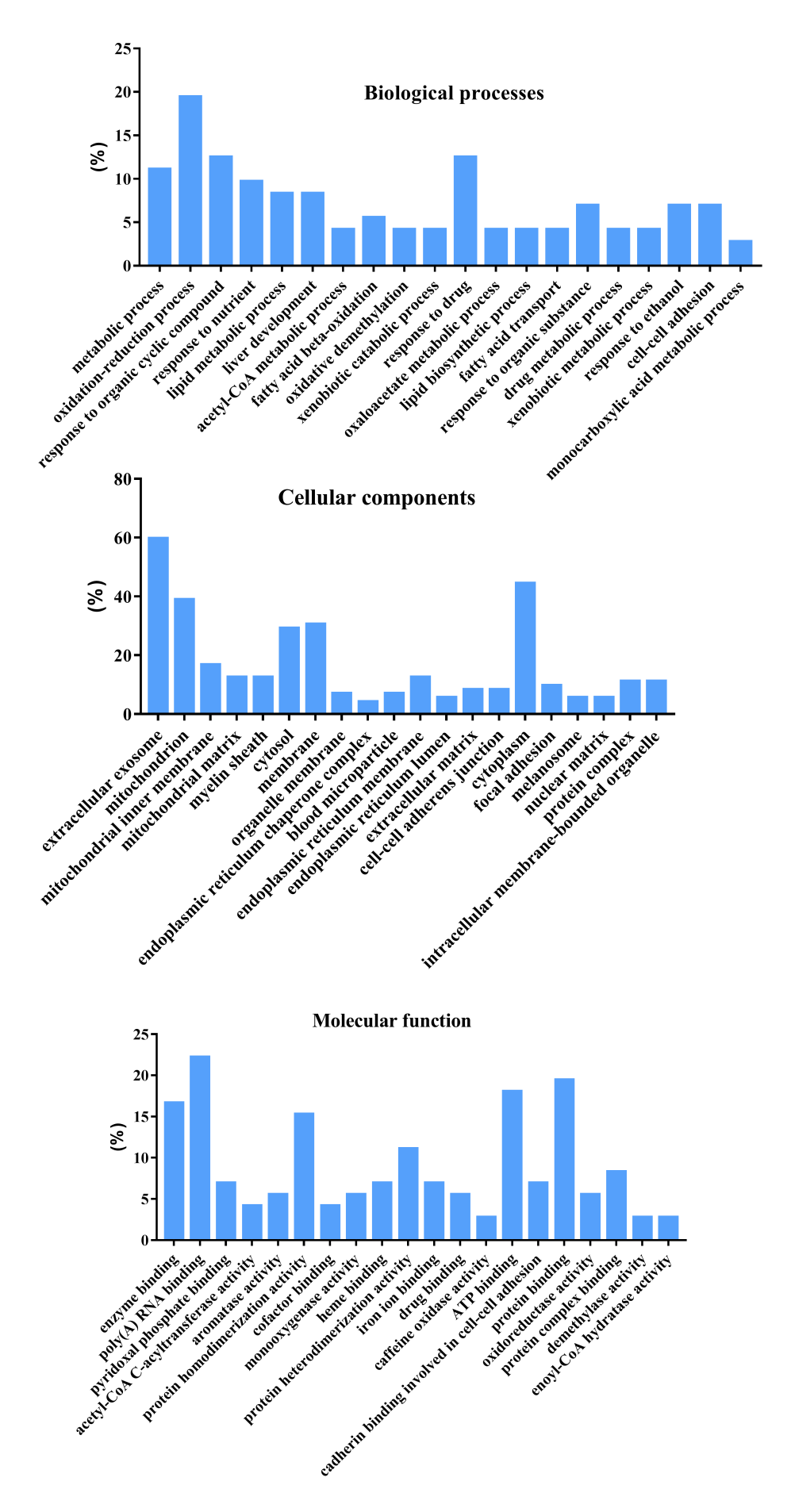


**Figure S7** **The proportion of the differentially expressed proteins categorized by function (MO *vs* CPF).**


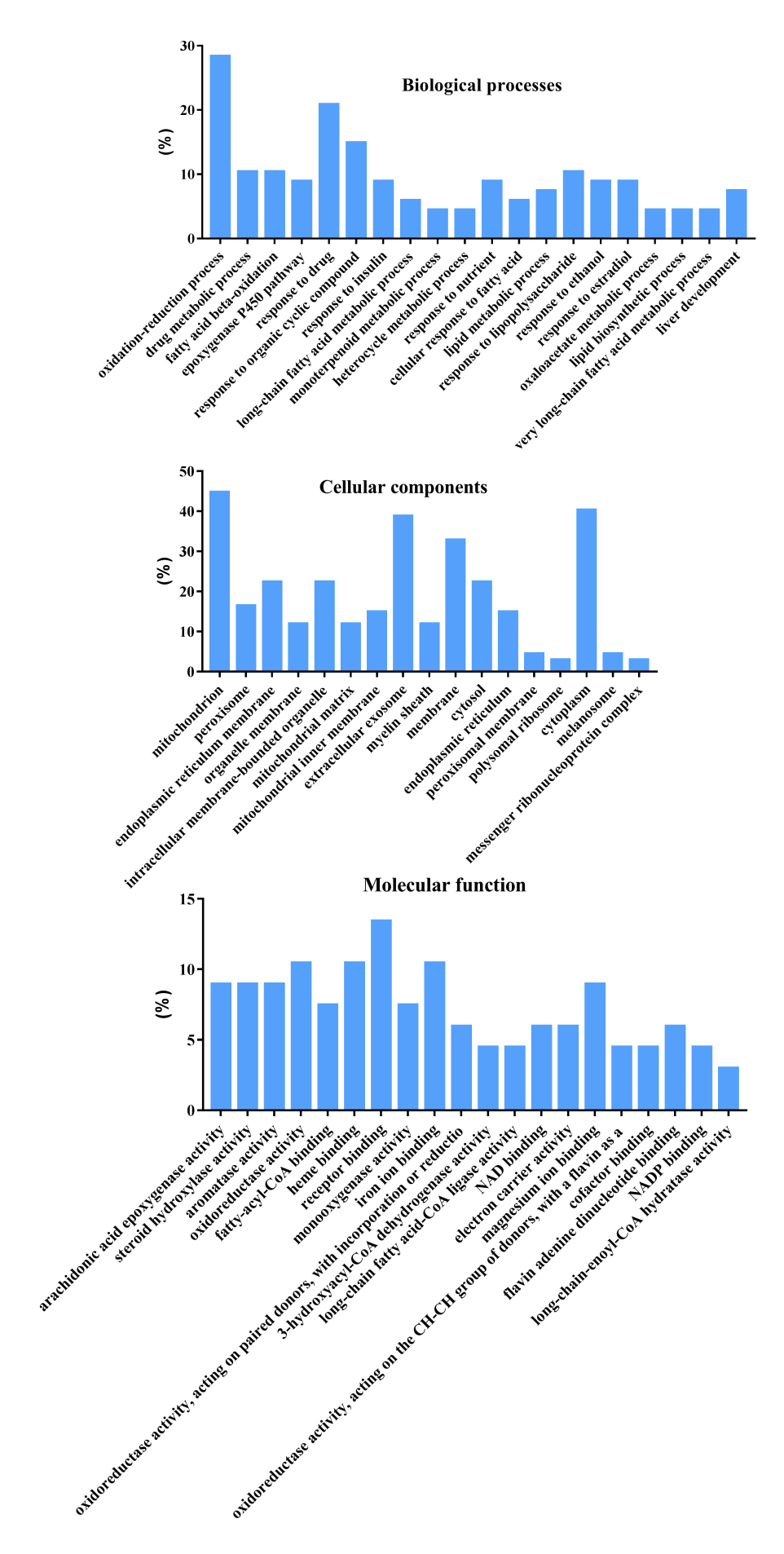


**Figure S8** **The proportion of the differentially expressed proteins categorized by function (MO *vs* LAF).
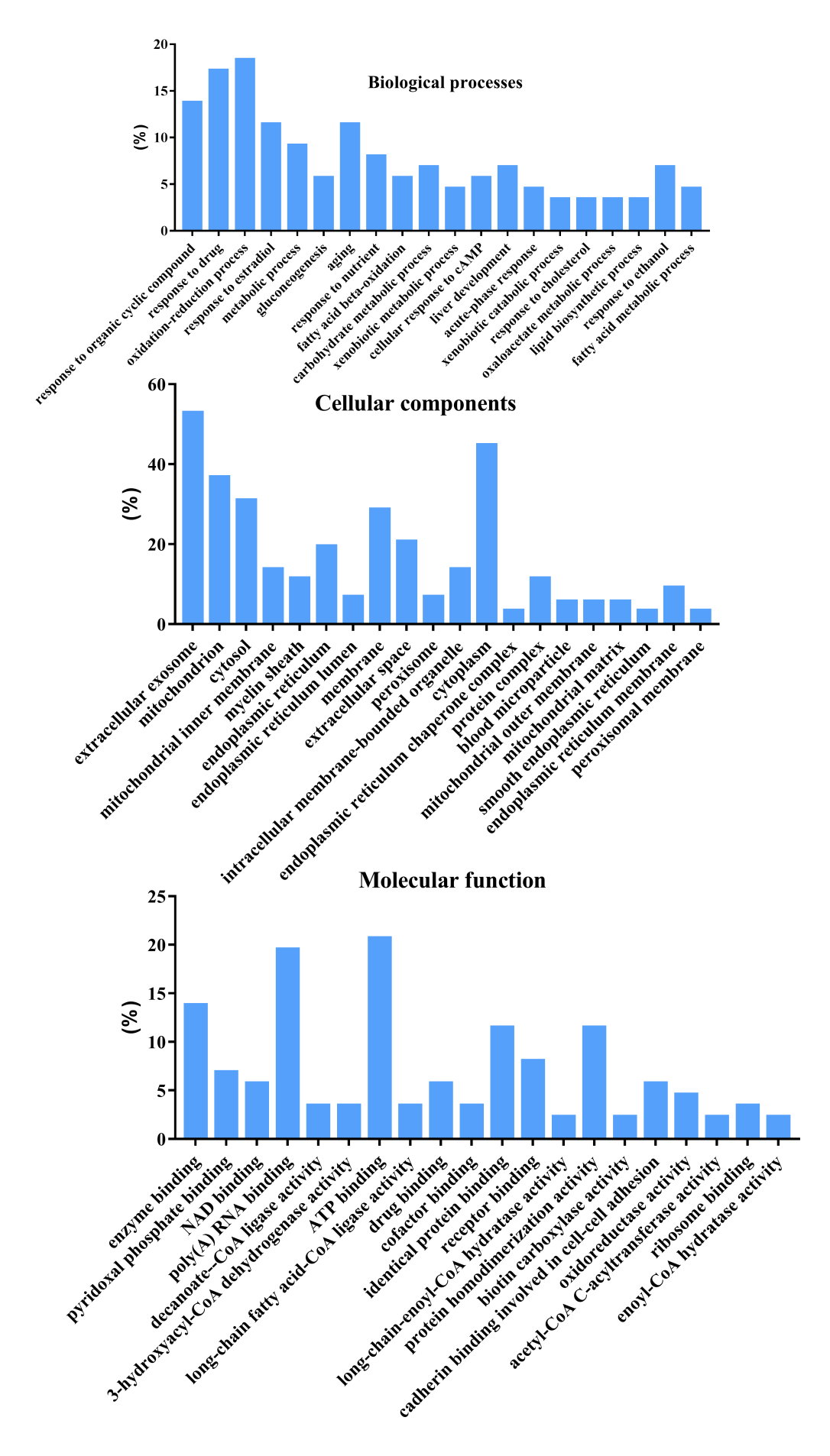
**

**Figure S9** **The proportion of the differentially expressed proteins categorized by function (MO *vs* OSF).** **
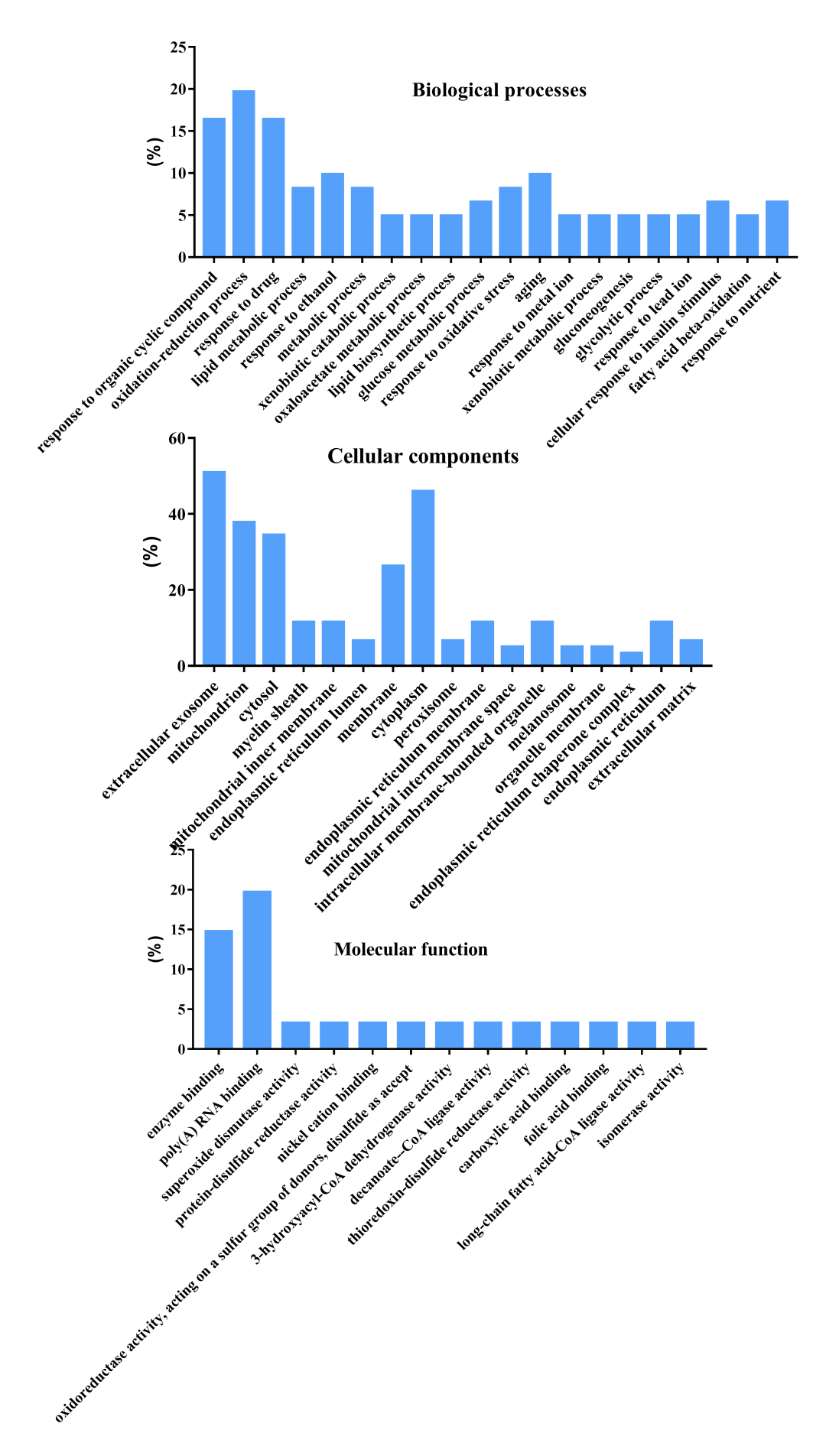
**

**Figure S10** **The proportion of the differentially expressed proteins categorized by function (MO *vs* ATF).**


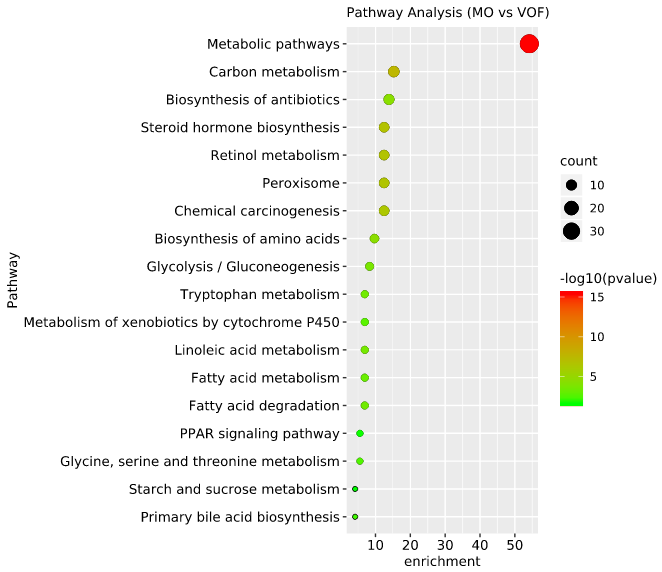

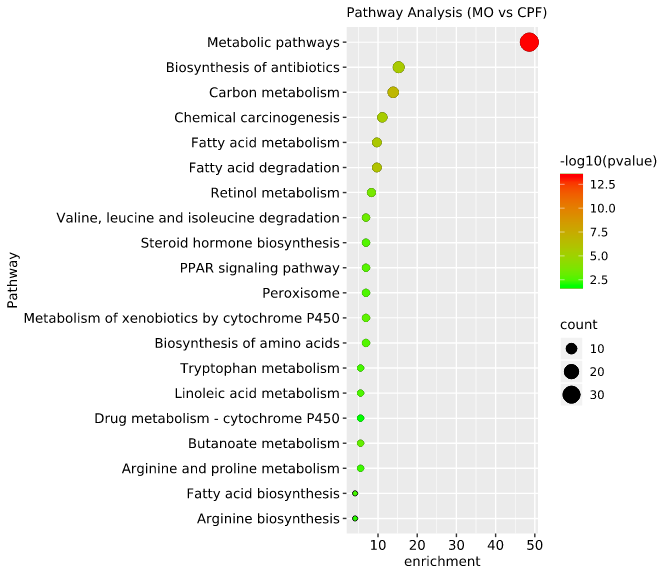


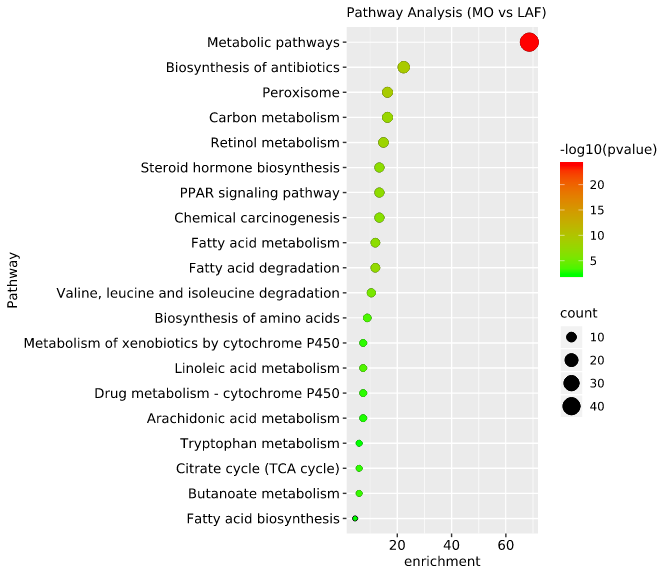

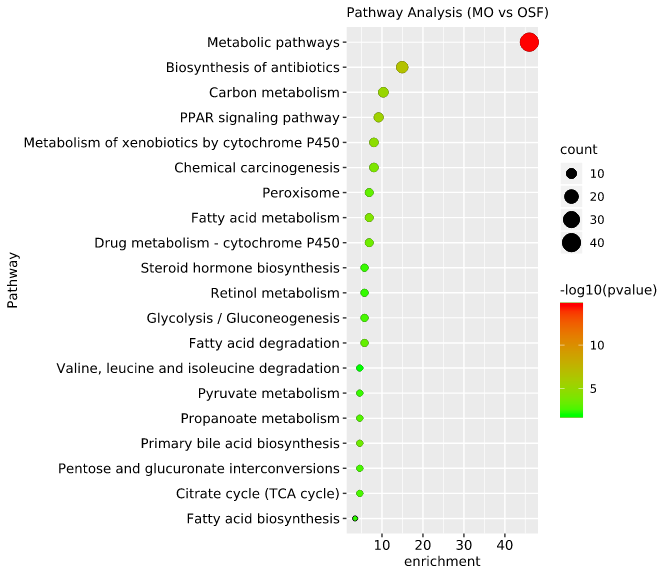

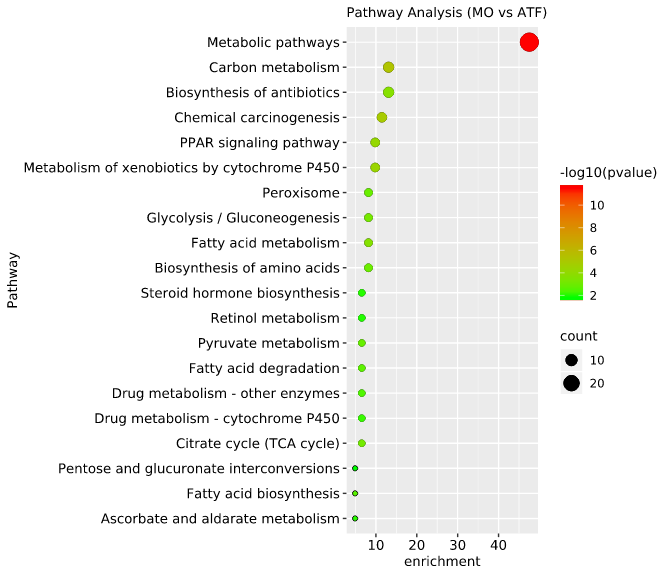


**Figures S11 pathway analysis of DEPs**


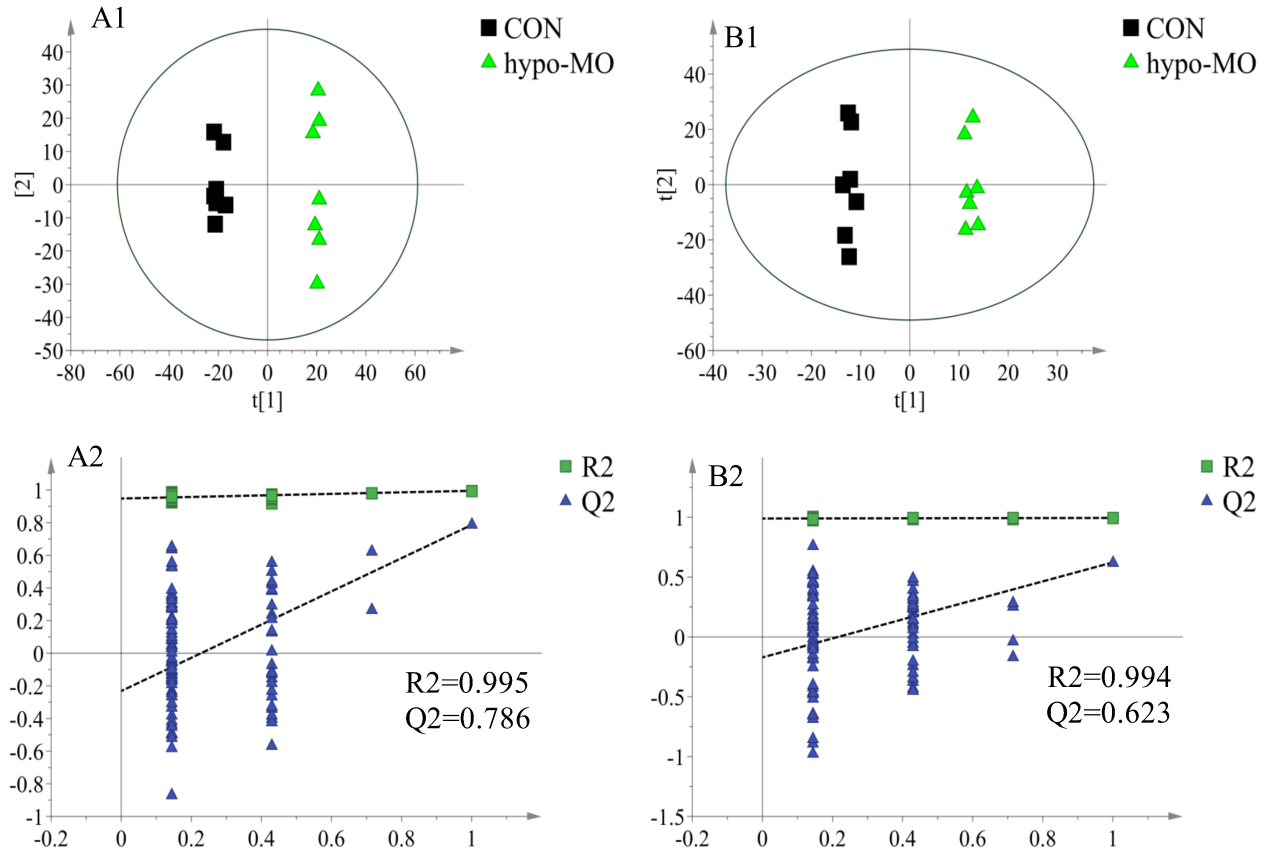


**Figures S12** OPLS-DA (A1, B1) score plots of hypothyroidism rat urine metabolites from the normal group (n = 7) and model group (n = 7) and the permutation tests

(A2, B2) for OPLS-DA models in positive ion mode (A) and negative ion mode (B). The OPLS-DA score plots indicated a distinct separation between normal group and

model group. Validation of the OPLS-DA models was randomly permutated for 100 times and the x-axis represents the correlation coefficient between the original y

variable and the permutated y variable while the y-axis represents the value of R2 and Q2.

| 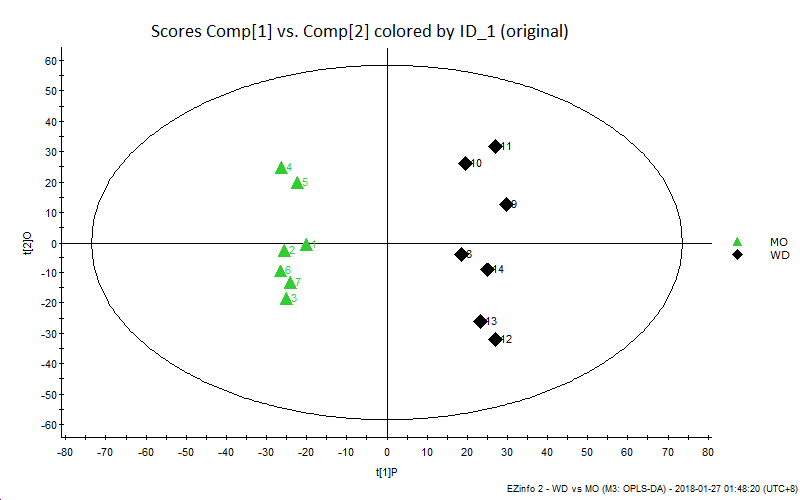A. hypo-MO *vs* hypo-WD in positive ion mode | 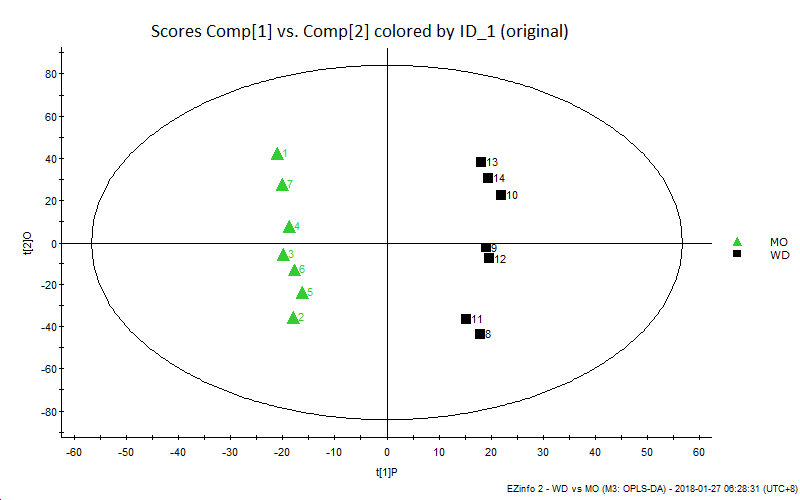 B. hypo-MO *vs* hypo-WD negative ion mode |
| --- | --- |
| 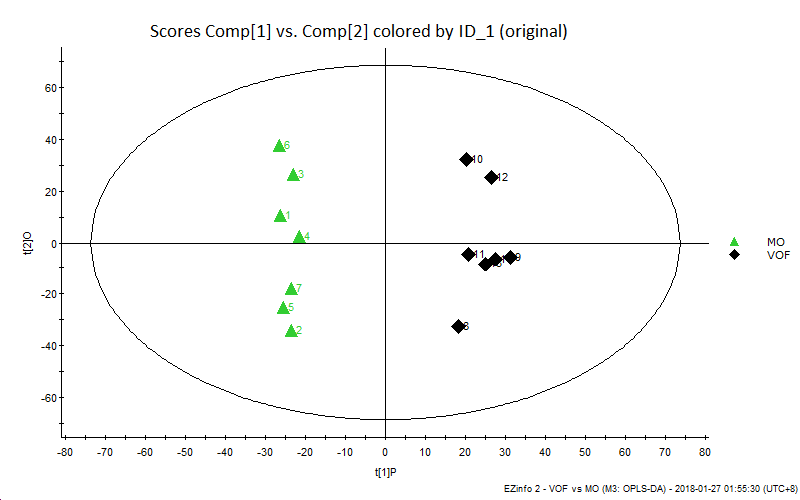C. hypo-MO *vs* hypo-VOF in positive ion mode | 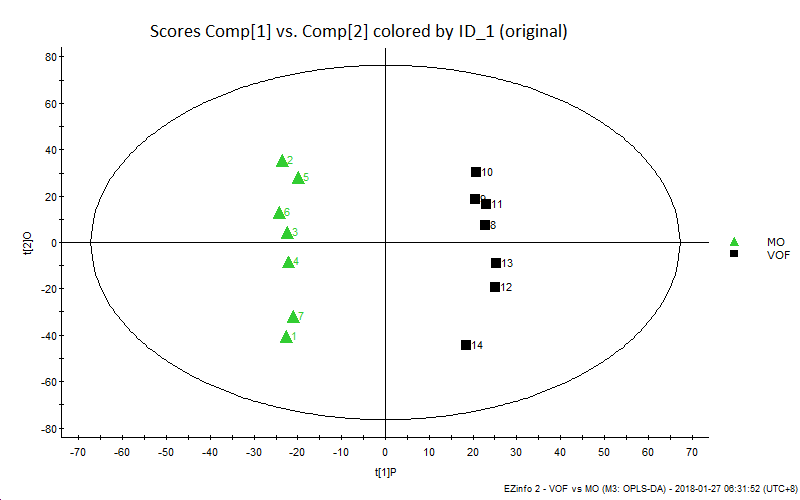 D. hypo-MO *vs* hypo-VOF negative ion mode |
| 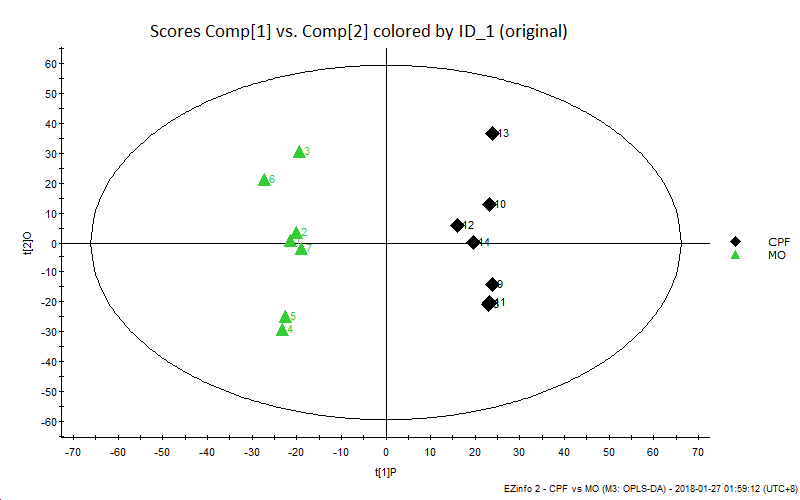 E. hypo-MO *vs* hypo-CPF in positive ion mode | 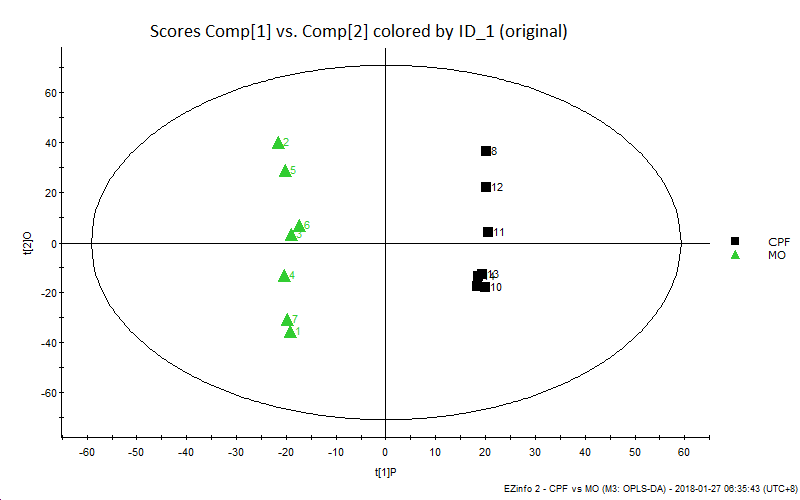 F. hypo-MO *vs* hypo-CPF in negative ion mode |
| 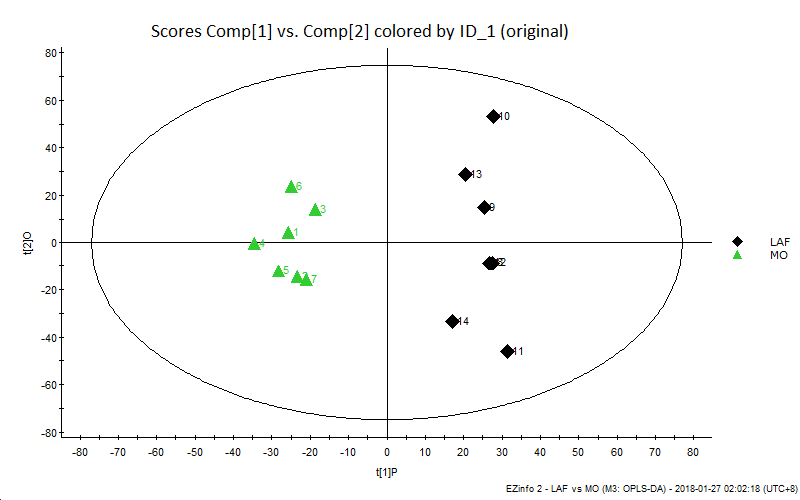 G. hypo-MO *vs* hypo-LAF in positive ion mode | 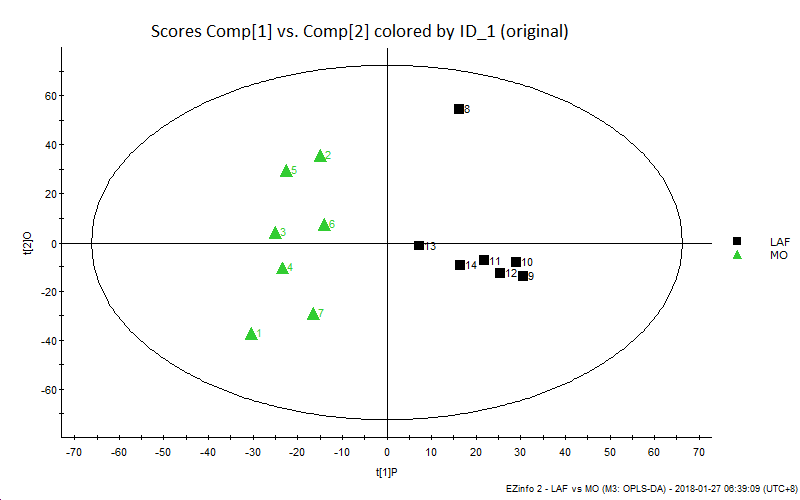 H. hypo-MO *vs* hypo-LAF in negative ion mode |
| 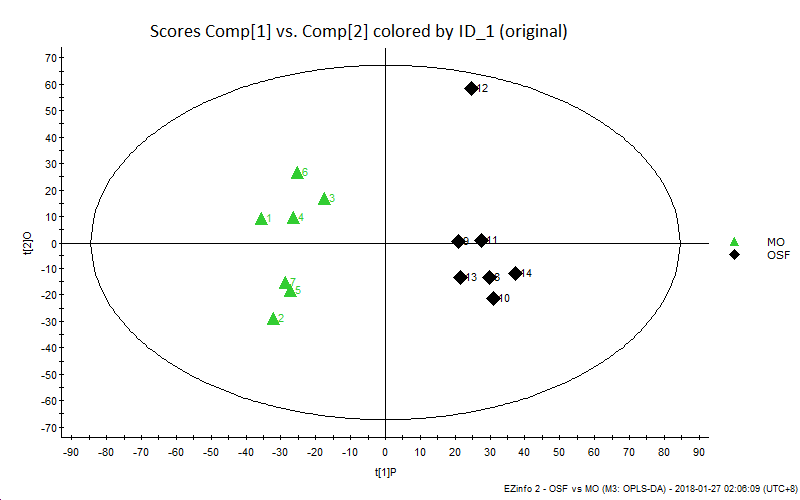 I. hypo-MO *vs* hypo-OSF in positive ion mode | 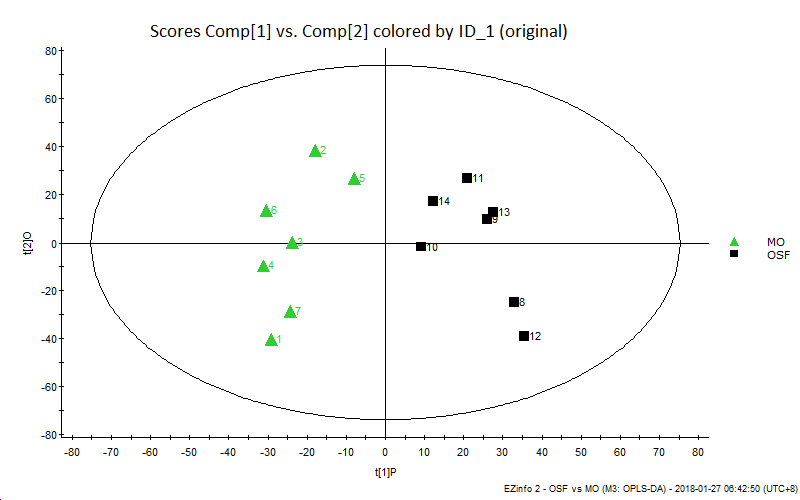 J. hypo-MO *vs* hypo-OSF in negative ion mode |
| 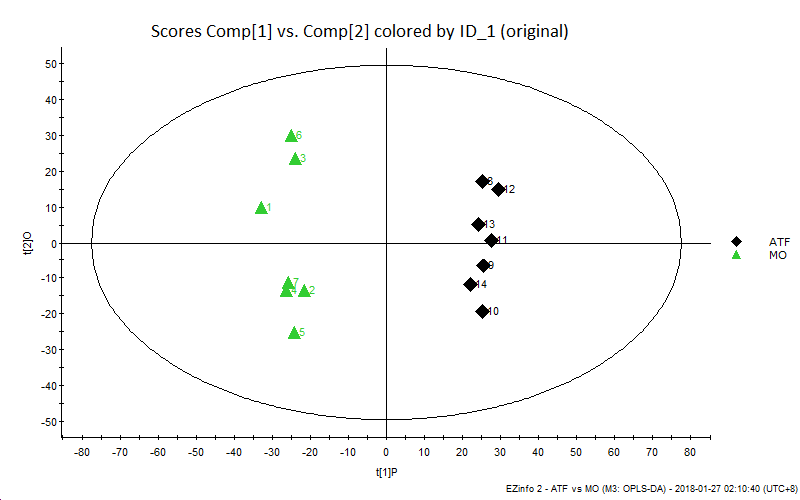 K. hypo-MO *vs* hypo-ATF in positive ion mode | 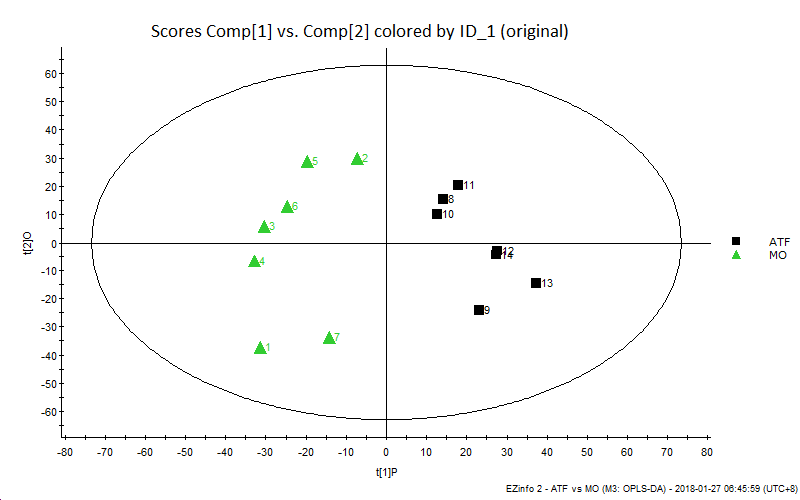 L. hypo-MO *vs* hypo-ATF in negative ion mode |

**Figures** **S13 OPLS-DA scores plot of the UHPLC/TOF-MS spectral from MO and AMR and its fraction group in hypothyroidism**

**
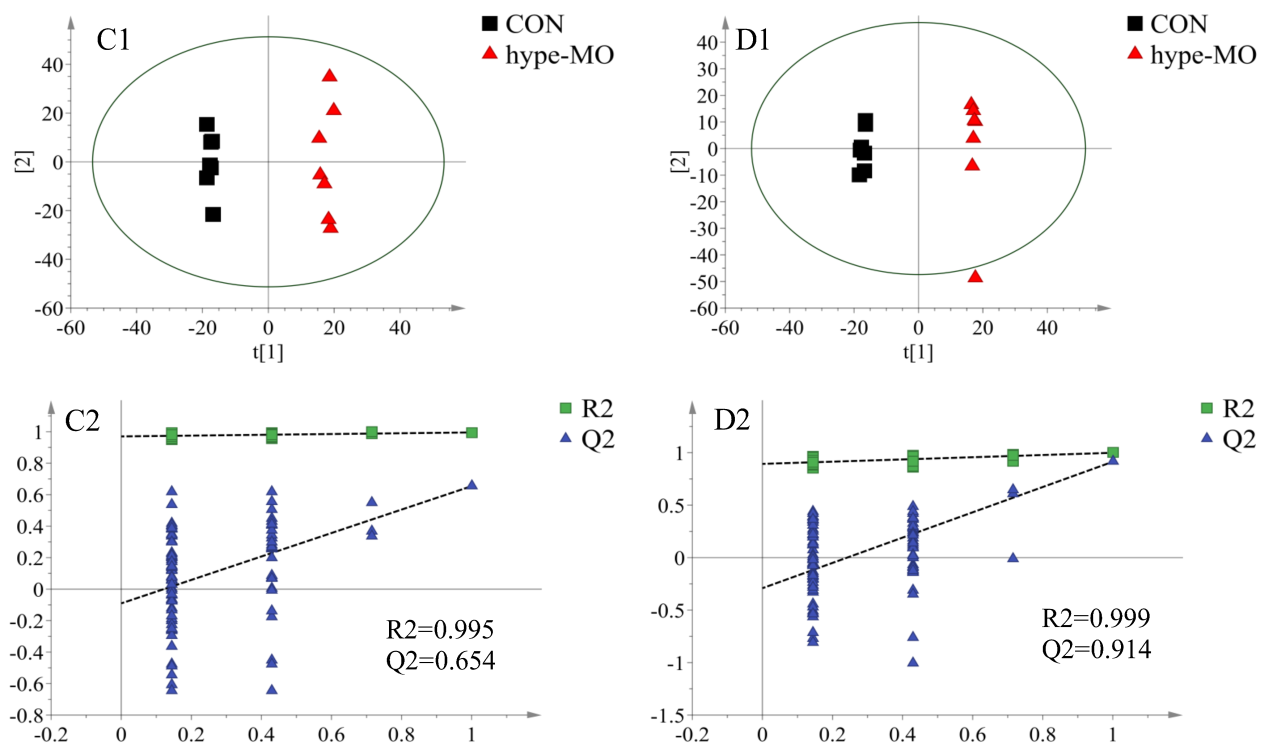
**

**FIGURE S14** OPLS-DA (C1, D1) score plots of hyperthyroidism rat urine metabolites from the normal group (n = 7) andmodel group (n = 7) and the permutation tests (C2, D2) for OPLS-DA models in positive ion mode (C) and negative ion mode (D). The OPLS-DA score plots indicated a distinct separation between normal group and model group. Validation of the OPLS-DA models was randomly permutated for 100 times and the x-axis represents the correlation coefficient between the original y variable and the permutated y variable while the y-axis represents the value of R2 and Q2.

| 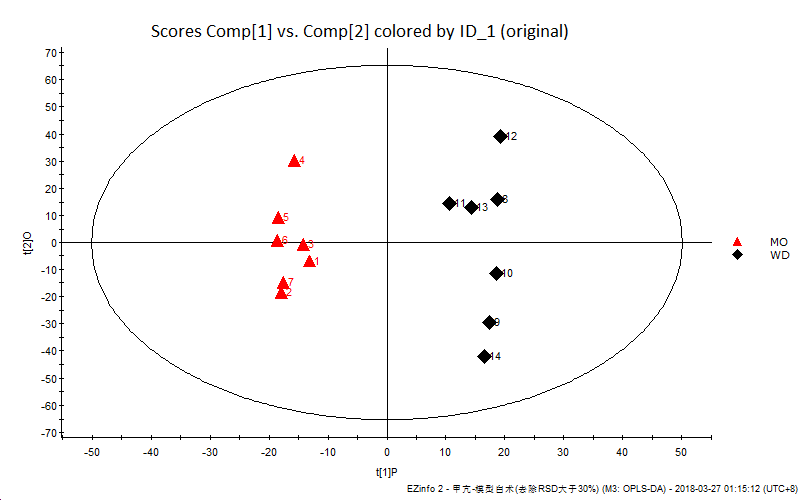 A. hype-MO *vs* hype-WD in positive ion mode | 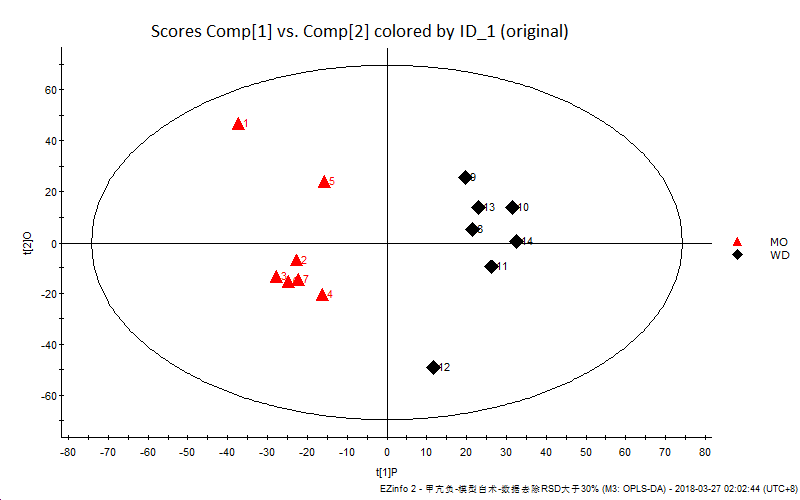 B. hype-MO *vs* hype-WD in negative ion mode |
| --- | --- |
| 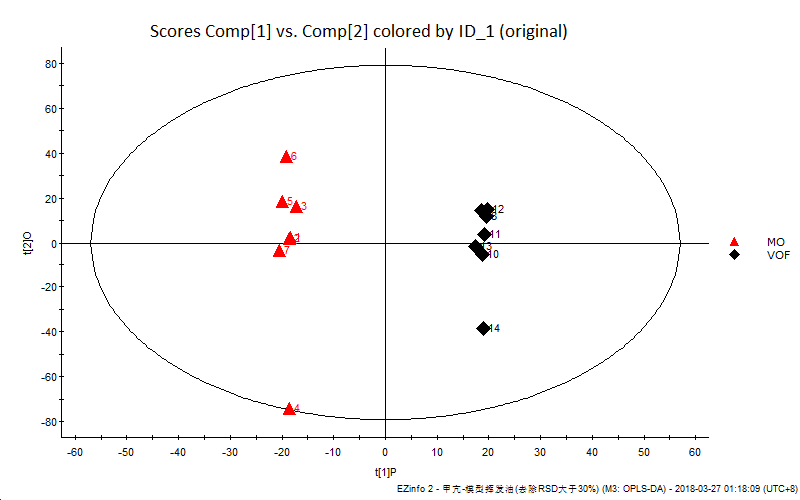 C. hype-MO *vs* hype-VOF in positive ion mode | 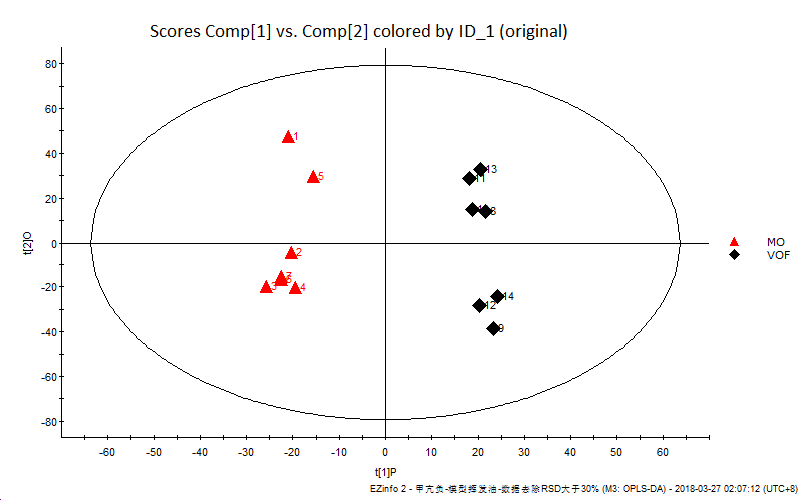 D. hype-MO *vs* hype-VOF in negative ion mode |
| 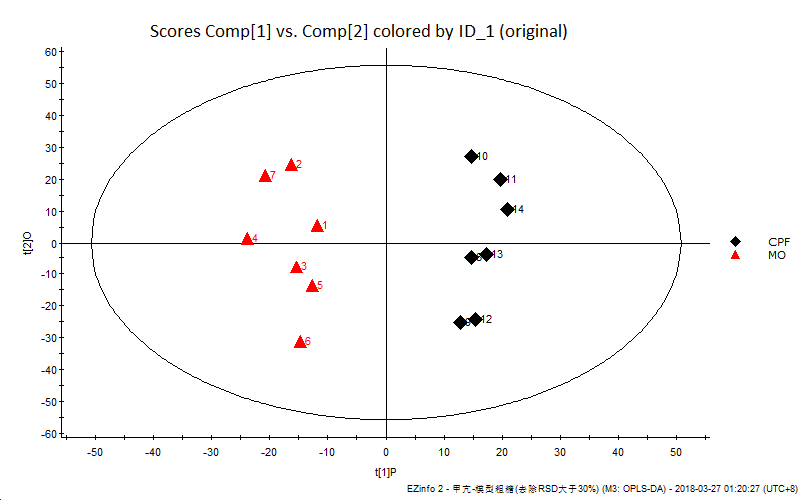 E. hype-MO *vs* hype-CPF in positive ion mode | 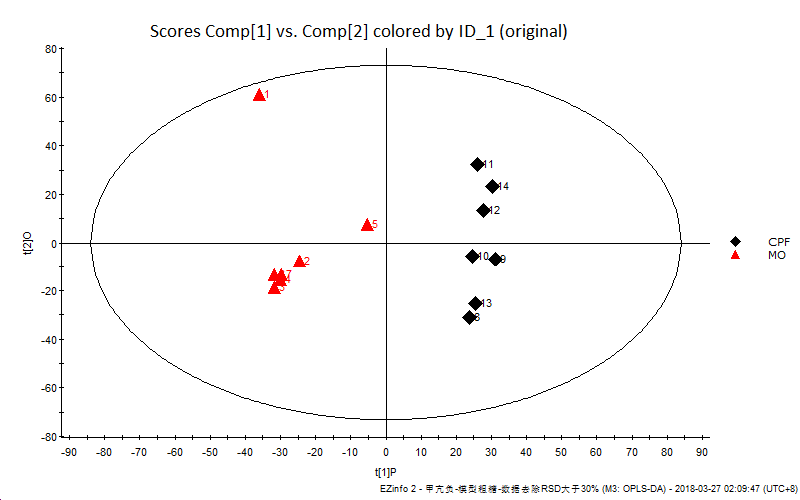 F. hype-MO *vs* hype-CPF in negative ion mode |
| 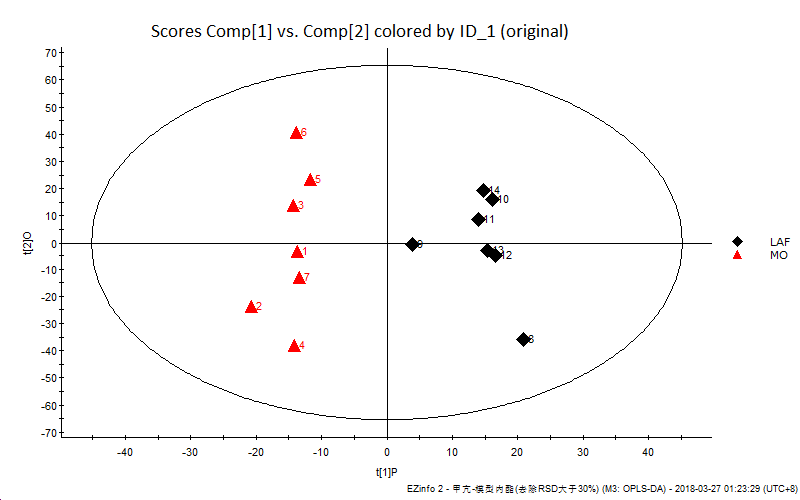 G. hype-MO *vs* hype-LAF in positive ion mode | 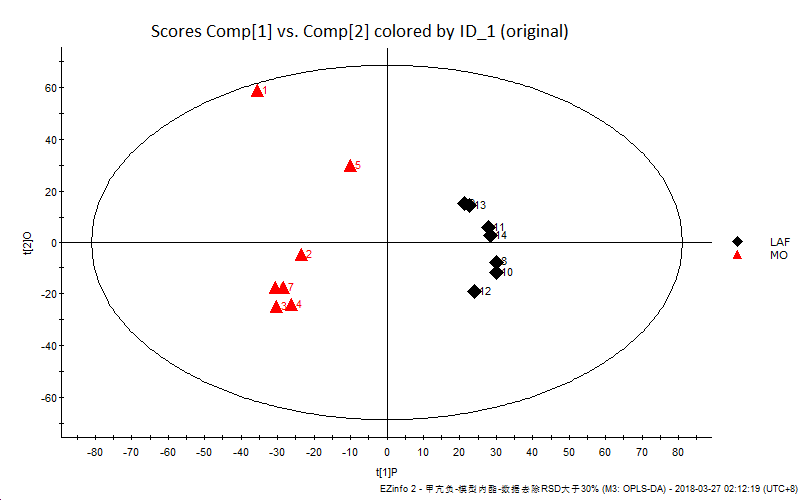H. hype-MO *vs* hype-LAF in negative ion mode |
| 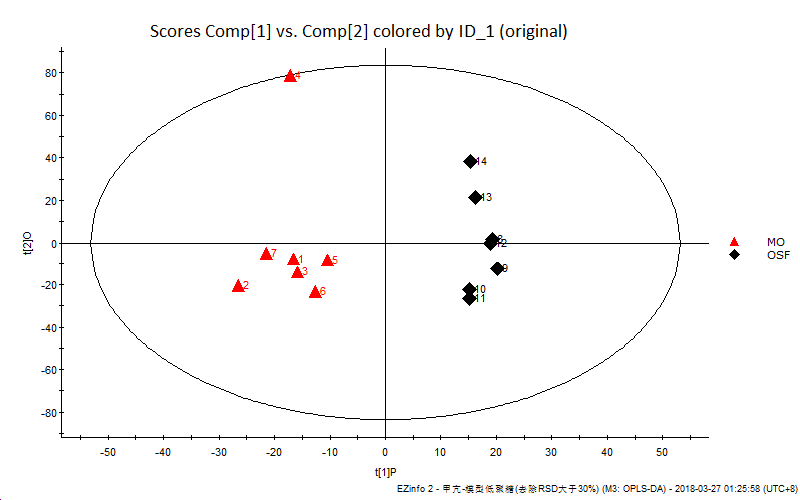I. hype-MO *vs* hype-OSF in positive ion mode | 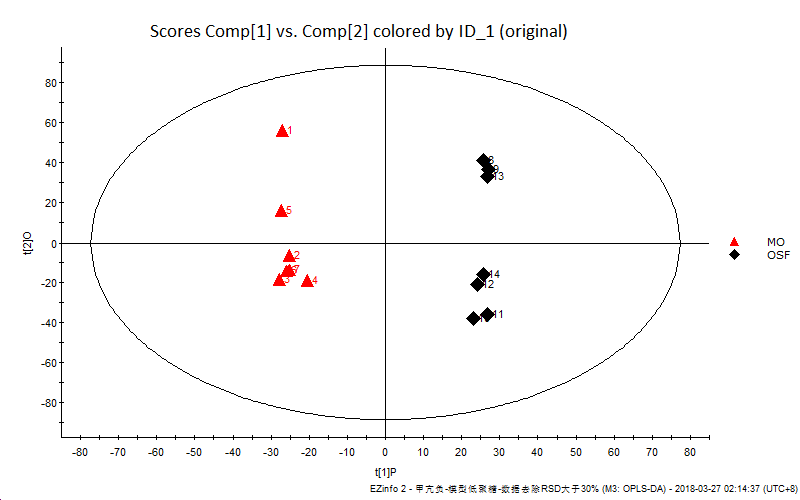J. hype-MO *vs* hype-OSF in negative ion mode |
| 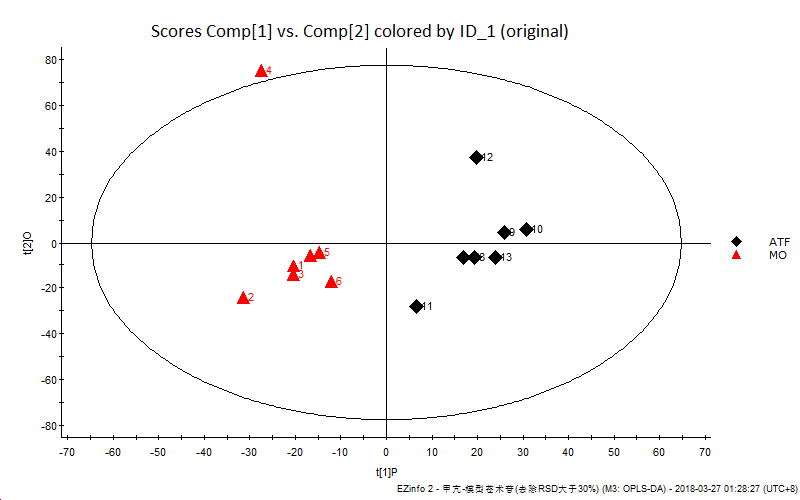 K. hype-MO *vs* hype-ATF in positive ion mode | 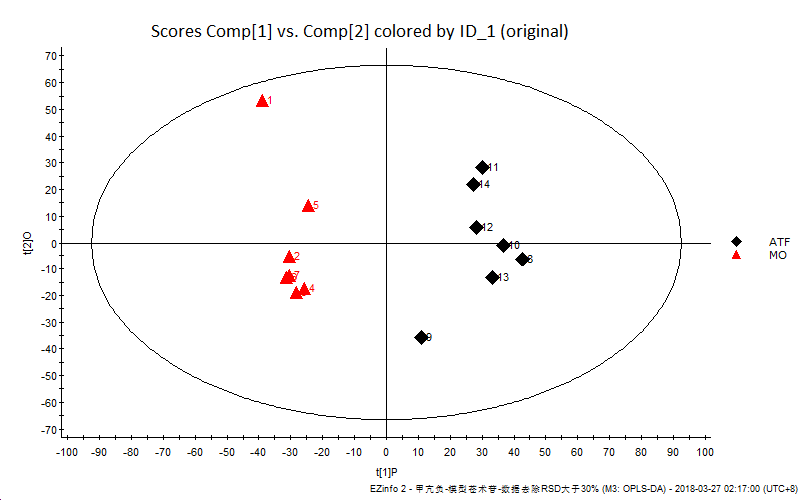 L. hype-MO *vs* hype-ATF in negative ion mode |
| **Figures S15 OPLS-DA scores plot of the UHPLC/TOF-MS spectral from MO and AMR and its fraction group in hyperthyroidism** | |


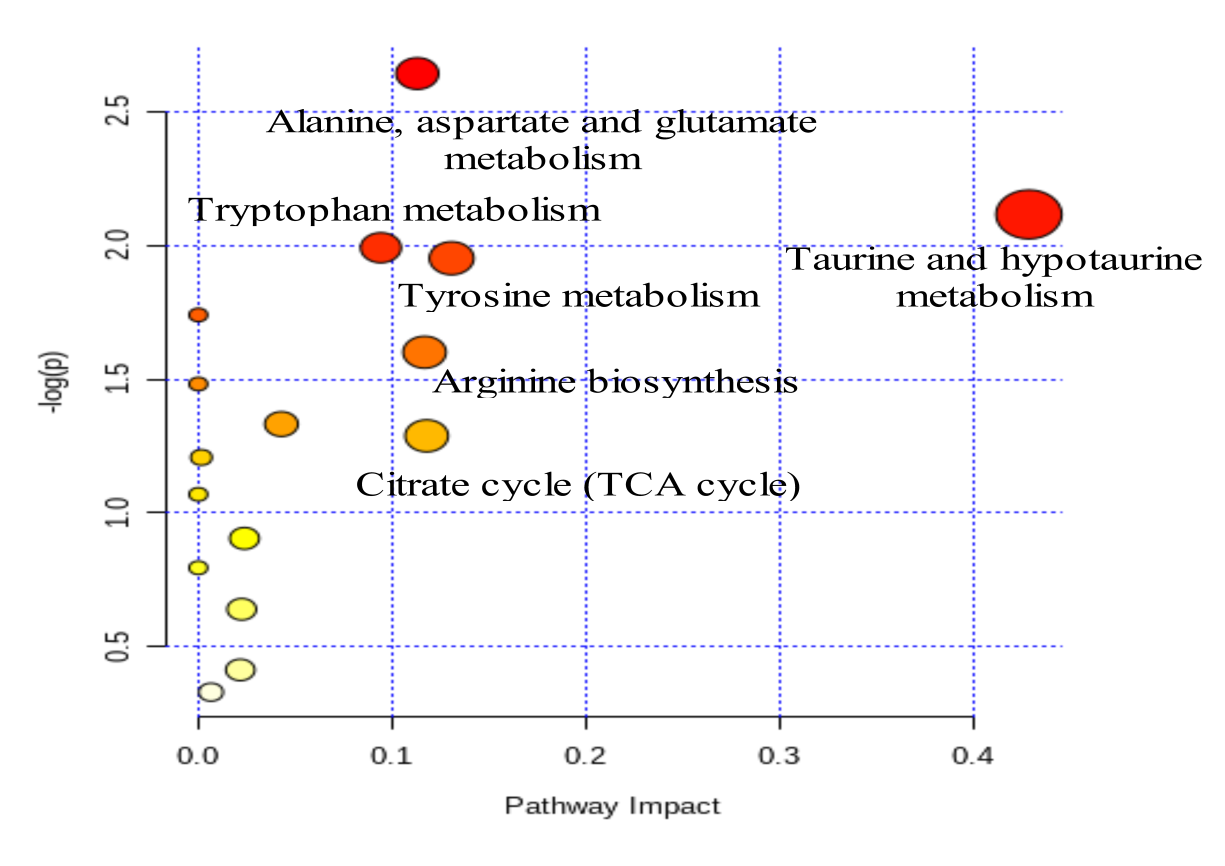


**Figures S16 Pathways enrichment of differential metabolites in hypothyroidism**


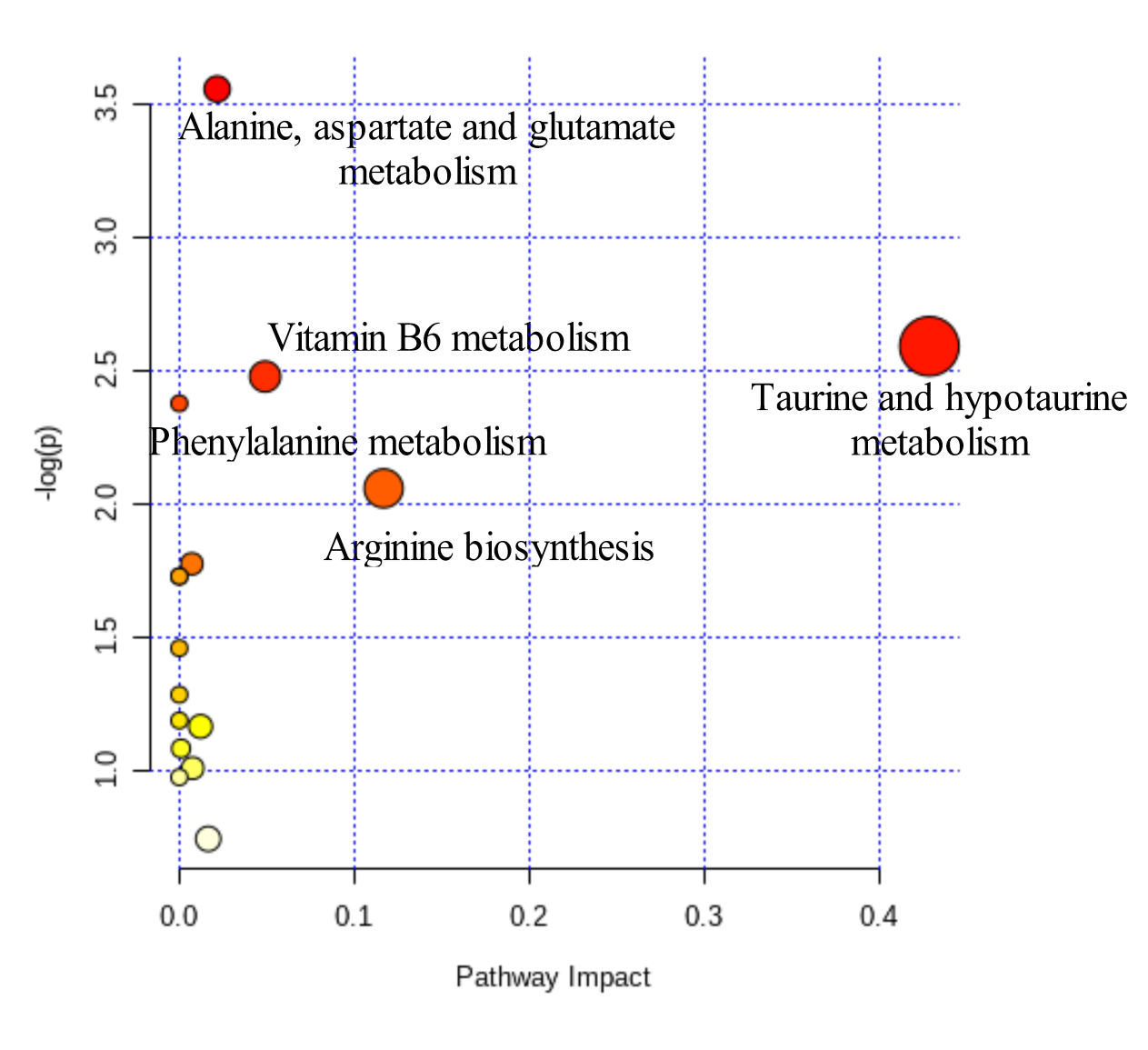


**Figures S17** **Pathways enrichment of differential metabolites in hyperthyroidism**

| 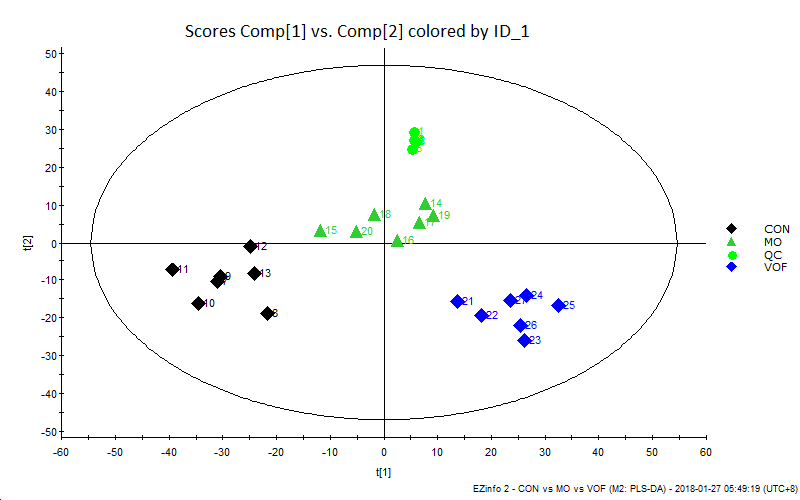A. CON vs hypo-MO *vs* hypo-VOF in positive ion mode | 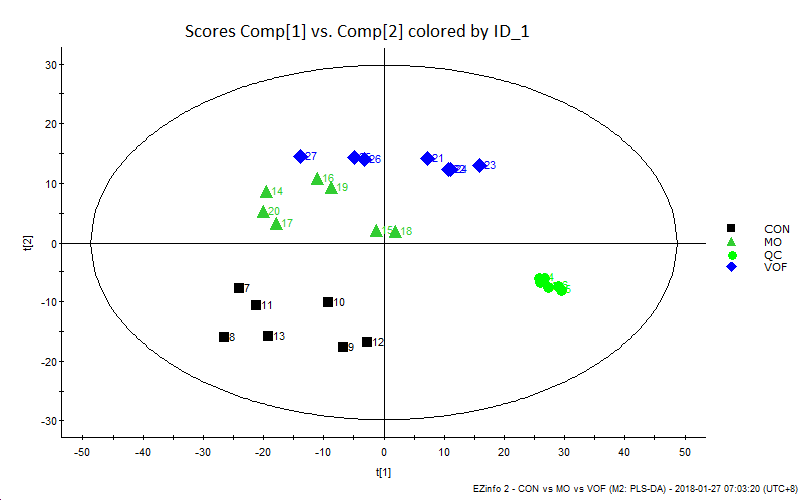 B. CON vs hypo-MO *vs* hypo-VOF negative ion mode |
| --- | --- |
| 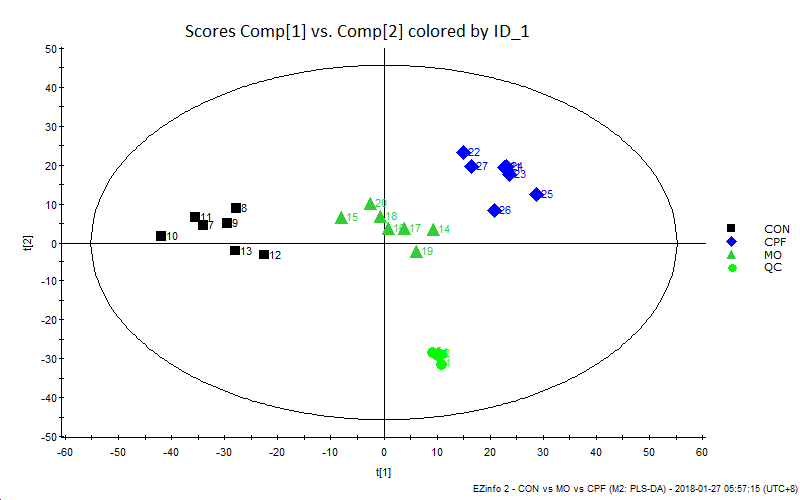C. CON vs hypo-MO *vs* hypo-CPF in positive ion mode | 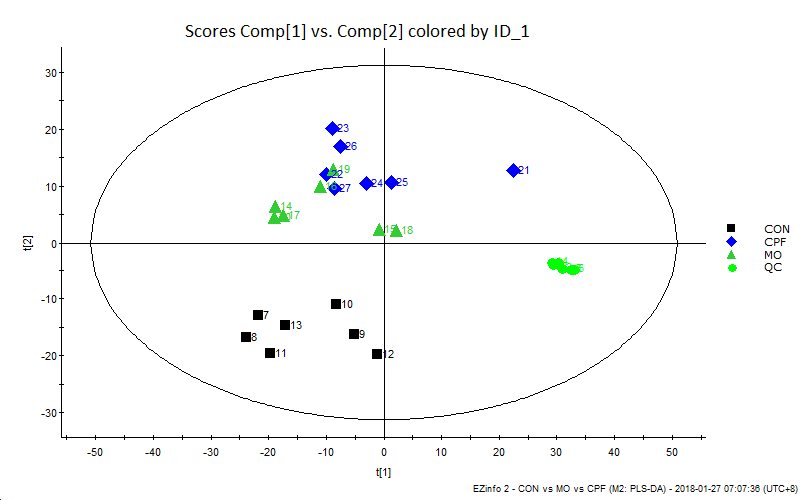D. CON vs hypo-MO *vs* hypo-CPF negative ion mode |
| 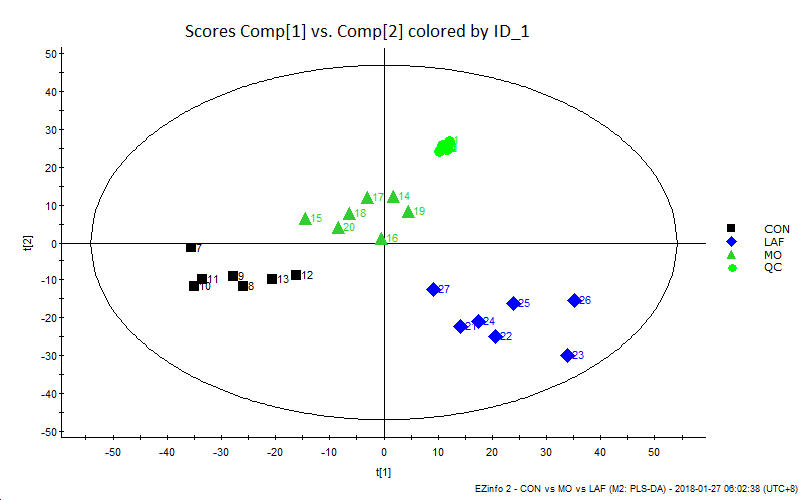 E. CON vs hypo-MO *vs* hypo-LAF in positive ion mode | 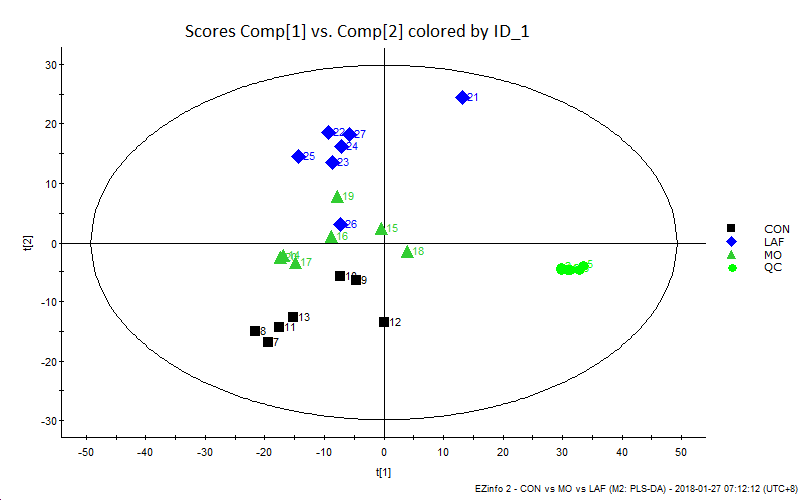 F. CON vs hypo-MO *vs* hypo-LAF in negative ion mode |
| 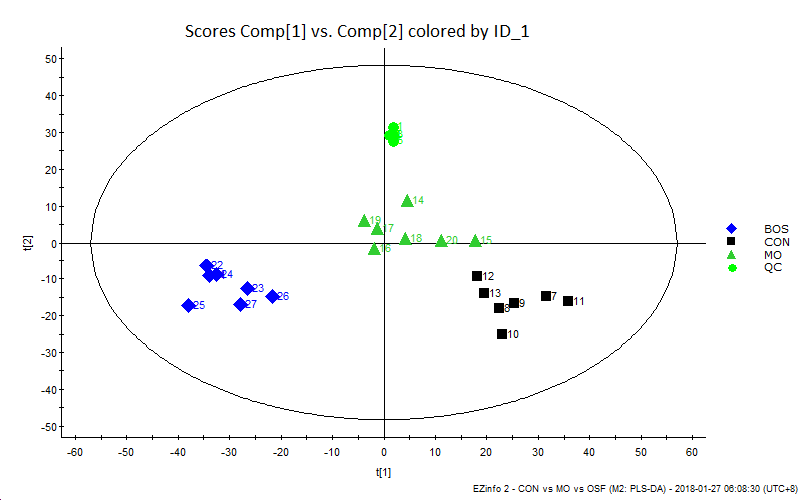G. CON vs hypo-MO *vs* hypo-OSF in positive ion mode | 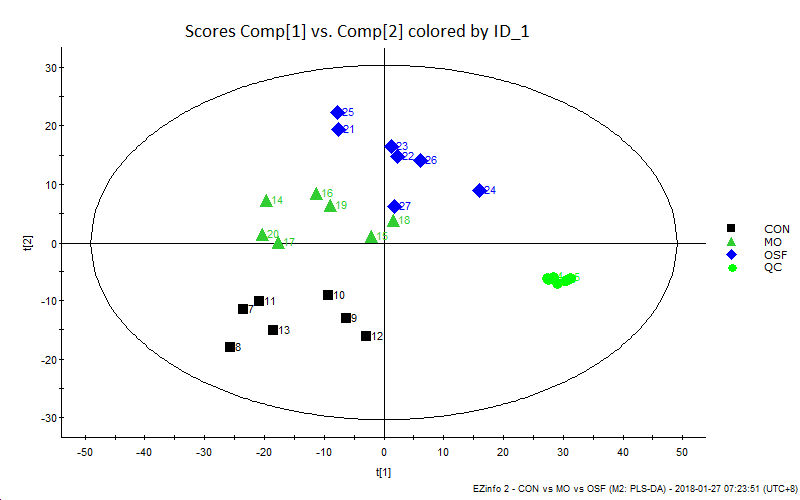H. CON vs hypo-MO *vs* hypo-OSF in negative ion mode |
| 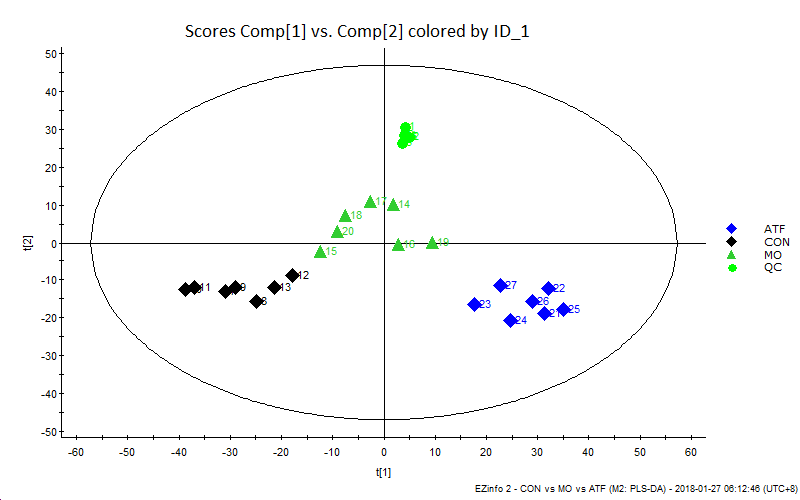I. CON vs hypo-MO *vs* hypo-ATF in positive ion mode | 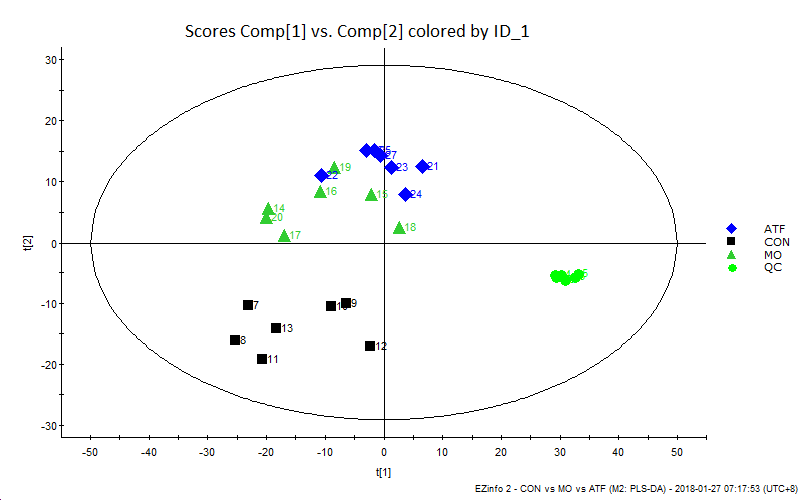 J. CON vs hypo-MO *vs* hypo-ATF in negative ion mode |

**Figures S18 PLS-DA scores plot of the UHPLC/TOF-MS spectral from MO and AMR and its fraction group in hypothyroidism**

| 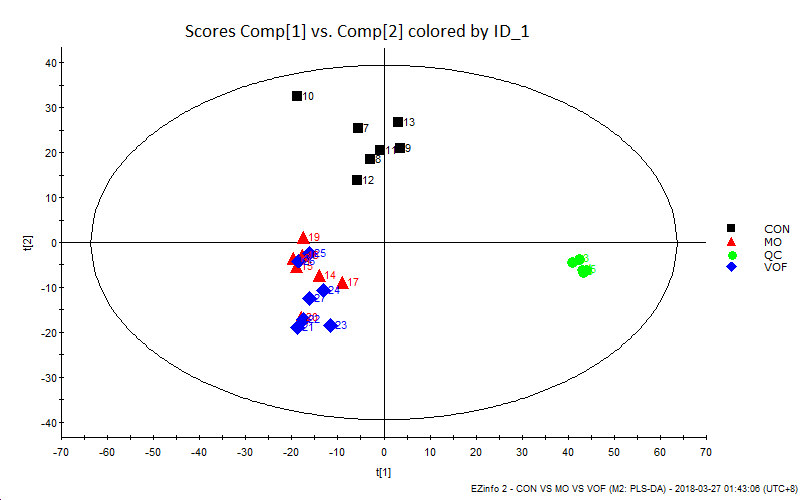 A. CON vs hype-MO *vs* hype-VOF in positive ion mode | 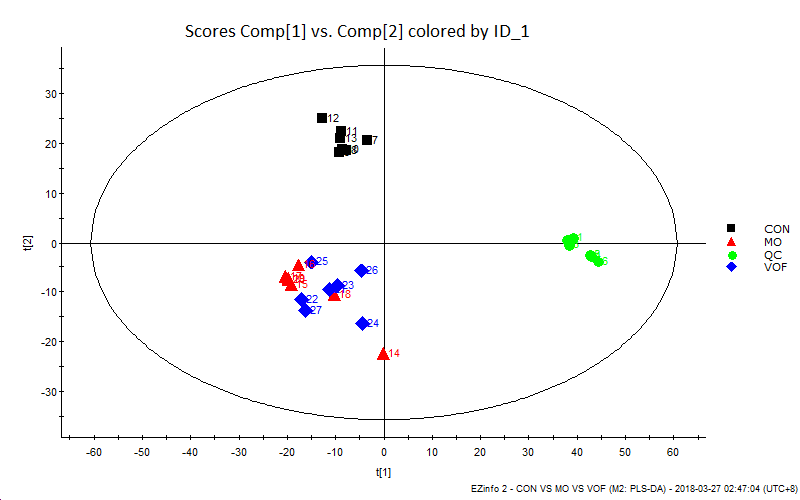 B. CON vs hype-MO *vs* hype-VOF in negative ion mode |
| --- | --- |
| 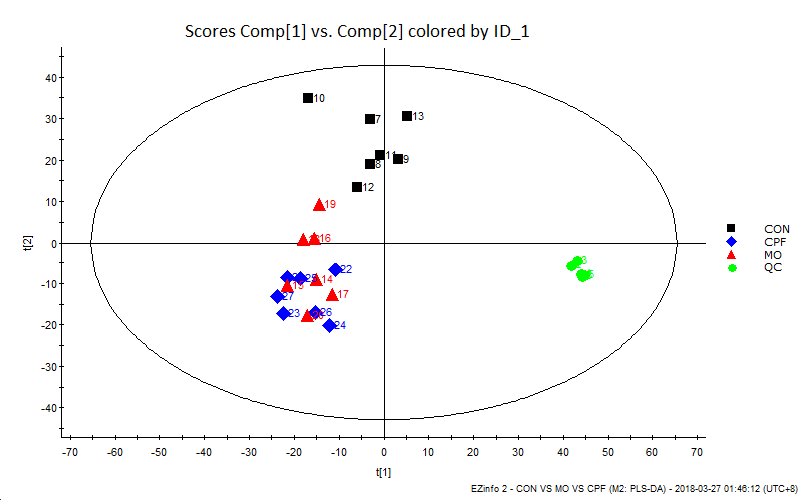 C. CON vs hype-MO *vs* hype-CPF in positive ion mode | 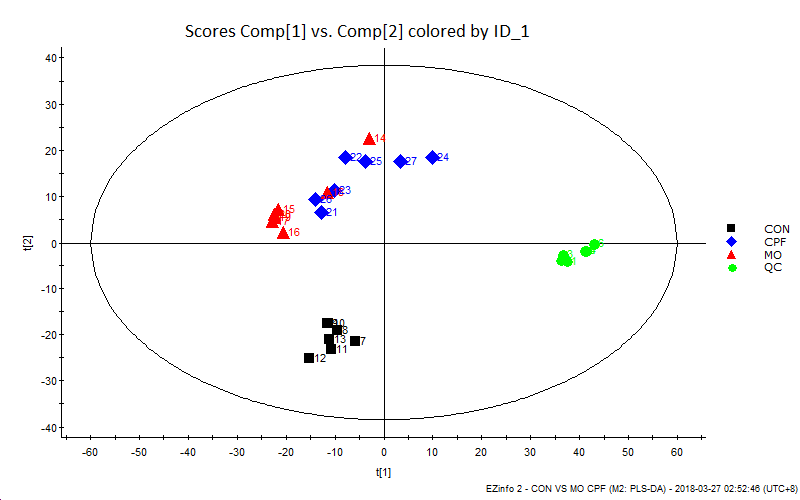 D. CON vs hype-MO *vs* hype-CPF in negative ion mode |
| 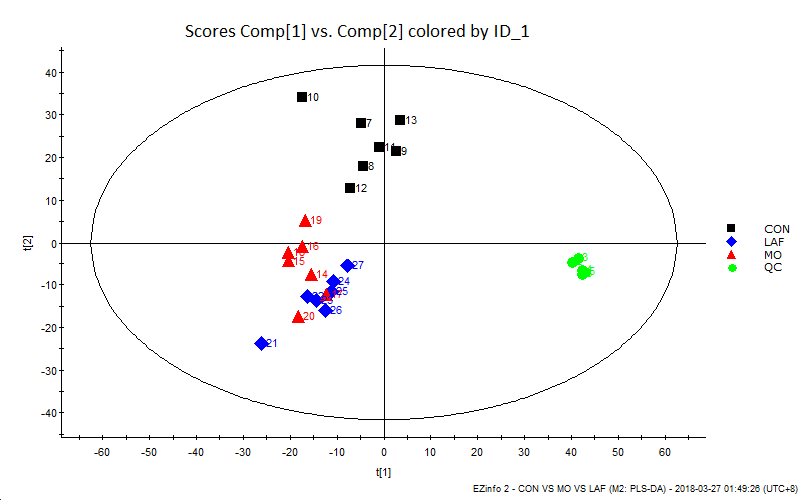 E. CON vs hype-MO *vs* hype-LAF in positive ion mode | 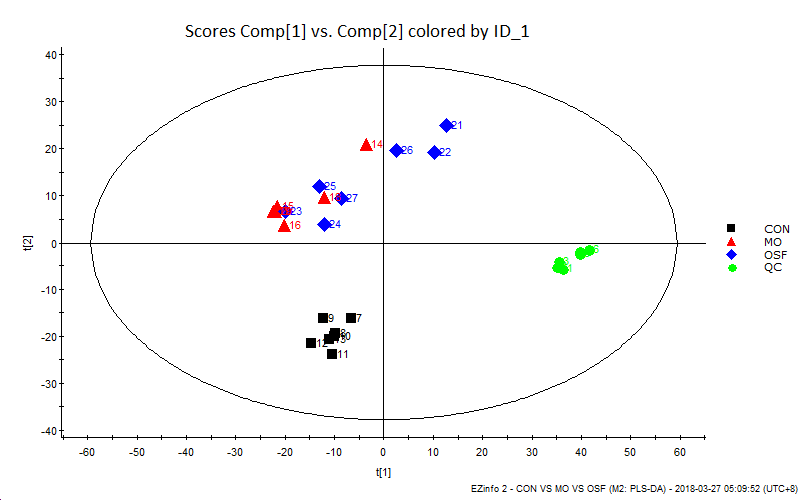 F. CON vs hype-MO *vs* hype-LAF in negative ion mode |
| 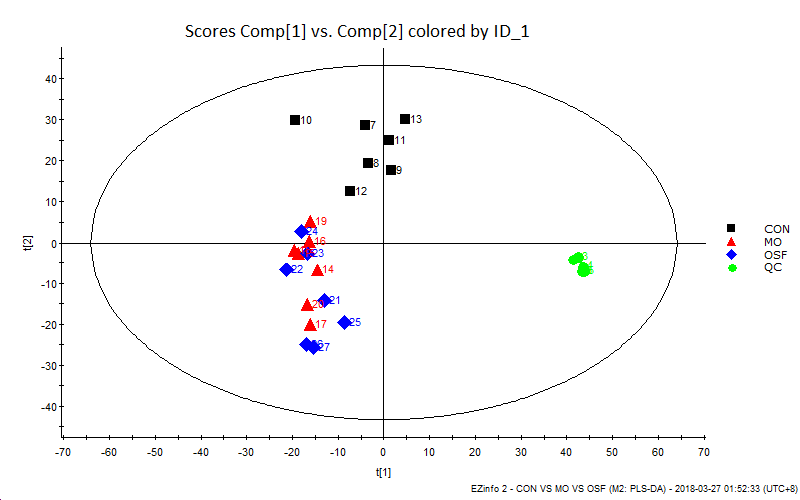 G. CON vs hype-MO *vs* hype-OSF in positive ion mode | 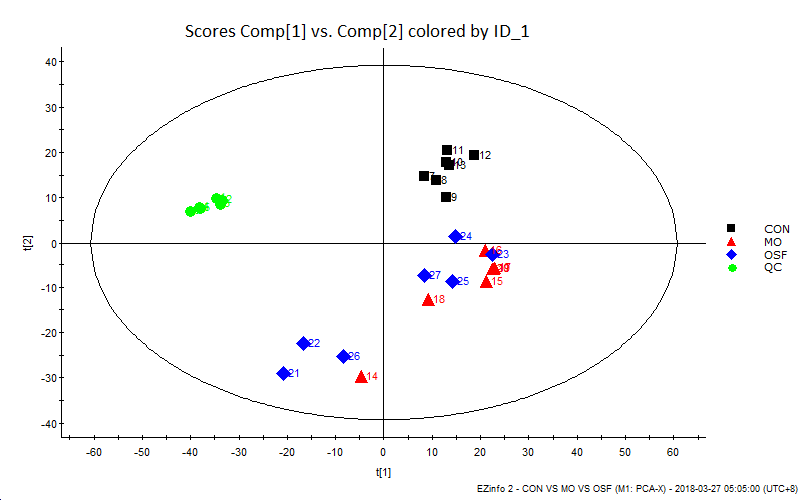 H. CON vs hype-MO *vs* hype-OSF in negative ion mode |
| 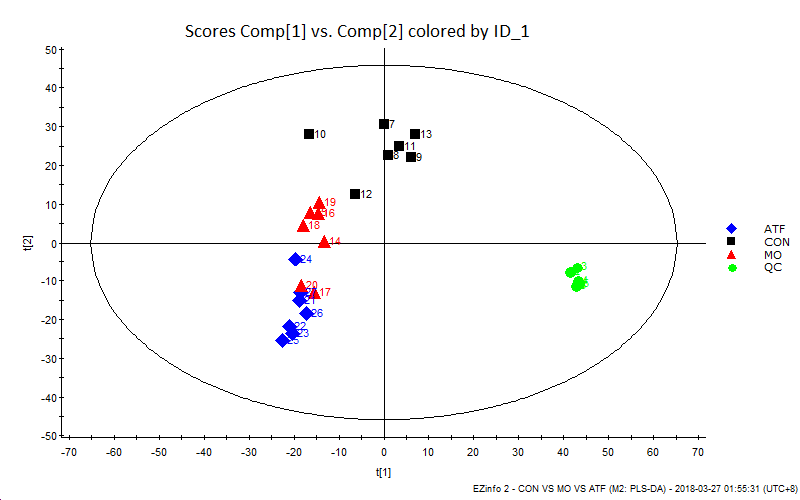 I. CON vs hype-MO *vs* hype-ATF in positive ion mode | 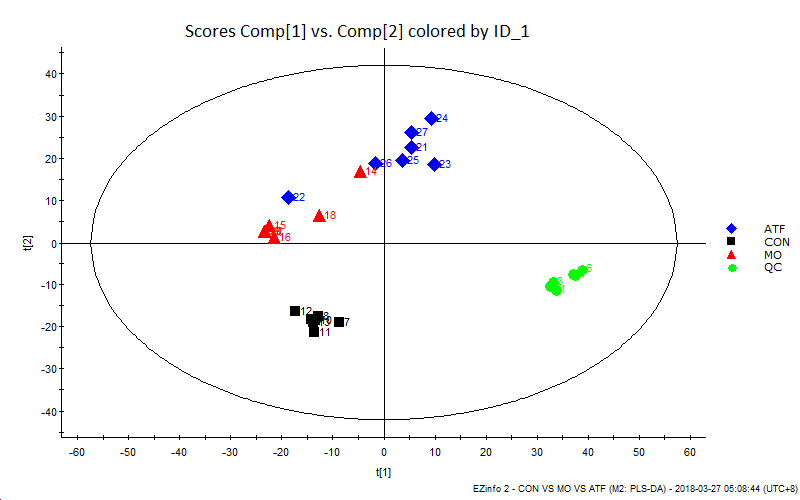 J. CON vs hype-MO *vs* hype-ATF in negative ion mode |

**Figures S19 PLS-DA scores plot of the UHPLC/TOF-MS spectral from MO and AMR and its fraction group in hyperthyroidism.**
