# Supplementary material for: Application of Proteomics and Metabonomics to Reveal the Molecular Basis of Atractylodis Macrocephalae Rhizome for Ameliorating Hypothyroidism Instead of Hyperthyroidism
Source: Front Pharmacol. 2021 Apr 20;12:664319. doi: 10.3389/fphar.2021.664319 (PMC8095350; doi:10.3389/fphar.2021.664319)
Supplement: Supplementary file 2 [file DataSheet2.docx]

Tables S1 The structure of 18 compounds in AMR

| NO. | Components | Structure |
| --- | --- | --- |
| 1 | Atractylone |  |
| 2 | Juniper camphor |  |
| 3 | AtractylenolideⅢ |  |
| 4 | Taraxeryl acetate |  |
| 5 | (4E, 6E, 12E)-tetradeca-4, 6, 12-trien-8, 10-diyne-1, 3, 14-triol |  |
| 6 | 3β-acetoxy-atractylenolide Ⅰ |  |
| 7 | Stigmasterol |  |
| 8 | Atractylenolide Ⅰ |  |
| 9 | Isoatractylenolide Ⅰ |  |
| 10 | Sitosterin |  |
| 11 | Atractylenolide Ⅱ |  |
| 12 | Dibutyl phthalate |  |
| 13 | Isoatractylenolide Ⅰ |  |
| 14 | Atractyloside A |  |
| 15 | Caprolactam |  |
| 16 | 5-Hydroxymethyl furfural ether |  |
| 17 | (4E, 6E, 12E)-3, 14-dihydroxytetradeca-4, 6, 12-trien-8, 10-diyn-1-yl acetate |  |
| 18 | 5-Hydroxymethyl furfural |  |

Tables S2 Potential targets of 18 components in AMR

| DRD1 | CES2 | PTGS1 | GABRA5 | CXCR2 | MAPK10 | CA2 |
| --- | --- | --- | --- | --- | --- | --- |
| CHRM3 | PTPRF | PLAU | HSP90AB1 | CTSK | AURKA | CA1 |
| F2 | PTPN2 | RORC | MAP2 | CTSS | VCP | CNR2 |
| CHRM1 | PLA2G1B | ESR2 | CHRNA2 | CTSL | PDE5A | CPL1 |
| ADRB1 | ACP1 | RORA | PIK3CG | CTSB | HDAC6 | cobT |
| SCN5A | AKR1B10 | SERPINA6 | KCNH2 | FAAH | MTNR1A | FUCA1 |
| NOS3 | SHBG | VDR | PRKCA | MGLL | MTNR1B | GBA |
| CHRM4 | HMGCR | NR1H2 | PON1 | MEN1 | GRM4 |  |
| PDE3A | PTPN6 | DHCR7 | JUN | ADORA2B | HTR7 |  |
| HTR2A | ICMT | GLRA1 | TGFB1 | GRM5 | TNFRSF1A |  |
| SLC6A2 | GRIA2 | G6PD | PREP | GPR55 | EPHX2 |  |
| ADRA1A | TNF | PTGER1 | PDCD4 | S1PR3 | EPHX1 |  |
| CHRM2 | RPS6KA5 | PTGER2 | CDC25C | MAPK14 | DPP7 |  |
| ADRA1B | CDC25A | PTGES | RASGRP3 | RPS6KA2 | HSD17B7 |  |
| SLC6A3 | CDC25B | PPARA | CHRM5 | MAPK11 | DPP4 |  |
| ADRB2 | PLA2G2A | PPARD | MPO | PGGT1B | DPP8 |  |
| OPRM1 | TTL | NOS2 | SRD5A1 | PDE2A | DPP9 |  |
| CHRNA7 | PGR | FDFT1 | TYMS | ADAM17 | DNM1 |  |
| PTGS2 | SIGMAR1 | PPARG | ADH1A | KCNA5 | CHRNA4 |  |
| GABRA2 | PARP1 | VEGFA | PTPRC | HTR2B | ADH1B |  |
| RXRA | DCTPP1 | IL6 | HSD11B2 | ADRA2B | HDAC3 |  |
| GABRB3 | TAS2R31 | IL1B | AHR | TACR2 | HDAC2 |  |
| GABRA1 | MDM2 | PGF | FNTA | DRD3 | ITGA2B |  |
| GABRA6 | FADS1 | TBXAS1 | NR3C1 | OPRD1 | NCOR2 |  |
| CYP19A1 | CDK2 | STAT3 | GSK3B | ADRB3 | RNPEP |  |
| SLC6A4 | P2RX7 | CYP11B1 | bla | CYP2D6 | DNPEP |  |
| UGT2B7 | PTK2B | CYP11B2 | HTR6 | CYP2C9 | HDAC1 |  |
| AR | TTR | PRKDC | TRPM8 | CYP3A4 | HDAC11 |  |
| PTPN1 | HMOX1 | KIF11 | MIF | GABRA3 | HDAC10 |  |
| NR1I3 | TRPV3 | CA3 | TSPO | GALR3 | NAAA |  |
| ATP12A | ADRA2C | CA12 | GRM2 | TMIGD3 | CHRNA3 |  |
| ACHE | SCN9A | CA14 | RGS4 | FABP1 | PAOX |  |
| ESR1 | ADH1C | CA9 | ALDH1A1 | RGS8 | HDAC8 |  |
| NR1H3 | AKR1B1 | CA13 | ALDH3A1 | CCNE2 | PLA2G2C |  |
| BCHE | ADRA2A | CA5B | TNKS2 | CES1 | PLA2G10 |  |
| CYP2C19 | MAOA | CA5A | PLG | TRPC6 | CNR1 |  |
| SREBF2 | MAOB | XPO1 | CTRC | TRPC3 | PTGER4 |  |
| NPC1L1 | CTRB1 | FLT3 | CMA1 | ADORA2A | PTGER3 |  |
| CYP51A1 | IGHG1 | BAX | CTSG | CASP7 | SRD5A2 |  |
| SQLE | LTA4H | BCL2 | ADORA1 | DYRK1B | MME |  |
| CYP17A1 | NR3C2 | CASP3 | PDE10A | TLR9 | REN |  |
| HSD11B1 | PRKACA | CASP8 | HCRTR2 | CLK3 | HSD17B3 |  |
| POLB | NCOA1 | CASP9 | HCRTR1 | ALDH2 | ADH7 |  |
| PDE4D | NCOA2 | camC | PPP1CA | MAPK8 | PLA2G4A |  |

Tables S3 Potential targets of hypothyroidism and hyperthyroidism

| Hypothyroidism | | | | Hyperthyroidism | | |
| --- | --- | --- | --- | --- | --- | --- |
| ABCC6 | DCAF17 | GNE | NKX2-1 | SKI | AKT1 | UFD1 |
| ACP5 | DCLRE1C | GP1BB | NKX2-5 | SLC16A2 | ARVCF | TPO |
| ADA | DDOST | GPR161 | NODAL | SLC25A4 | BTNL2 | THRA |
| AIP | DEAF1 | GTF2I | NPHS1 | SLC26A4 | COMT | SOD2 |
| AIRE | DISP1 | GTF2IRD1 | NSD1 | SLC30A10 | COX1 | CAT |
| AKT1 | DLL1 | HBB | NSUN2 | SLC5A5 | COX2 | PON1 |
| ALMS1 | DMXL2 | HESX1 | OTX2 | SOX3 | COX3 | GPX1 |
| ALX4 | DNAH1 | HIRA | PAX8 | SRY | GNAS | GSR |
| APC | DUOX2 | HLA-DRB1 | PCSK1 | STAT1 | GP1BB | SOD1 |
| APOE | DUOXA2 | HNF1B | PHF21A | STAT3 | HIRA | AQP1 |
| ARNT2 | EFEMP2 | HNF4A | PIEZO1 | STEAP3 | HLA-DRB1 | PTGS2 |
| ARVCF | EIF2AK3 | HPD | PIK3CA | STUB1 | JMJD1C | PTPN22 |
| ATP7B | ELN | HSD17B3 | PMM2 | SUFU | MSTO1 | IFIH1 |
| BAZ1B | ENPP1 | IGSF1 | POLG | TANGO2 | ND1 | CARTPT |
| BCHE | EXOSC2 | IL2RA | POLG2 | TBL2 | ND4 | IL6 |
| BCOR | EXT2 | IL2RG | POMC | TBX1 | ND5 | SCD |
| BMP4 | EZH2 | IL7R | POU1F1 | TDGF1 | ND6 | B3GNT2 |
| BRAF | FAM111A | IQSEC2 | PPP1R15B | TF | PIK3CA | IL6R |
| BTNL2 | FANCI | IRX5 | PRDM16 | TG | POLG | MTHFR |
| BUB1 | FBLN5 | IYD | PROKR2 | TGIF1 | POLG2 | ARID5B |
| BUB1B | FGF8 | JMJD1C | PROP1 | THRA | PTEN | CACNA1S |
| BUB3 | FGFR1 | KAT6B | PTCH1 | THRB | RREB1 | CTLA4 |
| CACNA1C | FLCN | KCNAB2 | PTEN | TPO | RRM2B | FCRL3 |
| CDH23 | FLII | KCNJ10 | PTH | TRH | SEC24C | GC |
| CDON | FMR1 | KIAA0556 | PTH1R | TRHR | SLC25A4 | RNASET2 |
| CEP57 | FOXE1 | KISS1R | PTRH2 | TRIP13 | SUGCT | TNFRSF8 |
| CHD7 | FOXH1 | LEP | RAB40AL | TRNF | TBX1 | TNFSF8 |
| CLIP2 | FOXI1 | LEPR | RAG1 | TRNH | THRB | DIO1 |
| COL4A5 | FOXP3 | LHX3 | RAG2 | TRNL1 | TRNF |  |
| COMT | FUCA1 | LHX4 | RAI1 | TRNQ | TRNH |  |
| COX1 | GABRD | LIFR | RERE | TRNS1 | TRNL1 |  |
| COX2 | GAS1 | LIG4 | RFC2 | TRNS2 | TRNQ |  |
| COX3 | GATA1 | LIMK1 | RMRP | TRNW | TRNS1 |  |
| CP | GATA3 | LRBA | ROBO1 | TSC1 | TRNS2 |  |
| CTNNB1 | GCH1 | LRP4 | RREB1 | TSC2 | TRNW |  |
| CTNS | GCM2 | MARS | RRM2B | TSHB | TSHR |  |
| DACT1 | GLI2 | MCM8 | SAA1 | TSHR | TWNK |  |
| TWNK | GLI3 | MEN1 | SALL1 |  |  |  |
| UBR1 | GLIS3 | MLXIPL | SCN4A |  |  |  |
| UFD1 | GNAS | ND1 | SEC24C |  |  |  |
| WDR11 | SEMA3E | ND4 |  |  |  |  |
| WFS1 | SGPL1 | ND5 |  |  |  |  |
| XRCC4 | SHH | ND6 |  |  |  |  |
| ZIC2 | SIX3 | NIN |  |  |  |  |

Table S4 Direct targets of hypothyroidism and hyperthyroidism treatment with AMR

| Fractions | Main components | Hypothyroidism | | Hyperthyroidism | |
| --- | --- | --- | --- | --- | --- |
|  |  | Numbers | Targets | Numbers | Targets |
| VOF | attractylone | 1 | ADRB2 | 1 | ADRB2 |
| LAF | juniper camphor | 3 | ESR1, AR, RXRA | 4 | RXRA, PTGS2, ESR1, PTPN6 |
|  | atractylenolide Ⅲ | 5 | CDK2, RPS6KA5, MDM2, PARP1, AR | 1 | PARP1 |
|  | stigmasterol | 9 | ESR1, NCOA2, PPARG, ADRB2, VDR, PRKACA, AR, NCOA1, RXRA | 7 | ADRB2, RXRA, NCOA1, PTGS2, ESR1, AKR1B1, PTPN6 |
|  | atractylenolide Ⅰ | 4 | STAT3, PRKDC, XPO1, AR | 2 | PRKDC, STAT3 |
|  | sitosterol | 7 | JUN, ESR1, NCOA2, ADRB2, VDR, PRKACA, AR | 5 | ADRB2, BCL2, PTGS2, ESR1, PTPN6 |
|  | atractylenolide II | 6 | RPS6KA5, PARP1, PPARG, PRKDC, XPO1, AR | 2 | PARP1, PRKDC |
|  | DBP | 7 | NCOA2, ADRB2, NR3C1, VCP, GSK3B, PRKACA, RXRA | 6 | ADRB2, RXRA, HDAC6, PTGS2, AURKA, VCP |
| ATF | hexanolactam | 7 | HDAC2, HDAC3, NCOR2, PARP1, NR3C1, AR, HDAC1 | 6 | HDAC2, HDAC3, HDAC1, PARP1, NCOR2, HDAC6 |
| OSF | 5-Hydroxymethylfurfural | 0 | - | 1 | PTGS2 |

Tables S5 73 significantly pathways of anti-hypothyroidism function of AMR

| Category | Term | | Count | % | PValue |
| --- | --- | --- | --- | --- | --- |
| KEGG_PATHWAY | hsa05203 | Viral carcinogenesis | 45 | 0.141363 | 1.24E-29 |
| KEGG_PATHWAY | hsa05034 | Alcoholism | 36 | 0.11309 | 2.87E-22 |
| KEGG_PATHWAY | hsa05322 | Systemic lupus erythematosus | 30 | 0.094242 | 1.11E-19 |
| KEGG_PATHWAY | hsa04919 | Thyroid hormone signaling pathway | 28 | 0.087959 | 2.36E-19 |
| KEGG_PATHWAY | hsa05202 | Transcriptional misregulation in cancer | 28 | 0.087959 | 5.94E-15 |
| KEGG_PATHWAY | hsa04110 | Cell cycle | 23 | 0.072252 | 3.32E-13 |
| KEGG_PATHWAY | hsa05215 | Prostate cancer | 19 | 0.059686 | 4.28E-12 |
| KEGG_PATHWAY | hsa05200 | Pathways in cancer | 37 | 0.116232 | 7.94E-12 |
| KEGG_PATHWAY | hsa05161 | Hepatitis B | 22 | 0.069111 | 6.80E-11 |
| KEGG_PATHWAY | hsa05220 | Chronic myeloid leukemia | 15 | 0.047121 | 2.34E-09 |
| KEGG_PATHWAY | hsa04068 | FoxO signaling pathway | 19 | 0.059686 | 5.88E-09 |
| KEGG_PATHWAY | hsa05169 | Epstein-Barr virus infection | 17 | 0.053404 | 6.10E-08 |
| KEGG_PATHWAY | hsa04915 | Estrogen signaling pathway | 15 | 0.047121 | 1.63E-07 |
| KEGG_PATHWAY | hsa03040 | Spliceosome | 17 | 0.053404 | 2.08E-07 |
| KEGG_PATHWAY | hsa04520 | Adherens junction | 12 | 0.037697 | 1.44E-06 |
| KEGG_PATHWAY | hsa04310 | Wnt signaling pathway | 16 | 0.050262 | 1.89E-06 |
| KEGG_PATHWAY | hsa05210 | Colorectal cancer | 11 | 0.034555 | 3.01E-06 |
| KEGG_PATHWAY | hsa05212 | Pancreatic cancer | 11 | 0.034555 | 4.68E-06 |
| KEGG_PATHWAY | hsa05216 | Thyroid cancer | 8 | 0.025131 | 5.95E-06 |
| KEGG_PATHWAY | hsa05162 | Measles | 15 | 0.047121 | 6.19E-06 |
| KEGG_PATHWAY | hsa05221 | Acute myeloid leukemia | 10 | 0.031414 | 9.84E-06 |
| KEGG_PATHWAY | hsa04917 | Prolactin signaling pathway | 11 | 0.034555 | 1.06E-05 |
| KEGG_PATHWAY | hsa05168 | Herpes simplex infection | 17 | 0.053404 | 1.48E-05 |
| KEGG_PATHWAY | hsa04010 | MAPK signaling pathway | 20 | 0.062828 | 2.04E-05 |
| KEGG_PATHWAY | hsa05166 | HTLV-I infection | 20 | 0.062828 | 2.16E-05 |
| KEGG_PATHWAY | hsa04114 | Oocyte meiosis | 13 | 0.040838 | 2.22E-05 |
| KEGG_PATHWAY | hsa05131 | Shigellosis | 10 | 0.031414 | 3.00E-05 |
| KEGG_PATHWAY | hsa05211 | Renal cell carcinoma | 10 | 0.031414 | 3.86E-05 |
| KEGG_PATHWAY | hsa05213 | Endometrial cancer | 9 | 0.028273 | 4.33E-05 |
| KEGG_PATHWAY | hsa04550 | Signaling pathways regulating pluripotency of stem cells | 14 | 0.04398 | 5.15E-05 |
| KEGG_PATHWAY | hsa05219 | Bladder cancer | 8 | 0.025131 | 6.62E-05 |
| KEGG_PATHWAY | hsa05164 | Influenza A | 15 | 0.047121 | 1.27E-04 |
| KEGG_PATHWAY | hsa05205 | Proteoglycans in cancer | 16 | 0.050262 | 1.61E-04 |
| KEGG_PATHWAY | hsa04120 | Ubiquitin mediated proteolysis | 13 | 0.040838 | 1.76E-04 |
| KEGG_PATHWAY | hsa04922 | Glucagon signaling pathway | 11 | 0.034555 | 1.93E-04 |
| KEGG_PATHWAY | hsa04722 | Neurotrophin signaling pathway | 12 | 0.037697 | 2.21E-04 |
| KEGG_PATHWAY | hsa05222 | Small cell lung cancer | 10 | 0.031414 | 2.84E-04 |
| KEGG_PATHWAY | hsa04662 | B cell receptor signaling pathway | 9 | 0.028273 | 3.33E-04 |
| KEGG_PATHWAY | hsa04012 | ErbB signaling pathway | 10 | 0.031414 | 3.39E-04 |
| KEGG_PATHWAY | hsa04668 | TNF signaling pathway | 11 | 0.034555 | 3.66E-04 |
| KEGG_PATHWAY | hsa04390 | Hippo signaling pathway | 13 | 0.040838 | 4.36E-04 |
| KEGG_PATHWAY | hsa04151 | PI3K-Akt signaling pathway | 21 | 0.065969 | 4.47E-04 |
| KEGG_PATHWAY | hsa05223 | Non-small cell lung cancer | 8 | 0.025131 | 4.95E-04 |
| KEGG_PATHWAY | hsa04066 | HIF-1 signaling pathway | 10 | 0.031414 | 7.04E-04 |
| KEGG_PATHWAY | hsa05206 | MicroRNAs in cancer | 18 | 0.056545 | 9.21E-04 |
| KEGG_PATHWAY | hsa04660 | T cell receptor signaling pathway | 10 | 0.031414 | 9.47E-04 |
| KEGG_PATHWAY | hsa04710 | Circadian rhythm | 6 | 0.018848 | 9.99E-04 |
| KEGG_PATHWAY | hsa05214 | Glioma | 8 | 0.025131 | 0.001228 |
| KEGG_PATHWAY | hsa04330 | Notch signaling pathway | 7 | 0.02199 | 0.001258 |
| KEGG_PATHWAY | hsa05130 | Pathogenic Escherichia coli infection | 7 | 0.02199 | 0.001733 |
| KEGG_PATHWAY | hsa04380 | Osteoclast differentiation | 11 | 0.034555 | 0.001778 |
| KEGG_PATHWAY | hsa05218 | Melanoma | 8 | 0.025131 | 0.002064 |
| KEGG_PATHWAY | hsa04510 | Focal adhesion | 14 | 0.04398 | 0.002191 |
| KEGG_PATHWAY | hsa04062 | Chemokine signaling pathway | 13 | 0.040838 | 0.00267 |
| KEGG_PATHWAY | hsa05100 | Bacterial invasion of epithelial cells | 8 | 0.025131 | 0.003535 |
| KEGG_PATHWAY | hsa05160 | Hepatitis C | 10 | 0.031414 | 0.006631 |
| KEGG_PATHWAY | hsa05142 | Chagas disease (American trypanosomiasis) | 8 | 0.025131 | 0.016422 |
| KEGG_PATHWAY | hsa04350 | TGF-beta signaling pathway | 7 | 0.02199 | 0.0197 |
| KEGG_PATHWAY | hsa04931 | Insulin resistance | 8 | 0.025131 | 0.019821 |
| KEGG_PATHWAY | hsa04210 | Apoptosis | 6 | 0.018848 | 0.020249 |
| KEGG_PATHWAY | hsa05145 | Toxoplasmosis | 8 | 0.025131 | 0.021693 |
| KEGG_PATHWAY | hsa05230 | Central carbon metabolism in cancer | 6 | 0.018848 | 0.022916 |
| KEGG_PATHWAY | hsa04910 | Insulin signaling pathway | 9 | 0.028273 | 0.024109 |
| KEGG_PATHWAY | hsa05031 | Amphetamine addiction | 6 | 0.018848 | 0.025802 |
| KEGG_PATHWAY | hsa04115 | p53 signaling pathway | 6 | 0.018848 | 0.027328 |
| KEGG_PATHWAY | hsa04024 | cAMP signaling pathway | 11 | 0.034555 | 0.029238 |
| KEGG_PATHWAY | hsa04340 | Hedgehog signaling pathway | 4 | 0.012566 | 0.03025 |
| KEGG_PATHWAY | hsa04920 | Adipocytokine signaling pathway | 6 | 0.018848 | 0.032247 |
| KEGG_PATHWAY | hsa05030 | Cocaine addiction | 5 | 0.015707 | 0.035285 |
| KEGG_PATHWAY | hsa04152 | AMPK signaling pathway | 8 | 0.025131 | 0.03691 |
| KEGG_PATHWAY | hsa04916 | Melanogenesis | 7 | 0.02199 | 0.041599 |
| KEGG_PATHWAY | hsa03450 | Non-homologous end-joining | 3 | 0.009424 | 0.041757 |

Tables S6 70 significantly pathways of anti-hyperthyroidism function of AMR

| Category | Term | | Count | % | PValue |
| --- | --- | --- | --- | --- | --- |
| KEGG_PATHWAY | hsa05200 | Pathways in cancer | 34 | 0.109783662 | 6.04E-13 |
| KEGG_PATHWAY | hsa05203 | Viral carcinogenesis | 25 | 0.080723281 | 1.21E-12 |
| KEGG_PATHWAY | hsa05161 | Hepatitis B | 19 | 0.061349693 | 3.89E-10 |
| KEGG_PATHWAY | hsa05220 | Chronic myeloid leukemia | 14 | 0.045205037 | 1.14E-09 |
| KEGG_PATHWAY | hsa04919 | Thyroid hormone signaling pathway | 16 | 0.0516629 | 6.26E-09 |
| KEGG_PATHWAY | hsa05169 | Epstein-Barr virus infection | 16 | 0.0516629 | 1.43E-08 |
| KEGG_PATHWAY | hsa05212 | Pancreatic cancer | 11 | 0.035518243 | 4.98E-07 |
| KEGG_PATHWAY | hsa04110 | Cell cycle | 14 | 0.045205037 | 9.11E-07 |
| KEGG_PATHWAY | hsa04012 | ErbB signaling pathway | 12 | 0.038747175 | 1.01E-06 |
| KEGG_PATHWAY | hsa05215 | Prostate cancer | 12 | 0.038747175 | 1.13E-06 |
| KEGG_PATHWAY | hsa04917 | Prolactin signaling pathway | 11 | 0.035518243 | 1.16E-06 |
| KEGG_PATHWAY | hsa05221 | Acute myeloid leukemia | 10 | 0.032289312 | 1.30E-06 |
| KEGG_PATHWAY | hsa05205 | Proteoglycans in cancer | 17 | 0.054891831 | 1.91E-06 |
| KEGG_PATHWAY | hsa04066 | HIF-1 signaling pathway | 12 | 0.038747175 | 2.72E-06 |
| KEGG_PATHWAY | hsa05211 | Renal cell carcinoma | 10 | 0.032289312 | 5.36E-06 |
| KEGG_PATHWAY | hsa05223 | Non-small cell lung cancer | 9 | 0.029060381 | 1.28E-05 |
| KEGG_PATHWAY | hsa05219 | Bladder cancer | 8 | 0.02583145 | 1.36E-05 |
| KEGG_PATHWAY | hsa04915 | Estrogen signaling pathway | 11 | 0.035518243 | 2.44E-05 |
| KEGG_PATHWAY | hsa05210 | Colorectal cancer | 9 | 0.029060381 | 2.76E-05 |
| KEGG_PATHWAY | hsa05166 | HTLV-I infection | 17 | 0.054891831 | 4.01E-05 |
| KEGG_PATHWAY | hsa05222 | Small cell lung cancer | 10 | 0.032289312 | 4.33E-05 |
| KEGG_PATHWAY | hsa04151 | PI3K-Akt signaling pathway | 20 | 0.064578624 | 4.73E-05 |
| KEGG_PATHWAY | hsa04510 | Focal adhesion | 15 | 0.048433968 | 5.51E-05 |
| KEGG_PATHWAY | hsa04662 | B cell receptor signaling pathway | 9 | 0.029060381 | 6.06E-05 |
| KEGG_PATHWAY | hsa05160 | Hepatitis C | 12 | 0.038747175 | 6.22E-05 |
| KEGG_PATHWAY | hsa05213 | Endometrial cancer | 8 | 0.02583145 | 6.75E-05 |
| KEGG_PATHWAY | hsa04520 | Adherens junction | 9 | 0.029060381 | 7.46E-05 |
| KEGG_PATHWAY | hsa01130 | Biosynthesis of antibiotics | 15 | 0.048433968 | 7.53E-05 |
| KEGG_PATHWAY | hsa04141 | Protein processing in endoplasmic reticulum | 13 | 0.041976106 | 1.27E-04 |
| KEGG_PATHWAY | hsa04630 | Jak-STAT signaling pathway | 12 | 0.038747175 | 1.36E-04 |
| KEGG_PATHWAY | hsa05216 | Thyroid cancer | 6 | 0.019373587 | 2.35E-04 |
| KEGG_PATHWAY | hsa05230 | Central carbon metabolism in cancer | 8 | 0.02583145 | 2.57E-04 |
| KEGG_PATHWAY | hsa04380 | Osteoclast differentiation | 11 | 0.035518243 | 2.63E-04 |
| KEGG_PATHWAY | hsa05214 | Glioma | 8 | 0.02583145 | 2.83E-04 |
| KEGG_PATHWAY | hsa05162 | Measles | 11 | 0.035518243 | 2.97E-04 |
| KEGG_PATHWAY | hsa04068 | FoxO signaling pathway | 11 | 0.035518243 | 3.16E-04 |
| KEGG_PATHWAY | hsa04664 | Fc epsilon RI signaling pathway | 8 | 0.02583145 | 3.75E-04 |
| KEGG_PATHWAY | hsa01230 | Biosynthesis of amino acids | 8 | 0.02583145 | 5.34E-04 |
| KEGG_PATHWAY | hsa04722 | Neurotrophin signaling pathway | 10 | 0.032289312 | 6.10E-04 |
| KEGG_PATHWAY | hsa04650 | Natural killer cell mediated cytotoxicity | 10 | 0.032289312 | 6.88E-04 |
| KEGG_PATHWAY | hsa04660 | T cell receptor signaling pathway | 9 | 0.029060381 | 8.04E-04 |
| KEGG_PATHWAY | hsa04666 | Fc gamma R-mediated phagocytosis | 8 | 0.02583145 | 0.001347097 |
| KEGG_PATHWAY | hsa04350 | TGF-beta signaling pathway | 8 | 0.02583145 | 0.001347097 |
| KEGG_PATHWAY | hsa01200 | Carbon metabolism | 9 | 0.029060381 | 0.001781678 |
| KEGG_PATHWAY | hsa00010 | Glycolysis / Gluconeogenesis | 7 | 0.022602519 | 0.0020601 |
| KEGG_PATHWAY | hsa04071 | Sphingolipid signaling pathway | 9 | 0.029060381 | 0.002604588 |
| KEGG_PATHWAY | hsa05218 | Melanoma | 7 | 0.022602519 | 0.002770974 |
| KEGG_PATHWAY | hsa05168 | Herpes simplex infection | 11 | 0.035518243 | 0.003429578 |
| KEGG_PATHWAY | hsa04062 | Chemokine signaling pathway | 11 | 0.035518243 | 0.003852684 |
| KEGG_PATHWAY | hsa05020 | Prion diseases | 5 | 0.016144656 | 0.004392506 |
| KEGG_PATHWAY | hsa04010 | MAPK signaling pathway | 13 | 0.041976106 | 0.00442486 |
| KEGG_PATHWAY | hsa05416 | Viral myocarditis | 6 | 0.019373587 | 0.005316631 |
| KEGG_PATHWAY | hsa04120 | Ubiquitin mediated proteolysis | 9 | 0.029060381 | 0.005845647 |
| KEGG_PATHWAY | hsa05202 | Transcriptional misregulation in cancer | 10 | 0.032289312 | 0.005944536 |
| KEGG_PATHWAY | hsa04550 | Signaling pathways regulating pluripotency of stem cells | 9 | 0.029060381 | 0.006645438 |
| KEGG_PATHWAY | hsa04064 | NF-kappa B signaling pathway | 7 | 0.022602519 | 0.007545583 |
| KEGG_PATHWAY | hsa04540 | Gap junction | 7 | 0.022602519 | 0.007968347 |
| KEGG_PATHWAY | hsa05206 | MicroRNAs in cancer | 13 | 0.041976106 | 0.011380758 |
| KEGG_PATHWAY | hsa05140 | Leishmaniasis | 6 | 0.019373587 | 0.013259649 |
| KEGG_PATHWAY | hsa05231 | Choline metabolism in cancer | 7 | 0.022602519 | 0.015122692 |
| KEGG_PATHWAY | hsa05014 | Amyotrophic lateral sclerosis (ALS) | 5 | 0.016144656 | 0.017089168 |
| KEGG_PATHWAY | hsa05142 | Chagas disease (American trypanosomiasis) | 7 | 0.022602519 | 0.017256943 |
| KEGG_PATHWAY | hsa05100 | Bacterial invasion of epithelial cells | 6 | 0.019373587 | 0.019265308 |
| KEGG_PATHWAY | hsa04114 | Oocyte meiosis | 7 | 0.022602519 | 0.023024758 |
| KEGG_PATHWAY | hsa04810 | Regulation of actin cytoskeleton | 10 | 0.032289312 | 0.023947851 |
| KEGG_PATHWAY | hsa05152 | Tuberculosis | 9 | 0.029060381 | 0.024631827 |
| KEGG_PATHWAY | hsa03450 | Non-homologous end-joining | 3 | 0.009686794 | 0.026602915 |
| KEGG_PATHWAY | hsa04370 | VEGF signaling pathway | 5 | 0.016144656 | 0.032791989 |
| KEGG_PATHWAY | hsa04210 | Apoptosis | 5 | 0.016144656 | 0.034529857 |
| KEGG_PATHWAY | hsa00270 | Cysteine and methionine metabolism | 4 | 0.012915725 | 0.039268248 |
| KEGG_PATHWAY | hsa05120 | Epithelial cell signaling in Helicobacter pylori infection | 5 | 0.016144656 | 0.044011861 |

**Tables S7 A list of proteins identified and quantified with iTRAQ technology in MO group vs CON group**

| Uniprot  Accession | Gene name | Protein name | MO：CON | |
| --- | --- | --- | --- | --- |
|  |  |  | Tendency | Fold change |
| P08683 | Cyp2c11 | Cytochrome P450 2C11 | ↓ | 0.10 |
| P05179 | Cyp2c7 | Cytochrome P450 2C7 | ↓ | 0.11 |
| P50237 | Sult1c1 | Sulfotransferase 1C1 | ↓ | 0.12 |
| P12928 | Pklr | Pyruvate kinase PKLR | ↓ | 0.16 |
| P07896 | Ehhadh | Peroxisomal bifunctional enzyme | ↓ | 0.17 |
| P31210 | Akr1d1 | 3-oxo-5-beta-steroid 4-dehydrogenase | ↓ | 0.18 |
| P25409 | Gpt | Alanine aminotransferase 1 | ↓ | 0.20 |
| P13255 | Gnmt | Glycine N-methyltransferase | ↓ | 0.21 |
| P05183 | Cyp3a2 | Cytochrome P450 3A2 | ↓ | 0.21 |
| Q8CHM7 | Hacl1 | 2-hydroxyacyl-CoA lyase 1 | ↓ | 0.21 |
| P12785 | Fasn | Fatty acid synthase | ↓ | 0.22 |
| Q4KLZ6 | Tkfc | Triokinase/FMN cyclase | ↓ | 0.23 |
| Q6MG08 | Abcf1 | ATP-binding cassette sub-family F member 1 | ↓ | 0.24 |
| Q8VHE9 | Retsat | All-trans-retinol 13,14-reductase | ↓ | 0.26 |
| P52873 | Pc | Pyruvate carboxylase, mitochondrial | ↓ | 0.27 |
| P16232 | Hsd11b1 | Corticosteroid 11-beta-dehydrogenase isozyme 1 | ↓ | 0.28 |
| P04176 | Pah | Phenylalanine-4-hydroxylase | ↓ | 0.28 |
| Q63342 | Dmgdh | Dimethylglycine dehydrogenase, mitochondrial | ↓ | 0.28 |
| Q64611 | Csad | Cysteine sulfinic acid decarboxylase | ↓ | 0.29 |
| P04762 | Cat | Catalase | ↓ | 0.32 |
| O35077 | Gpd1 | Glycerol-3-phosphate dehydrogenase [NAD(+)], cytoplasmic | ↓ | 0.33 |
| P18163 | Acsl1 | Long-chain-fatty-acid--CoA ligase 1 | ↓ | 0.34 |
| P04182 | Oat | Ornithine aminotransferase, mitochondrial | ↓ | 0.36 |
| P16638 | Acly | ATP-citrate synthase | ↓ | 0.37 |
| P13107 | Cyp2b3 | Cytochrome P450 2B3 | ↓ | 0.37 |
| O88813 | Acsl5 | Long-chain-fatty-acid--CoA ligase 5 | ↓ | 0.38 |
| Q6P6V0 | Gpi | Glucose-6-phosphate isomerase | ↓ | 0.38 |
| P38652 | Pgm1 | Phosphoglucomutase-1 | ↓ | 0.39 |
| P09811 | Pygl | Glycogen phosphorylase, liver form | ↓ | 0.41 |
| P11884 | Aldh2 | Aldehyde dehydrogenase, mitochondrial | ↓ | 0.42 |
| Q6SKG1 | Acsm3 | Acyl-coenzyme A synthetase ACSM3, mitochondrial | ↓ | 0.46 |
| P97852 | Hsd17b4 | Peroxisomal multifunctional enzyme type 2 | ↓ | 0.47 |
| P08541 | Ugt2b | UDP-glucuronosyltransferase 2B2 | ↓ | 0.48 |
| Q8VHT6 | As3mt | Arsenite methyltransferase | ↓ | 0.48 |
| P07872 | Acox1 | Peroxisomal acyl-coenzyme A oxidase 1 | ↓ | 0.49 |
| P10634 | Cyp2d26 | Cytochrome P450 2D26 | ↓ | 0.51 |
| Q07116 | Suox | Sulfite oxidase, mitochondrial | ↓ | 0.51 |
| O09171 | Bhmt | Betaine--homocysteine S-methyltransferase 1 | ↓ | 0.52 |
| P14141 | Ca3 | Carbonic anhydrase 3 | ↓ | 0.54 |
| P15149 | Cyp2a2 | Cytochrome P450 2A2 | ↓ | 0.56 |
| Q64581 | Cyp3a18 | Cytochrome P450 3A18 | ↓ | 0.57 |
| Q9Z1A6 | Hdlbp | Vigilin | ↓ | 0.58 |
| Q5I0C3 | Mccc1 | Methylcrotonoyl-CoA carboxylase subunit alpha, mitochondrial | ↓ | 0.60 |
| Q63108 | Ces1e | Carboxylesterase 1E | ↑ | 1.51 |
| P97524 | Slc27a2 | Very long-chain acyl-CoA synthetase | ↑ | 1.51 |
| Q64428 | Hadha | Trifunctional enzyme subunit alpha, mitochondrial | ↑ | 1.53 |
| P04905 | Gstm1 | Glutathione S-transferase Mu 1 | ↑ | 1.56 |
| P62271 | Rps18 | 40S ribosomal protein S18 | ↑ | 1.56 |
| P14408 | Fh | Fumarate hydratase, mitochondrial | ↑ | 1.60 |
| Q63120 | Abcc2 | Canalicular multispecific organic anion transporter 1 | ↑ | 1.61 |
| O70490 | Acsm2 | Acyl-coenzyme A synthetase ACSM2, mitochondrial | ↑ | 1.64 |
| Q6IRK9 | Cpq | Carboxypeptidase Q | ↑ | 1.67 |
| P05182 | Cyp2e1 | Cytochrome P450 2E1 | ↑ | 1.72 |
| P24368 | Ppib | Peptidyl-prolyl cis-trans isomerase B | ↑ | 1.72 |
| Q09073 | Slc25a5 | ADP/ATP translocase 2 | ↑ | 1.72 |
| P46953 | Haao | 3-hydroxyanthranilate 3,4-dioxygenase | ↑ | 1.77 |
| P23457 | Akr1c9 | 3-alpha-hydroxysteroid dehydrogenase | ↑ | 1.80 |
| P21213 | Hal | Histidine ammonia-lyase | ↑ | 1.82 |
| P22789 | St2a2 | Alcohol sulfotransferase A | ↑ | 1.87 |
| P24329 | Tst | Thiosulfate sulfurtransferase | ↑ | 1.96 |
| P04799 | Cyp1a2 | Cytochrome P450 1A2 | ↑ | 2.00 |
| P11232 | Txn | Thioredoxin | ↑ | 2.07 |
| P48500 | Tpi1 | Triosephosphate isomerase | ↑ | 2.38 |
| Q5XIH7 | Phb2 | Prohibitin-2 | ↑ | 2.58 |
| P13086 | Suclg1 | Succinate--CoA ligase [ADP/GDP-forming] subunit alpha, mitochondrial | ↑ | 2.65 |
| O70199 | Ugdh | UDP-glucose 6-dehydrogenase | ↑ | 2.70 |
| P08430 | Ugt1a6 | UDP-glucuronosyltransferase 1-6 | ↑ | 2.81 |
| P32755 | Hpd | 4-hydroxyphenylpyruvate dioxygenase | ↑ | 2.83 |
| P41562 | Idh1 | Isocitrate dehydrogenase [NADP] cytoplasmic | ↑ | 3.08 |
| P06757 | Adh1 | Alcohol dehydrogenase 1 | ↑ | 3.10 |
| P80254 | Ddt | D-dopachrome decarboxylase | ↑ | 3.34 |
| Q10758 | Krt8 | Keratin, type II cytoskeletal 8 | ↑ | 3.37 |
| Q6AYT9 | Acsm5 | Acyl-coenzyme A synthetase ACSM5, mitochondrial | ↑ | 3.91 |
| P07687 | Ephx1 | Epoxide hydrolase 1 | ↑ | 3.94 |
| P07895 | Sod2 | Superoxide dismutase [Mn], mitochondrial | ↑ | 3.94 |
| Q5BJY9 | Krt18 | Keratin, type I cytoskeletal 18 | ↑ | 4.21 |
| P51647 | Aldh1a1 | Retinal dehydrogenase 1 | ↑ | 4.61 |
| P05982 | Nqo1 | NAD(P)H dehydrogenase [quinone] 1 | ↑ | 5.40 |

**Tables S8 A list of proteins identified and quantified with iTRAQ technology in WD group vs MO group**

| Uniprot  Accession | Gene name | Protein name | WD：MO | |
| --- | --- | --- | --- | --- |
|  |  |  | Tendency | Fold change |
| Q9Z339 | Gsto1 | Glutathione S-transferase omega-1 | ↓ | 0.25 |
| Q03336 | Rgn | Regucalcin | ↓ | 0.32 |
| P80067 | Ctsc | Dipeptidyl peptidase 1 | ↓ | 0.33 |
| P80254 | Ddt | D-dopachrome decarboxylase | ↓ | 0.34 |
| Q6AYT9 | Acsm5 | Acyl-coenzyme A synthetase ACSM5, mitochondrial | ↓ | 0.39 |
| Q6IRK9 | Cpq | Carboxypeptidase Q | ↓ | 0.41 |
| P08430 | Ugt1a6 | UDP-glucuronosyltransferase 1-6 | ↓ | 0.43 |
| P04799 | Cyp1a2 | Cytochrome P450 1A2 | ↓ | 0.44 |
| P11960 | Bckdha | 2-oxoisovalerate dehydrogenase subunit alpha, mitochondrial (Fragment) | ↓ | 0.44 |
| Q68FS4 | Lap3 | Cytosol aminopeptidase | ↓ | 0.47 |
| P10860 | Glud1 | Glutamate dehydrogenase 1, mitochondrial | ↓ | 0.49 |
| P17178 | Cyp27a1 | Sterol 26-hydroxylase, mitochondrial | ↓ | 0.50 |
| P02770 | Alb | Serum albumin | ↓ | 0.51 |
| O70199 | Ugdh | UDP-glucose 6-dehydrogenase | ↓ | 0.51 |
| P13444 | Mat1a | S-adenosylmethionine synthase isoform type-1 | ↓ | 0.52 |
| P05182 | Cyp2e1 | Cytochrome P450 2E1 | ↓ | 0.57 |
| P07687 | Ephx1 | Epoxide hydrolase 1 | ↓ | 0.58 |
| P04905 | Gstm1 | Glutathione S-transferase Mu 1 | ↓ | 0.60 |
| Q66HF1 | Ndufs1 | NADH-ubiquinone oxidoreductase 75 kDa subunit, mitochondrial | ↓ | 0.61 |
| Q09073 | Slc25a5 | ADP/ATP translocase 2 | ↓ | 0.61 |
| P27867 | Sord | Sorbitol dehydrogenase | ↓ | 0.63 |
| Q9Z1A6 | Hdlbp | Vigilin | ↑ | 1.67 |
| P21533 | Rpl6 | 60S ribosomal protein L6 | ↑ | 1.74 |
| P28037 | Aldh1l1 | Cytosolic 10-formyltetrahydrofolate dehydrogenase | ↑ | 1.77 |
| Q63617 | Hyou1 | Hypoxia up-regulated protein 1 | ↑ | 1.79 |
| P10634 | Cyp2d26 | Cytochrome P450 2D26 | ↑ | 1.80 |
| P04764 | Eno1 | Alpha-enolase | ↑ | 1.82 |
| P09811 | Pygl | Glycogen phosphorylase, liver form | ↑ | 1.94 |
| P07872 | Acox1 | Peroxisomal acyl-coenzyme A oxidase 1 | ↑ | 2.19 |
| P97852 | Hsd17b4 | Peroxisomal multifunctional enzyme type 2 | ↑ | 2.31 |
| P38652 | Pgm1 | Phosphoglucomutase-1 | ↑ | 2.36 |
| P52873 | Pc | Pyruvate carboxylase, mitochondrial | ↑ | 2.42 |
| Q499N5 | Acsf2 | Acyl-CoA synthetase family member 2, mitochondrial | ↑ | 2.44 |
| Q6P6V0 | Gpi | Glucose-6-phosphate isomerase | ↑ | 2.47 |
| P16638 | Acly | ATP-citrate synthase | ↑ | 2.54 |
| O55171 | Acot2 | Acyl-coenzyme A thioesterase 2, mitochondrial | ↑ | 2.65 |
| P04762 | Cat | Catalase | ↑ | 2.75 |
| O88813 | Acsl5 | Long-chain-fatty-acid--CoA ligase 5 | ↑ | 2.83 |
| P62961 | Ybx1 | Nuclease-sensitive element-binding protein 1 | ↑ | 2.83 |
| P18163 | Acsl1 | Long-chain-fatty-acid--CoA ligase 1 | ↑ | 3.13 |
| Q63342 | Dmgdh | Dimethylglycine dehydrogenase, mitochondrial | ↑ | 3.16 |
| P13255 | Gnmt | Glycine N-methyltransferase | ↑ | 3.66 |
| P25409 | Gpt | Alanine aminotransferase 1 | ↑ | 3.80 |
| P05183 | Cyp3a2 | Cytochrome P450 3A2 | ↑ | 3.98 |
| P12928 | Pklr | Pyruvate kinase PKLR | ↑ | 3.98 |
| Q64611 | Csad | Cysteine sulfinic acid decarboxylase | ↑ | 4.21 |
| O35077 | Gpd1 | Glycerol-3-phosphate dehydrogenase [NAD(+)], cytoplasmic | ↑ | 4.49 |
| Q8CHM7 | Hacl1 | 2-hydroxyacyl-CoA lyase 1 | ↑ | 4.61 |
| P12785 | Fasn | Fatty acid synthase | ↑ | 4.92 |
| Q4KLZ6 | Tkfc | Triokinase/FMN cyclase | ↑ | 5.06 |
| P07896 | Ehhadh | Peroxisomal bifunctional enzyme | ↑ | 7.52 |
| P50237 | Sult1c1 | Sulfotransferase 1C1 | ↑ | 7.73 |

**Tables S9 A list of proteins identified and quantified with iTRAQ technology in VOF group vs MO group**

| Uniprot  Accession | Gene name | Protein name | VOF：MO | |
| --- | --- | --- | --- | --- |
|  |  |  | Tendency | Fold change |
| P50237 | Sult1c1 | Sulfotransferase 1C1 | ↑ | 9.38 |
| P05179 | Cyp2c7 | Cytochrome P450 2C7 | ↑ | 8.47 |
| P07896 | Ehhadh | Peroxisomal bifunctional enzyme | ↑ | 6.73 |
| P08683 | Cyp2c11 | Cytochrome P450 2C11 | ↑ | 6.67 |
| Q4KLZ6 | Tkfc | Triokinase/FMN cyclase | ↑ | 5.60 |
| P12928 | Pklr | Pyruvate kinase PKLR | ↑ | 5.60 |
| Q07066 | Pxmp2 | Peroxisomal membrane protein 2 | ↑ | 5.01 |
| Q6MG08 | Abcf1 | ATP-binding cassette sub-family F member 1 | ↑ | 4.97 |
| P16232 | Hsd11b1 | Corticosteroid 11-beta-dehydrogenase isozyme 1 | ↑ | 4.92 |
| Q64611 | Csad | Cysteine sulfinic acid decarboxylase | ↑ | 4.70 |
| P25409 | Gpt | Alanine aminotransferase 1 | ↑ | 4.53 |
| Q8CHM7 | Hacl1 | 2-hydroxyacyl-CoA lyase 1 | ↑ | 4.53 |
| O35077 | Gpd1 | Glycerol-3-phosphate dehydrogenase [NAD(+)], cytoplasmic | ↑ | 4.33 |
| P12785 | Fasn | Fatty acid synthase | ↑ | 4.17 |
| Q8VHE9 | Retsat | All-trans-retinol 13,14-reductase | ↑ | 4.02 |
| Q6P6V0 | Gpi | Glucose-6-phosphate isomerase | ↑ | 3.94 |
| Q63342 | Dmgdh | Dimethylglycine dehydrogenase, mitochondrial | ↑ | 3.87 |
| P31210 | Akr1d1 | 3-oxo-5-beta-steroid 4-dehydrogenase | ↑ | 3.87 |
| P18163 | Acsl1 | Long-chain-fatty-acid--CoA ligase 1 | ↑ | 3.80 |
| P05183 | Cyp3a2 | Cytochrome P450 3A2 | ↑ | 3.80 |
| P38652 | Pgm1 | Phosphoglucomutase-1 | ↑ | 3.34 |
| P13255 | Gnmt | Glycine N-methyltransferase | ↑ | 3.31 |
| P04762 | Cat | Catalase | ↑ | 3.19 |
| P52873 | Pc | Pyruvate carboxylase, mitochondrial | ↑ | 2.94 |
| Q75Q40 | Tomm40 | Mitochondrial import receptor subunit TOM40 homolog | ↑ | 2.78 |
| P48679 | Lmna | Prelamin-A/C | ↑ | 2.70 |
| P53987 | Slc16a1 | Monocarboxylate transporter 1 | ↑ | 2.49 |
| Q7TQM4 | Soat2 | Sterol O-acyltransferase 2 | ↑ | 2.27 |
| P12938 | Cyp2d3 | Cytochrome P450 2D3 | ↑ | 2.21 |
| P07872 | Acox1 | Peroxisomal acyl-coenzyme A oxidase 1 | ↑ | 2.19 |
| Q9Z2Z8 | Dhcr7 | 7-dehydrocholesterol reductase | ↑ | 2.13 |
| P09811 | Pygl | Glycogen phosphorylase, liver form | ↑ | 2.07 |
| P97852 | Hsd17b4 | Peroxisomal multifunctional enzyme type 2 | ↑ | 2.03 |
| Q9Z2M4 | Decr2 | Peroxisomal 2,4-dienoyl-CoA reductase | ↑ | 1.84 |
| P05178 | Cyp2c6 | Cytochrome P450 2C6 | ↑ | 1.80 |
| P18484 | Ap2a2 | AP-2 complex subunit alpha-2 | ↑ | 1.79 |
| Q68FT9 | Scly | Selenocysteine lyase | ↑ | 1.72 |
| Q9Z1A6 | Hdlbp | Vigilin | ↑ | 1.64 |
| Q5I0C3 | Mccc1 | Methylcrotonoyl-CoA carboxylase subunit alpha, mitochondrial | ↑ | 1.61 |
| P11442 | Cltc | Clathrin heavy chain 1 | ↑ | 1.54 |
| P30427 | Plec | Plectin | ↑ | 1.53 |
| P13635 | Cp | Ceruloplasmin | ↓ | 0.65 |
| P13635 | Cp | Ceruloplasmin | ↓ | 0.65 |
| P32755 | Hpd | 4-hydroxyphenylpyruvate dioxygenase | ↓ | 0.65 |
| P24368 | Ppib | Peptidyl-prolyl cis-trans isomerase B | ↓ | 0.65 |
| P04785 | Pgm1 | Protein disulfide-isomerase | ↓ | 0.64 |
| P49242 | Rps3a | 40S ribosomal protein S3a | ↓ | 0.63 |
| P10111 | Ppia | Peptidyl-prolyl cis-trans isomerase A | ↓ | 0.63 |
| P48500 | Tpi1 | Triosephosphate isomerase | ↓ | 0.62 |
| P46953 | Haao | 3-hydroxyanthranilate 3,4-dioxygenase | ↓ | 0.62 |
| Q6IRK9 | Cpq | Carboxypeptidase Q | ↓ | 0.61 |
| Q5XIH7 | Phb2 | Prohibitin-2 | ↓ | 0.61 |
| Q64428 | Hadha | Trifunctional enzyme subunit alpha, mitochondrial | ↓ | 0.59 |
| Q3T1L0 | Aldh16a1 | Aldehyde dehydrogenase family 16 member A1 | ↓ | 0.58 |
| P07687 | Ephx1 | Epoxide hydrolase 1 | ↓ | 0.57 |
| P07632 | Sod1 | Superoxide dismutase [Cu-Zn] | ↓ | 0.56 |
| Q68FS4 | Lap3 | Cytosol aminopeptidase | ↓ | 0.55 |
| P08430 | Ugt1a6 | UDP-glucuronosyltransferase 1-6 | ↓ | 0.54 |
| Q66HD0 | Hsp90b1 | Endoplasmin | ↓ | 0.53 |
| O09171 | Bhmt | Betaine--homocysteine S-methyltransferase 1 | ↓ | 0.53 |
| O70199 | Ugdh | UDP-glucose 6-dehydrogenase | ↓ | 0.51 |
| Q66HF1 | Ndufs1 | NADH-ubiquinone oxidoreductase 75 kDa subunit, mitochondrial | ↓ | 0.49 |
| P09367 | Sds | L-serine dehydratase/L-threonine deaminase | ↓ | 0.49 |
| P17178 | Cyp27a1 | Sterol 26-hydroxylase, mitochondrial | ↓ | 0.48 |
| P09034 | Ass1 | Argininosuccinate synthase | ↓ | 0.48 |
| P06757 | Adh1 | Alcohol dehydrogenase 1 | ↓ | 0.47 |
| P51647 | Aldh1a1 | Retinal dehydrogenase 1 | ↓ | 0.46 |
| P04797 | Gapdh | Glyceraldehyde-3-phosphate dehydrogenase | ↓ | 0.44 |
| P80067 | Ctsc | Dipeptidyl peptidase 1 | ↓ | 0.43 |
| P04799 | Cyp1a2 | Cytochrome P450 1A2 | ↓ | 0.38 |
| P05982 | Nqo1 | NAD(P)H dehydrogenase [quinone] 1 | ↓ | 0.29 |
| P01946 | Hba1 | Hemoglobin subunit alpha-1/2 | ↓ | 0.12 |
| P02770 | Alb | Serum albumin | ↓ | 0.08 |

**Tables S10 A list of proteins identified and quantified with iTRAQ technology in CPF group vs MO group**

| Uniprot  Accession | Gene name | Protein name | CPF：MO | |
| --- | --- | --- | --- | --- |
|  |  |  | Fold change | Tendency |
| P50237 | Sult1c1 | Sulfotransferase 1C1 | 7.80 | ↑ |
| P05179 | Cyp2c7 | Cytochrome P450 2C7 | 7.52 | ↑ |
| P08683 | Cyp2c11 | Cytochrome P450 2C11 | 6.08 | ↑ |
| Q4KLZ6 | Tkfc | Triokinase/FMN cyclase | 5.11 | ↑ |
| P12928 | Pklr | Pyruvate kinase PKLR | 4.49 | ↑ |
| P07896 | Ehhadh | Peroxisomal bifunctional enzyme | 4.33 | ↑ |
| P25409 | Gpt | Alanine aminotransferase 1 | 4.17 | ↑ |
| Q8CHM7 | Hacl1 | 2-hydroxyacyl-CoA lyase 1 | 4.02 | ↑ |
| P12785 | Fasn | Fatty acid synthase | 3.80 | ↑ |
| Q64611 | Csad | Cysteine sulfinic acid decarboxylase | 3.80 | ↑ |
| Q63342 | Dmgdh | Dimethylglycine dehydrogenase, mitochondrial | 3.66 | ↑ |
| P18163 | Acsl1 | Long-chain-fatty-acid--CoA ligase 1 | 3.53 | ↑ |
| O88813 | Acsl5 | Long-chain-fatty-acid--CoA ligase 5 | 3.28 | ↑ |
| P05183 | Cyp3a2 | Cytochrome P450 3A2 | 3.28 | ↑ |
| Q9Z2S9 | Flot2 | Flotillin-2 | 3.22 | ↑ |
| P52873 | Pc | Pyruvate carboxylase, mitochondrial | 3.08 | ↑ |
| P04182 | Oat | Ornithine aminotransferase, mitochondrial | 2.96 | ↑ |
| P13255 | Gnmt | Glycine N-methyltransferase | 2.91 | ↑ |
| Q03346 | Pmpcb | Mitochondrial-processing peptidase subunit beta | 2.78 | ↑ |
| P16638 | Acly | ATP-citrate synthase | 2.70 | ↑ |
| P53987 | Slc16a1 | Monocarboxylate transporter 1 | 2.65 | ↑ |
| P10868 | Gamt | Guanidinoacetate N-methyltransferase | 2.63 | ↑ |
| P16086 | Sptan1 | Spectrin alpha chain, non-erythrocytic 1 | 2.40 | ↑ |
| P11884 | Aldh2 | Aldehyde dehydrogenase, mitochondrial | 2.17 | ↑ |
| P04764 | Eno1 | Alpha-enolase | 2.17 | ↑ |
| Q6SKG1 | Acsm3 | Acyl-coenzyme A synthetase ACSM3, mitochondrial | 2.13 | ↑ |
| Q5BJY9 | Krt18 | Keratin, type I cytoskeletal 18 | 2.07 | ↑ |
| Q63617 | Hyou1 | Hypoxia up-regulated protein 1 | 1.75 | ↑ |
| Q9Z1A6 | Hdlbp | Vigilin | 1.74 | ↑ |
| P11915 | Scp2 | Non-specific lipid-transfer protein | 1.72 | ↑ |
| Q66X93 | Snd1 | Staphylococcal nuclease domain-containing protein 1 | 1.67 | ↑ |
| Q10758 | Krt8 | Keratin, type II cytoskeletal 8 | 1.67 | ↑ |
| P48679 | Lmna | Prelamin-A/C | 1.64 | ↑ |
| P30427 | Plec | Plectin | 1.53 | ↑ |
| P27653 | Mthfd1 | C-1-tetrahydrofolate synthase, cytoplasmic | 1.51 | ↑ |
| P19945 | Rplp0 | 60S acidic ribosomal protein P0 | 0.66 | ↓ |
| Q66HD0 | Hsp90b1 | Endoplasmin | 0.65 | ↓ |
| P49242 | Rps3a | 40S ribosomal protein S3a | 0.64 | ↓ |
| Q8VIF7 | Selenbp1 | Selenium-binding protein 1 | 0.63 | ↓ |
| P18886 | Cpt2 | Carnitine O-palmitoyltransferase 2, mitochondrial | 0.63 | ↓ |
| P10860 | Glud1 | Glutamate dehydrogenase 1, mitochondrial | 0.61 | ↓ |
| P55159 | Pon1 | Serum paraoxonase/arylesterase 1 | 0.61 | ↓ |
| P63245 | Rack1 | Receptor of activated protein C kinase 1 | 0.61 | ↓ |
| P80254 | Ddt | D-dopachrome decarboxylase | 0.59 | ↓ |
| Q5BK63 | Ndufa9 | NADH dehydrogenase [ubiquinone] 1 alpha subcomplex subunit 9, mitochondrial | 0.57 | ↓ |
| Q64428 | Hadha | Trifunctional enzyme subunit alpha, mitochondrial | 0.55 | ↓ |
| Q5XIH7 | Phb2 | Prohibitin-2 | 0.55 | ↓ |
| Q3MIF4 | Xylb | Xylulose kinase | 0.54 | ↓ |
| P13635 | Cp | Ceruloplasmin | 0.54 | ↓ |
| P13086 | Suclg1 | Succinate--CoA ligase [ADP/GDP-forming] subunit alpha, mitochondrial | 0.52 | ↓ |
| Q66HF1 | Ndufs1 | NADH-ubiquinone oxidoreductase 75 kDa subunit, mitochondrial | 0.50 | ↓ |
| P56571 | P1 SV=2 | ES1 protein homolog, mitochondrial | 0.50 | ↓ |
| Q09073 | Slc25a5 | ADP/ATP translocase 2 | 0.50 | ↓ |
| P07687 | Ephx1 | Epoxide hydrolase 1 | 0.49 | ↓ |
| P04785 | P4hb | Protein disulfide-isomerase | 0.49 | ↓ |
| P04799 | Cyp1a2 | Cytochrome P450 1A2 | 0.49 | ↓ |
| P17425 | Hmgcs1 | Hydroxymethylglutaryl-CoA synthase, cytoplasmic | 0.47 | ↓ |
| P11232 | Txn | Thioredoxin | 0.47 | ↓ |
| P01946 | Hba1 | Hemoglobin subunit alpha-1/2 | 0.45 | ↓ |
| P04905 | Gstm1 | Glutathione S-transferase Mu 1 | 0.45 | ↓ |
| O70199 | Ugdh | UDP-glucose 6-dehydrogenase | 0.45 | ↓ |
| P08430 | Ugt1a6 | UDP-glucuronosyltransferase 1-6 | 0.44 | ↓ |
| Q9Z339 | Gsto1 | Glutathione S-transferase omega-1 | 0.38 | ↓ |
| Q63120 | Abcc2 | Canalicular multispecific organic anion transporter 1 | 0.36 | ↓ |
| P00507 | Got2 | Aspartate aminotransferase, mitochondrial | 0.36 | ↓ |
| P16303 | Ces1d | Carboxylesterase 1D | 0.35 | ↓ |
| P12346 | Tf | Serotransferrin | 0.29 | ↓ |
| P80067 | Ctsc | Dipeptidyl peptidase 1 | 0.26 | ↓ |
| B0BNA5 | Cotl1 | Coactosin-like protein | 0.24 | ↓ |
| P51647 | Aldh1a1 | Retinal dehydrogenase 1 | 0.21 | ↓ |
| P13437 | Acaa2 | 3-ketoacyl-CoA thiolase, mitochondrial | 0.18 | ↓ |
| P02770 | Alb | Serum albumin | 0.14 | ↓ |

**Tables S11 A list of proteins identified and quantified with iTRAQ technology in LAF group *vs* MO group**

| Uniprot  Accession | Gene name | Protein name | LAF：MO | |
| --- | --- | --- | --- | --- |
|  |  |  | Fold change | Tendency |
| P07896 | Ehhadh | Peroxisomal bifunctional enzyme | ↑ | 7.94 |
| P04167 | Cyp2b2 | Cytochrome P450 2B2 | ↑ | 6.19 |
| P05179 | Cyp2c7 | Cytochrome P450 2C7 | ↑ | 6.03 |
| Q6MG08 | Abcf1 | ATP-binding cassette sub-family F member 1 | ↑ | 5.30 |
| Q02974 | Khk | Ketohexokinase | ↑ | 5.06 |
| Q63448 | Acox3 | Peroxisomal acyl-coenzyme A oxidase 3 | ↑ | 4.83 |
| P12785 | Fasn | Fatty acid synthase | ↑ | 4.70 |
| Q4KLZ6 | Tkfc | Triokinase/FMN cyclase | ↑ | 4.33 |
| Q8VHE9 | Retsat | All-trans-retinol 13,14-reductase | ↑ | 4.06 |
| P25409 | Gpt | Alanine aminotransferase 1 | ↑ | 3.94 |
| P16232 | Hsd11b1 | Corticosteroid 11-beta-dehydrogenase isozyme 1 | ↑ | 3.77 |
| O88813 | Acsl5 | Long-chain-fatty-acid--CoA ligase 5 | ↑ | 3.70 |
| Q8CHM7 | Hacl1 | 2-hydroxyacyl-CoA lyase 1 | ↑ | 3.60 |
| P07872 | Acox1 | Peroxisomal acyl-coenzyme A oxidase 1 | ↑ | 3.19 |
| P05178 | Cyp2c6 | Cytochrome P450 2C6 | ↑ | 3.19 |
| P13601 | Aldh1a7 | Aldehyde dehydrogenase, cytosolic 1 | ↑ | 3.13 |
| P18163 | Acsl1 | Long-chain-fatty-acid--CoA ligase 1 | ↑ | 2.83 |
| P16638 | Acly | ATP-citrate synthase | ↑ | 2.83 |
| P97852 | Hsd17b4 | Peroxisomal multifunctional enzyme type 2 | ↑ | 2.61 |
| P62961 | Ybx1 | Nuclease-sensitive element-binding protein 1 | ↑ | 2.58 |
| Q62651 | Ech1 | Delta(3,5)-Delta(2,4)-dienoyl-CoA isomerase, mitochondrial | ↑ | 2.44 |
| Q6P6V0 | Gpi | Glucose-6-phosphate isomerase | ↑ | 2.38 |
| P04764 | Eno1 | Alpha-enolase | ↑ | 2.33 |
| P22791 | Hmgcs2 | Hydroxymethylglutaryl-CoA synthase, mitochondrial | ↑ | 2.33 |
| P11915 | Scp2 | Non-specific lipid-transfer protein | ↑ | 2.27 |
| P38650 | Dync1h1 | Cytoplasmic dynein 1 heavy chain 1 | ↑ | 2.23 |
| P50137 | Tkt | Transketolase | ↑ | 2.19 |
| P05369 | Fdps | Farnesyl pyrophosphate synthase | ↑ | 2.19 |
| Q9EPH8 | Pabpc1 | Polyadenylate-binding protein 1 | ↑ | 2.07 |
| Q5BJY9 | Krt18 | Keratin, type I cytoskeletal 18 | ↑ | 1.96 |
| P09875 | Ugt2b1 | UDP-glucuronosyltransferase 2B1 | ↑ | 1.96 |
| P13444 | Mat1a | S-adenosylmethionine synthase isoform type-1 | ↑ | 1.87 |
| P11711 | Cyp2a1 | Cytochrome P450 2A1 | ↑ | 1.85 |
| P38659 | Pdia4 | Protein disulfide-isomerase A4 | ↑ | 1.82 |
| P24268 | Ctsd | Cathepsin D | ↑ | 1.80 |
| P13255 | Gnmt | Glycine N-methyltransferase | ↑ | 1.79 |
| P05197 | Eef2 | Elongation factor 2 | ↑ | 1.77 |
| Q8K4C0 | Fmo5 | Dimethylaniline monooxygenase [N-oxide-forming] 5 | ↑ | 1.60 |
| O88989 | Mdh1 | Malate dehydrogenase, cytoplasmic | ↑ | 1.60 |
| Q9Z1A6 | Hdlbp | Vigilin | ↑ | 1.58 |
| P32198 | Cpt1a | Carnitine O-palmitoyltransferase 1, liver isoform | ↑ | 1.51 |
| Q9ES38 | Slc27a5 | Bile acyl-CoA synthetase | ↑ | 1.51 |
| P10860 | Glud1 | Glutamate dehydrogenase 1, mitochondrial | ↓ | 0.65 |
| Q7TP48 | Apmap | Adipocyte plasma membrane-associated protein | ↓ | 0.64 |
| P19511 | Atp5f1 | ATP synthase F(0) complex subunit B1, mitochondrial | ↓ | 0.63 |
| P05182 | Cyp2e1 | Cytochrome P450 2E1 | ↓ | 0.61 |
| P63039 | Hspd1 | 60 kDa heat shock protein, mitochondrial | ↓ | 0.60 |
| Q07523 | Hao2 | Hydroxyacid oxidase 2 | ↓ | 0.60 |
| Q66HF1 | Ndufs1 | NADH-ubiquinone oxidoreductase 75 kDa subunit, mitochondrial | ↓ | 0.59 |
| Q68FY0 | Uqcrc1 | Cytochrome b-c1 complex subunit 1, mitochondrial | ↓ | 0.58 |
| P08430 | Ugt1a6 | UDP-glucuronosyltransferase 1-6 | ↓ | 0.56 |
| P11960 | Bckdha | 2-oxoisovalerate dehydrogenase subunit alpha, mitochondrial (Fragment) | ↓ | 0.54 |
| P20814 | Cyp2c13 | Cytochrome P450 2C13, male-specific | ↓ | 0.52 |
| P00481 | Otc | Ornithine carbamoyltransferase, mitochondrial | ↓ | 0.51 |
| Q6IRK9 | Cpq | Carboxypeptidase Q | ↓ | 0.51 |
| P07379 | Pck1 | Phosphoenolpyruvate carboxykinase, cytosolic [GTP] | ↓ | 0.50 |
| Q64428 | Hadha | Trifunctional enzyme subunit alpha, mitochondrial | ↓ | 0.48 |
| P04799 | Cyp1a2 | Cytochrome P450 1A2 | ↓ | 0.47 |
| P41562 | Idh1 | Isocitrate dehydrogenase [NADP] cytoplasmic | ↓ | 0.47 |
| P21213 | Hal | Histidine ammonia-lyase | ↓ | 0.45 |
| P29266 | Hibadh | 3-hydroxyisobutyrate dehydrogenase, mitochondrial | ↓ | 0.44 |
| Q3T1L0 | Aldh16a1 | Aldehyde dehydrogenase family 16 member A1 | ↓ | 0.42 |
| Q64573 | P2 SV=2 | Liver carboxylesterase 4 | ↓ | 0.39 |
| P11884 | Aldh2 | Aldehyde dehydrogenase, mitochondrial | ↓ | 0.39 |
| P12007 | Ivd | Isovaleryl-CoA dehydrogenase, mitochondrial | ↓ | 0.31 |
| P29147 | Bdh1 | D-beta-hydroxybutyrate dehydrogenase, mitochondrial | ↓ | 0.21 |
| P14141 | Ca3 | Carbonic anhydrase 3 | ↓ | 0.12 |

**Tables S12 A list of proteins identified and quantified with iTRAQ technology in OSF group *vs* MO group**

| Uniprot  Accession | Gene name | Protein name | OSF：MO | |
| --- | --- | --- | --- | --- |
|  |  |  | Fold change | Tendency |
| Q4KLZ6 | Tkfc | Triokinase/FMN cyclase | ↑ | 7.24 |
| P07896 | Ehhadh | Peroxisomal bifunctional enzyme | ↑ | 6.67 |
| Q6MG08 | Abcf1 | ATP-binding cassette sub-family F member 1 | ↑ | 6.31 |
| P12928 | Pklr | Pyruvate kinase PKLR | ↑ | 5.60 |
| P50237 | Sult1c1 | Sulfotransferase 1C1 | ↑ | 5.55 |
| P12785 | Fasn | Fatty acid synthase | ↑ | 5.45 |
| P01048 | Map1 | T-kininogen 1 | ↑ | 4.66 |
| P25409 | Gpt | Alanine aminotransferase 1 | ↑ | 4.49 |
| P16638 | Acly | ATP-citrate synthase | ↑ | 4.13 |
| O35077 | Gpd1 | Glycerol-3-phosphate dehydrogenase [NAD(+)], cytoplasmic | ↑ | 3.87 |
| P04642 | Ldha | L-lactate dehydrogenase A chain | ↑ | 3.70 |
| Q63610 | Tpm3 | Tropomyosin alpha-3 chain | ↑ | 3.70 |
| P31210 | Akr1d1 | 3-oxo-5-beta-steroid 4-dehydrogenase | ↑ | 3.47 |
| O88813 | Acsl5 | Long-chain-fatty-acid--CoA ligase 5 | ↑ | 3.22 |
| P13255 | Gnmt | Glycine N-methyltransferase | ↑ | 3.10 |
| P05197 | Eef2 | Elongation factor 2 | ↑ | 2.99 |
| P04182 | Oat | Ornithine aminotransferase, mitochondrial | ↑ | 2.99 |
| P18163 | Acsl1 | Long-chain-fatty-acid--CoA ligase 1 | ↑ | 2.91 |
| P97852 | Hsd17b4 | Peroxisomal multifunctional enzyme type 2 | ↑ | 2.73 |
| P09006 | Serpina3n | Serine protease inhibitor A3N | ↑ | 2.70 |
| Q6P6V0 | Gpi | Glucose-6-phosphate isomerase | ↑ | 2.70 |
| P38659 | Pdia4 | Protein disulfide-isomerase A4 | ↑ | 2.61 |
| Q499N5 | Acsf2 | Acyl-CoA synthetase family member 2, mitochondrial | ↑ | 2.40 |
| Q9Z2Z8 | Dhcr7 | 7-dehydrocholesterol reductase | ↑ | 2.31 |
| Q64611 | Csad | Cysteine sulfinic acid decarboxylase | ↑ | 2.31 |
| P09811 | Pygl | Glycogen phosphorylase, liver form | ↑ | 2.29 |
| P48679 | Lmna | Prelamin-A/C | ↑ | 2.27 |
| P09034 | Ass1 | Argininosuccinate synthase | ↑ | 2.23 |
| P17475 | Serpina1 | Alpha-1-antiproteinase | ↑ | 2.07 |
| Q9Z1A6 | Hdlbp | Vigilin | ↑ | 2.05 |
| P38652 | Pgm1 | Phosphoglucomutase-1 | ↑ | 2.03 |
| Q641Y0 | Ddost | Dolichyl-diphosphooligosaccharide--protein glycosyltransferase 48 kDa subunit | ↑ | 1.94 |
| P52873 | Pc | Pyruvate carboxylase, mitochondrial | ↑ | 1.92 |
| P62630 | Eef1a1 | Elongation factor 1-alpha 1 | ↑ | 1.87 |
| P01026 | C3 | Complement C3 | ↑ | 1.79 |
| P06399 | Fga | Fibrinogen alpha chain | ↑ | 1.79 |
| Q63617 | Hyou1 | Hypoxia up-regulated protein 1 | ↑ | 1.77 |
| Q5I0C3 | Mccc1 | Methylcrotonoyl-CoA carboxylase subunit alpha, mitochondrial | ↑ | 1.75 |
| P11915 | Scp2 | Non-specific lipid-transfer protein | ↑ | 1.74 |
| P20059 | Hpx | Hemopexin | ↑ | 1.67 |
| P06761 | Hspa5 | 78 kDa glucose-regulated protein | ↑ | 1.67 |
| P16086 | Sptan1 | Spectrin alpha chain, non-erythrocytic 1 | ↑ | 1.66 |
| P05369 | Fdps | Farnesyl pyrophosphate synthase | ↑ | 1.61 |
| P62718 | Rpl18a | 60S ribosomal protein L18a | ↓ | 0.63 |
| P17425 | Hmgcs1 | Hydroxymethylglutaryl-CoA synthase, cytoplasmic | ↓ | 0.63 |
| Q4QQT4 | Ppp2r1b | Serine/threonine-protein phosphatase 2A 65 kDa regulatory subunit A beta isoform | ↓ | 0.62 |
| Q63108 | Ces1e | Carboxylesterase 1E | ↓ | 0.58 |
| P02770 | Alb | Serum albumin | ↓ | 0.57 |
| P13086 | Suclg1 | Succinate--CoA ligase [ADP/GDP-forming] subunit alpha, mitochondrial | ↓ | 0.56 |
| P08541 | Ugt2b | UDP-glucuronosyltransferase 2B2 | ↓ | 0.52 |
| Q09073 | Slc25a5 | ADP/ATP translocase 2 | ↓ | 0.52 |
| P11951 | Cox6c2 | Cytochrome c oxidase subunit 6C-2 | ↓ | 0.51 |
| Q63413 | Ddx39b | Spliceosome RNA helicase Ddx39b | ↓ | 0.51 |
| P05982 | Nqo1 | NAD(P)H dehydrogenase [quinone] 1 | ↓ | 0.49 |
| P18886 | Cpt2 | Carnitine O-palmitoyltransferase 2, mitochondrial | ↓ | 0.49 |
| D4AAT7 | Naxd | ATP-dependent (S)-NAD(P)H-hydrate dehydratase | ↓ | 0.48 |
| Q2TA68 | Opa1 | Dynamin-like 120 kDa protein, mitochondrial | ↓ | 0.48 |
| P27867 | Sord | Sorbitol dehydrogenase | ↓ | 0.47 |
| Q8VIF7 | Selenbp1 | Selenium-binding protein 1 | ↓ | 0.46 |
| P08430 | Ugt1a6 | UDP-glucuronosyltransferase 1-6 | ↓ | 0.46 |
| O88994 | P2017/3/2 | Mitochondrial amidoxime reducing component 2 | ↓ | 0.45 |
| P10860 | Glud1 | Glutamate dehydrogenase 1, mitochondrial | ↓ | 0.44 |
| P10111 | Ppia | Peptidyl-prolyl cis-trans isomerase A | ↓ | 0.44 |
| Q794E4 | Hnrnpf | Heterogeneous nuclear ribonucleoprotein F | ↓ | 0.44 |
| Q5PQT3 | Glyat | Glycine N-acyltransferase | ↓ | 0.42 |
| Q66HF1 | Ndufs1 | NADH-ubiquinone oxidoreductase 75 kDa subunit, mitochondrial | ↓ | 0.42 |
| P08010 | Gstm2 | Glutathione S-transferase Mu 2 | ↓ | 0.42 |
| Q6IRK9 | Cpq | Carboxypeptidase Q | ↓ | 0.41 |
| Q64428 | Hadha | Trifunctional enzyme subunit alpha, mitochondrial | ↓ | 0.40 |
| P13676 | Apeh | Acylamino-acid-releasing enzyme | ↓ | 0.39 |
| P04799 | Cyp1a2 | Cytochrome P450 1A2 | ↓ | 0.38 |
| P23457 | Akr1c9 | 3-alpha-hydroxysteroid dehydrogenase | ↓ | 0.38 |
| P80254 | Ddt | D-dopachrome decarboxylase | ↓ | 0.36 |
| Q63120 | Abcc2 | Canalicular multispecific organic anion transporter 1 | ↓ | 0.35 |
| O70199 | Ugdh | UDP-glucose 6-dehydrogenase | ↓ | 0.34 |
| P17178 | Cyp27a1 | Sterol 26-hydroxylase, mitochondrial | ↓ | 0.32 |
| P07687 | Ephx1 | Epoxide hydrolase 1 | ↓ | 0.31 |
| P97524 | Slc27a2 | Very long-chain acyl-CoA synthetase | ↓ | 0.31 |
| Q64573 | P2 SV=2 | Liver carboxylesterase 4 | ↓ | 0.29 |
| P80067 | Ctsc | Dipeptidyl peptidase 1 | ↓ | 0.28 |
| P51647 | Aldh1a1 | Retinal dehydrogenase 1 | ↓ | 0.26 |
| P04916 | Rbp4 | Retinol-binding protein 4 | ↓ | 0.22 |
| P04905 | Gstm1 | Glutathione S-transferase Mu 1 | ↓ | 0.20 |
| P11232 | Txn | Thioredoxin | ↓ | 0.17 |
| P05182 | Cyp2e1 | Cytochrome P450 2E1 | ↓ | 0.17 |
| P07379 | Pck1 | Phosphoenolpyruvate carboxykinase, cytosolic [GTP] | ↓ | 0.17 |
| P50169 | Rdh3 | Retinol dehydrogenase 3 | ↓ | 0.04 |

**Tables S13 list of proteins identified and quantified with iTRAQ technology in ATF group *vs* MO group**

| Uniprot  Accession | Gene name | Protein name | ATF：MO | |
| --- | --- | --- | --- | --- |
|  |  |  | Fold change | Tendency |
| P50237 | Sult1c1 | Sulfotransferase 1C1 | ↑ | 6.98 |
| P07896 | Ehhadh | Peroxisomal bifunctional enzyme | ↑ | 5.97 |
| P12785 | Fasn | Fatty acid synthase | ↑ | 5.55 |
| P12928 | Pklr | Pyruvate kinase PKLR | ↑ | 4.09 |
| P25409 | Gpt | Alanine aminotransferase 1 | ↑ | 3.25 |
| P13255 | Gnmt | Glycine N-methyltransferase | ↑ | 3.19 |
| P08683 | Cyp2c11 | Cytochrome P450 2C11 | ↑ | 3.05 |
| Q63342 | Dmgdh | Dimethylglycine dehydrogenase, mitochondrial | ↑ | 2.70 |
| P16638 | Acly | ATP-citrate synthase | ↑ | 2.56 |
| O88813 | Acsl5 | Long-chain-fatty-acid--CoA ligase 5 | ↑ | 2.56 |
| Q4KLZ6 | Tkfc | Triokinase/FMN cyclase | ↑ | 2.49 |
| P16232 | Hsd11b1 | Corticosteroid 11-beta-dehydrogenase isozyme 1 | ↑ | 2.47 |
| P18163 | Acsl1 | Long-chain-fatty-acid--CoA ligase 1 | ↑ | 2.40 |
| Q499N5 | Acsf2 | Acyl-CoA synthetase family member 2, mitochondrial | ↑ | 2.33 |
| P32089 | Slc25a1 | Tricarboxylate transport protein, mitochondrial | ↑ | 2.01 |
| Q03626 | Mug1 | Murinoglobulin-1 | ↑ | 1.92 |
| P52873 | Pc | Pyruvate carboxylase, mitochondrial | ↑ | 1.91 |
| P08541 | Ugt2b | UDP-glucuronosyltransferase 2B2 | ↑ | 1.63 |
| P05197 | Eef2 | Elongation factor 2 | ↑ | 1.61 |
| P12346 | Tf | Serotransferrin | ↑ | 1.61 |
| P05369 | Fdps | Farnesyl pyrophosphate synthase | ↑ | 1.56 |
| Q5BJQ0 | Coq8a | Atypical kinase COQ8A, mitochondrial | ↑ | 1.54 |
| P97852 | Hsd17b4 | Peroxisomal multifunctional enzyme type 2 | ↑ | 1.51 |
| P24368 | Ppib | Peptidyl-prolyl cis-trans isomerase B | ↓ | 0.65 |
| P14141 | Ca3 | Carbonic anhydrase 3 | ↓ | 0.64 |
| Q63108 | Ces1e | Carboxylesterase 1E | ↓ | 0.58 |
| Q3B7D0 | Cpox | Oxygen-dependent coproporphyrinogen-III oxidase, mitochondrial | ↓ | 0.56 |
| Q3KR86 | Immt | MICOS complex subunit Mic60 (Fragment) | ↓ | 0.53 |
| P00884 | Aldob | Fructose-bisphosphate aldolase B | ↓ | 0.53 |
| Q66HF1 | Ndufs1 | NADH-ubiquinone oxidoreductase 75 kDa subunit, mitochondrial | ↓ | 0.52 |
| P04785 | P4hb | Protein disulfide-isomerase | ↓ | 0.52 |
| P48500 | Tpi1 | Triosephosphate isomerase | ↓ | 0.51 |
| P21213 | Hal | Histidine ammonia-lyase | ↓ | 0.50 |
| Q09073 | Slc25a5 | ADP/ATP translocase 2 | ↓ | 0.49 |
| Q63120 | Abcc2 | Canalicular multispecific organic anion transporter 1 | ↓ | 0.48 |
| P46953 | Haao | 3-hydroxyanthranilate 3,4-dioxygenase | ↓ | 0.47 |
| Q6IRK9 | Cpq | Carboxypeptidase Q | ↓ | 0.46 |
| P20280 | Rpl21 | 60S ribosomal protein L21 | ↓ | 0.46 |
| P55053 | Fabp5 | Fatty acid-binding protein, epidermal | ↓ | 0.45 |
| P11232 | Txn | Thioredoxin | ↓ | 0.45 |
| P56571 | P1 SV=2 | ES1 protein homolog, mitochondrial | ↓ | 0.44 |
| P62193 | Psmc1 | 26S protease regulatory subunit 4 | ↓ | 0.42 |
| P80254 | Ddt | D-dopachrome decarboxylase | ↓ | 0.42 |
| P16303 | Ces1d | Carboxylesterase 1D | ↓ | 0.41 |
| P08010 | Gstm2 | Glutathione S-transferase Mu 2 | ↓ | 0.41 |
| P08430 | Ugt1a6 | UDP-glucuronosyltransferase 1-6 | ↓ | 0.41 |
| Q68FS4 | Lap3 | Cytosol aminopeptidase | ↓ | 0.41 |
| P18886 | Cpt2 | Carnitine O-palmitoyltransferase 2, mitochondrial | ↓ | 0.41 |
| O70199 | Ugdh | UDP-glucose 6-dehydrogenase | ↓ | 0.40 |
| Q920J4 | Txnl1 | Thioredoxin-like protein 1 | ↓ | 0.39 |
| Q7TP48 | Apmap | Adipocyte plasma membrane-associated protein | ↓ | 0.35 |
| P04905 | Gstm1 | Glutathione S-transferase Mu 1 | ↓ | 0.33 |
| P08461 | Dlat | Dihydrolipoyllysine-residue acetyltransferase component of pyruvate dehydrogenase complex, mitochondrial | ↓ | 0.32 |
| P07632 | Sod1 | Superoxide dismutase [Cu-Zn] | ↓ | 0.31 |
| P07379 | Pc | Phosphoenolpyruvate carboxykinase, cytosolic [GTP] | ↓ | 0.27 |
| P07687 | Ephx1 | Epoxide hydrolase 1 | ↓ | 0.24 |
| B0BNA5 | Cotl1 | Coactosin-like protein | ↓ | 0.22 |
| P51647 | Aldh1a1 | Retinal dehydrogenase 1 | ↓ | 0.20 |
| P05982 | Nqo1 | NAD(P)H dehydrogenase [quinone] 1 | ↓ | 0.13 |
| P16446 | Pitpna | Phosphatidylinositol transfer protein alpha isoform | ↓ | 0.08 |
| Q5U2Y0 | Wdr45 | WD repeat domain phosphoinositide-interacting protein 4 | ↓ | 0.01 |

**Tables S14 Mass databases of 27 metabolites in hypothyroidism rats**

| NO. | RT(min) | Ion(m/z) | Ion mode | Elemental  composition | Identification | Related pathway | MO vs CON | | WD vs MO | | CPF vs MO | | VOF vs MO | | LAF vs MO | | OSF vs MO | | ATF vs MO | |
| --- | --- | --- | --- | --- | --- | --- | --- | --- | --- | --- | --- | --- | --- | --- | --- | --- | --- | --- | --- | --- |
|  |  |  |  |  |  |  | VIP | Trend | VIP | Trend | VIP | Trend | VIP | Trend | VIP | Trend | VIP | Trend | VIP | Trend |
| 1 | 0.81 | 145.0085 | [M-H]^-^ | C_5_H_6_O_5_ | Oxalacetic acid | Citrate cycle (TCA cycle) | 2.85 | ↓ | 0.27 | ↑ | 3.07 | ↑ | 0.84 | ¯ | 2.93 | ↑ | 1.04 | ¯ | 1.72 | ↑ |
| 2 | 0.90 | 115.0014 | [M-H2O-H]^-^ | C_4_H_4_O_4_ | Malic acid |  | 0.54 | ↓ | 0.19 | ↑ | 1.10 | - | 1.11 | ↑ | 0.81 | - | 0.66 | - | 0.89 | - |
| 3 | 2.41 | 180.0902 | [M+H]^+^ | C_6_H_13_NO_5_ | Glucosamine | Amino sugar and nucleotide sugar metabolism | 2.38 | ↓ | 1.56 | ¯ | 0.58 | ¯ | 0.55 | ¯ | 1.78 | ↑ | 2.61 | ↑ | 1.16 | ↑ |
| 4 | 5.76 | 143.1077 | [M+H]^+^ | C_8_H_14_O_2_ | 3-Hexenyl acetate | Fatty acid metabolism | 1.51 | ↓ | 0.74 | ¯ | 1.34 | ¯ | 0.13 | ¯ | 0.08 | ¯ | 0.68 | ¯ | 0.89 | ¯ |
| 5 | 0.77 | 119.0312 | [M+HCOOH-H]^-^ | C_3_H_6_O_2_ | 3-Hydroxypropanal |  | 1.40 | ↑ | 1.10 | ¯ | 1.15 | ↓ | 1.17 | ¯ | 0.25 | ¯ | 0.71 | ¯ | 0.19 | ¯ |
| 6 | 2.51 | 226.1083 | [M+H]^+^ | C_9_H_18_ClNO_4_ | Acetylcarnitine |  | 2.36 | ↑ | 2.32 | ↓ | 1.98 | ¯ | 1.25 | ¯ | 1.28 | ¯ | 0.65 | ↓ | 0.29 | ¯ |
| 7 | 8.08 | 361.2027 | [M-H2O+H]^+^ | C_21_H_34_O_5_ | Cortolone | Steroid synthesis biosynthesis | 1.40 | ↓ | 0.54 | ¯ | 0.20 | ¯ | 0.21 | ¯ | 0.47 | ¯ | 0.14 | ¯ | 0.27 | ¯ |
| 8 | 6.31 | 349.2396 | [M+H]^+^ | C_21_H_32_O_4_ | 3β, 17α, 21-trihydroxy-pregnenone |  | 1.02 | ↓ | 0.62 | ↑ | 0.26 | ¯ | 1.19 | ↑ | 0.81 | ¯ | 0.15 | ¯ | 0.57 | ¯ |
| 9 | 6.11 | 174.0564 | [M-H]^-^ | C_10_H_9_NO_2_ | 3-Indoleacetic Acid | Tryptophan metabolism | 1.06 | ↑ | 1.19 | ↓ | 0.51 | ¯ | 0.66 | ¯ | 0.27 | ¯ | 0.85 | ↓ | 0.82 | ↓ |
| 10 | 6.14 | 130.0642 | [M+H]^+^ | C_9_H_7_N | Isoquinoline |  | 2.12 | ↑ | 2.69 | ↓ | 0.34 | - | 2.43 | ↓ | 1.87 | ↓ | 2.98 | ↓ | 2.17 | ↓ |
| 11 | 3.02 | 210.0783 | [M+H]^+^ | C_10_H_11_NO_4_ | Hydroxyphenylacetylglycine |  | 2.88 | ↑ | 1.11 | - | 1.96 | - | 0.02 | - | 1.38 | - | 1.15 | - | 1.34 | - |
| 12 | 3.19 | 190.049 | [M+H]^+^ | C_10_H_7_NO_3_ | Kynurenic acid |  | 4.49 | ↓ | 0.55 | - | 2.83 | ↓ | 1.47 | - | 0.51 | - | 0.23 | - | 1.52 | - |
| 13 | 2.38 | 191.0818 | [M+H]^+^ | C_10_H_12_N_2_O_3_ | Kynurenine |  | 2.12 | ↓ | 3.28 | ↓ | 2.57 | ↓ | 1.85 | ↓ | 0.79 | - | 1.79 | ↓ | 2.08 | ↓ |
| 14 | 1.02 | 273.1083 | [M-H2O+H]^+^ | C_10_H_18_N_4_O_6_ | Arginosuccinic acid | Arginine and proline metabolism | 3.56 | ↑ | 1.78 | - | 2.65 | ↓ | 3.33 | ↑ | 1.89 | ↑ | 0.69 | - | 0.41 | - |
| 15 | 4.30 | 194.0833 | [M+H]^+^ | C_10_H_11_NO_3_ | Phenylacetylglycine | Phenylalanine metabolism | 6.94 | ↑ | 12.36 | - | 16.25 | - | 9.59 | - | 13.26 | - | 12.77 | - | 13.07 | - |
| 16 | 0.82 | 170.0936 | [M+H]^+^ | C_7_H_11_N_3_O_2_ | 1-methylhistidine | Histidine metabolism | 0.71 | ↓ | 0.39 | - | 0.07 | - | 0.01 | - | 0.44 | - | 1.32 | ↑ | 0.79 | - |
| 17 | 2.59 | 279.1366 | [M+H]^+^ | C_11_H_22_N_2_O_4_S | Pantetheine | - | 1.15 | ↓ | 0.34 | ¯ | 0.35 | ¯ | 0.57 | ¯ | 0.43 | ¯ | 0.56 | ¯ | 0.30 | ¯ |
| 18 | 6.29 | 196.0939 | [M-H]^-^ | C_10_H_15_NO_3_ | Metanephrine | - | 5.41 | ↑ | 1.44 | ¯ | 0.02 | ¯ | 3.03 | ¯ | 1.02 | ¯ | 0.47 | ¯ | 2.37 | ¯ |
| 19 | 0.71 | 114.0658 | [M+H]^+^ | C_4_H_7_N_3_O | Creatinine | Energy metabolism | 4.74 | ↓ | 5.37 | ↑ | 6.77 | ↑ | 0.14 | ¯ | 4.00 | ¯ | 9.74 | ↑ | 0.15 | ¯ |
| 20 | 0.61 | 124.0046 | [M-H]^-^ | C_2_H_7_NO_3_S | Taurine | Primary bile acid biosynthesis | 1.41 | ↓ | 0.59 | ¯ | 1.59 | ¯ | 1.16 | ¯ | 0.91 | ¯ | 1.22 | ¯ | 1.24 | ¯ |
| 21 | 1.78 | 137.0477 | [M+H]^+^ | C_5_H_4_N_4_O | Hypoxanthine | Purine metabolism | 0.51 | ↓ | 0.89 | ↑ | 1.43 | ¯ | 0.50 | ¯ | 1.81 | ↓ | 1.94 | ¯ | 2.08 | ↓ |
| 22 | 0.68 | 159.0459 | [M+H]^+^ | C_4_H_6_N_4_O_3_ | Allantoin |  | 0.64 | ↓ | 1.07 | - | 1.32 | - | 1.02 | - | 1.45 | - | 1.42 | - | 1.01 | - |
| 23 | 1.21 | 166.0729 | [M+H]^+^ | C6H7N5O | 7-Methylguanine | - | 0.21 | ↓ | 2.19 | - | 2.72 | - | 2.46 | - | 0.48 | - | 1.68 | - | 2.50 | - |
| 24 | 8.60 | 305.1756 | [M+H]^+^ | C_18_H_24_O_4_ | Latanoprost Lactone Diol | - | 2.33 | ↑ | 2.22 | ↓ | 1.77 | ↓ | 0.78 | - | 1.01 | ↓ | 2.37 | ↓ | 0.85 | - |
| 25 | 0.86 | 154.0983 | [M+H]^+^ | C_8_H_11_O_2_N | Dopamine | Phenylalanine metabolism | 2.09 | ↑ | 3.20 | ↑ | 1.71 | ↑ | 1.27 | - | 2.33 | ↑ | 1.16 | ↑ | 3.88 | ↑ |
| 26 | 1.47 | 112.0509 | [M+H]^+^ | C_21_H_28_O_5_ | Cortisone | - | 2.59 | ↓ | 1.32 | - | 0.20 | - | 1.51 | - | 1.48 | ↑ | 0.52 | - | 0.88 | - |
| 27 | 3.24 | 110.0596 | [M+H]^+^ | C_6_H_7_NO | Hydroxylaminobenzene | Aminobenzoate degradation | 1.79 | ↓ | 1.73 | ↓ | 2.53 | ↓ | 2.51 | ↓ | 1.92 | ↓ | 2.47 | ↓ | 2.35 | ↓ |

Note:（+）means positive ion mode; （-）means negative ion mode; “↑” means a higher level of metabolites, whereas “↓” represents a lower level of metabolites. “—” represents no statistically significant difference.

## Tables S15 Mass databases of 19 metabolites in hyperthyroidism rats

| NO. | RT(min) | Ion(m/z) | Ion mode | Elemental  composition | Identification | Related pathway | MO vs CON | | WD vs MO | | CPF vs MO | | VOF vs MO | | LAF vs MO | | OSF vs MO | | ATF vs MO | |
| --- | --- | --- | --- | --- | --- | --- | --- | --- | --- | --- | --- | --- | --- | --- | --- | --- | --- | --- | --- | --- |
|  |  |  |  |  |  |  | VIP | Trend | VIP | Trend | VIP | Trend | VIP | Trend | VIP | Trend | VIP | Trend | VIP | Trend |
| 1 | 5.01 | 181.0867 | [M+H]^+^ | C_6_H_12_O_6_ | Mannose | Fructose and mannose metabolism | 5.48 | ↓ | 1.50 | - | 1.90 | - | 1.51 | - | 0.60 | - | 1.60 | - | 1.88 | - |
| 2 | 2.89 | 206.0455(+) | [M+H]^+^ | C_10_H_7_NO_4_ | Xanthurenic acid | Tryptophan metabolism | 7.12 | ↑ | 0.65 | - | 1.96 | - | 1.60 | - | 4.28 | - | 3.84 | - | 3.30 | - |
| 3 | 3.20 | 190.0507(+) | [M+H]^+^ | C_10_H_7_NO_3_ | Kynurenic acid |  | 10.79 | ↑ | 0.07 | - | 1.03 | - | 1.72 | - | 2.50 | - | 4.56 | - | 5.11 | - |
| 4 | 0.69 | 90.0536 | [M+H]^+^ | C_3_H_7_NO_2_ | Alanine | Alanine, aspartate and glutamate metabolism | 1.50 | ↑ | 2.17 | ↑ | 0.72 | - | 0.14 | - | 1.97 | - | 3.77 | ↑ | 0.07 | - |
| 5 | 3.77 | 178.0496 | [M-H]^-^ | C_9_H_9_NO_3_ | Hippuric acid | Phenylalanline metabolism | 8.78 | ↑ | 5.20 | - | 6.19 | ↓ | 4.35 | - | 6.83 | ↓ | 7.94 | ↓ | 2.15 | - |
| 6 | 0.62 | 124.001 | [M-H]^-^ | C_12_H_20_O_4_ | Taurine | Primary bile acid biosynthesis | 1.73 | ↑ | 0.06 | - | 1.16 | - | 0.07 | - | 0.30 | - | 0.12 | - | 0.47 | - |
| 7 | 8.77 | 251.1294 | [M+H]^+^ | C_8_H_12_N O_6_P | Pyridoxine phosphate | Vitamin B6 metabolism | 2.42 | ↓ | 1.73 | - | 0.68 | - | 0.40 | - | 0.76 | - | 0.24 | - | 1.40 | - |
| 8 | 0.72 | 114.0657 | [M+H]^+^ | C_4_H_7_N_3_O | Creatinine | Arginine and proline metabolism | 9.77 | ↓ | 0.46 | - | 1.12 | - | 0.38 | - | 5.14 | - | 1.54 | - | 4.55 | - |
| 9 | 2.35 | 218.1018 | [M-H]^-^ | C_9_H_17_NO_5_ | Pantothenic acid | Vitamin digestion and absorption | 4.21 | ↑ | 3.27 | - | 4.57 | ↓ | 4.35 | ↓ | 4.36 | ↓ | 1.57 | - | 1.60 | - |
| 10 | 0.68 | 137.0711 | [M+H]^+^ | C_5_H_4_N_4_O | Hypoxanthine | Purine metabolism | 4.07 | ↓ | 0.33 | - | 4.01 | - | 0.06 | - | 0.10 | - | 0.59 | - | 3.29 | ↑ |
| 11 | 1.13 | 123.0527 | [M+H]^+^ | C_6_H_6_N_2_O | Picolinamide | - | 5.93 | ↑ | 1.83 | - | 0.65 | - | 1.22 | - | 7.83 | ↓ | 3.91 | - | 6.26 | ↓ |
| 12 | 5.74 | 220.1364 | [M+H]^+^ | C_13_H_17_NO_2_ | Cyclohexyl 2-aminobenzoate | - | 2.66 | ↑ | 2.66 | - | 3.50 | ↓ | 1.63 | ↓ | 3.44 | ↓ | 2.43 | - | 1.12 | - |
| 13 | 0.74 | 119.0311 | [M+HCOOH-H]^-^ | C_3_H_6_O_2_ | 3-Hydroxypropanal | Fatty acid degradation | 1.29 | ↓ | 1.06 | - | 1.41 | - | 0.70 | ↑ | 1.53 | ↑ | 1.33 | ↑ | 0.97 | - |
| 14 | 5.89 | 349.2388 | [M-H_2_O+H]^+^ | C_21_H_34_O_5_ | Cortolone | Steroid synthesis biosynthesis | 1.58 | ↑ | 0.98 | - | 0.41 | - | 0.88 | - | 0.89 | - | 0.72 | - | 0.69 | - |
| 15 | 6.31 | 349.2379 | [M+H]^+^ | C_21_H_32_O_4_ | 3β, 17α, 21-trihydroxy-pregnenone |  | 3.32 | ↑ | 1.25 | - | 1.75 | - | 0.41 | - | 1.27 | - | 1.54 | - | 0.89 | - |
| 16 | 1.02 | 273.1094 | [M-H_2_O+H]^+^ | C_10_H_18_N_4_O_6_ | Arginosuccinic acid | - | 4.65 | ↓ | 0.55 | ↓ | 0.10 | - | 0.58 | - | 0.26 | - | 0.34 | - | 0.75 | ↓ |
| 17 | 6.29 | 196.0961 | [M-H]^-^ | C_10_H_15_NO_3_ | Metanephrine | - | 3.71 | ↓ | 1.06 | - | 0.30 | ↑ | 0.94 | - | 0.43 | ↑ | 1.17 | ↑ | 0.96 | ↑ |
| 18 | 2.22 | 261.1405 | [M+H]^+^ | C_11_H_20_N_2_O_5_ | L-gamma-glutamyl-L-isoleucine | - | 1.18 | ↑ | 1.34 | - | 0.16 | - | 0.86 | - | 0.92 | - | 0.57 | - | 1.11 | ↓ |
| 19 | 0.70 | 132.077 | [M+H]^+^ | C_4_H_9_N_3_O_2_ | Creatine | Arginine and proline metabolism | 1.69 | ↑ | 4.66 | - | 2.86 | - | 0.13 | - | 4.33 | - | 6.48 | - | 0.35 | 0.70 |

Note:（+）means positive ion mode; （-）means negative ion mode; “↑” means a higher level of metabolites, whereas “↓” represents a lower level of metabolites. “—” represents no statistically significant difference.
