# Supplementary material for: Application of Proteomics and Metabonomics to Reveal the Molecular Basis of Atractylodis Macrocephalae Rhizome for Ameliorating Hypothyroidism Instead of Hyperthyroidism
Source: Front Pharmacol. 2021 Apr 20;12:664319. doi: 10.3389/fphar.2021.664319 (PMC8095350; doi:10.3389/fphar.2021.664319)
Supplement: Supplementary file 3 [file DataSheet1.docx]

**Chemicals and reagents**

A UV-2100 spectrophotometer was purchased from Unico Co. (Shanghai, China). A Microplate Reader was purchased from Kate Biological Medical Electronics Technology Co. Ltd. (Shenzhen, China). Normal saline was obtained from Kelun Pharmaceutical Ltd. (Heilongjiang, China). PTU (Batch No.BCBM4329V) was obtained from Sigma-Aldrich (St. Louis, MO, USA). T_3_ assay kit (Batch No. 20160901), T_4_ assay kit (Batch No.20160903), Na^+^-K^+^-ATPase assay kit (Batch No. 20160903), Fatty acid synthase assay kit (Batch No.20161203) and Peroxisomal bifunctional enzyme assay kit (Batch No.20161209) were purchased from Nanjing Jian Cheng Bioengineering Institute (Nanjing, China). HPLC grade acetonitrile, methanol and MS-grade formic acid were purchased from Merck (Merck, Germany). Ultra-pure water was treated with a Milli-Q water purification system (Millipore, France) and other reagents were analytical grade. An iTRAQ 8-plex application kit was purchased from Applied Biosystems (Sigma, USA). Strong cation exchange (SCX) and C_18_ solid-phase extraction (SPE) cartridges were purchased from Supelco (PA, USA). Triple-TOF 5600 system (AB SCIEX, Massachusetts, USA).

AMR was collected in *Yuqian*, Zhejiang province, Oct.2015 which is the geo-authentic producing area. The plant materials were identified by Professor Wang Bing (Liaoning University of Traditional Chinese Medicine) as rhizomes of *Atractylodes macrocephala* Koidz.

Rats for experiment were purchased from the laboratory animal center of Changsheng Bio-Technique Co. Ltd. (Benxi, Liaoning, China), qualified no. SCXK 2010-0001. Animals were kept in an air conditioned room (22°C; relative humidity, 55%) and fed ad libitum with standard feed and water in the course of the study. The investigation conformed to the Guide for the Care and Use of Laboratory Animals published by the US National Institutes of Health (NIH Publication No. 85-23, revised 1996). The study protocol was approved by the ethics regulations of Liaoning University of Traditional Chinese Medicine, China (131/2010).

**The preparation of splitted fractions and component analysis**

According to the polarity of components of AMR, the water decoction of AMR was split as follow. Briefly, take a suitable amount of AMR and add 12 times the amount of water, reflux extraction by essential oil extractor for 2 times, each 1h, to obtain volatile oil fraction (VOF) and water decoction (WD). The water decoction of AMR was concentrated and Crude Polysaccharides Faction (CPF) was obtained by regulate the alcohol concentration to 75%, precipitate, lyophilization. Further, the supernatant was evaporated on water bath to and the resulting water layer was extracted eight times with 60~90EC petroleum ether until there is no color in layer of petroleum ether to give Petroleum Ether Fraction (LAF). The resulting water layer was subjected to a column of macroporous adsorption resin D101, washing with distilled water, 60% ethanol and 80% ethanol successively. The water eluate was concentrated at 50EC and lyophilized to give Water Eluted Fraction (OSF) and the 60 and 80% ethanol elutes were collected and evaporated in vacuum to give the Alcohol Eluted Fraction (ATF). Five fractions of AMR were obtained as follows: volatile oil fraction (VOF, yield, 0.858±0.102%), lactones fraction (LAF, yield, 0.204±0.002%), atractyloside fraction (ATF, yield, 0.858±0.102%), oligosaccharide fraction (OSF, yield, 32.7±2.0%), crud polysaccharide fraction (CPF, yield, 36.7±2.27%).
